# A Patient‐Derived Organoid Biobank of Adamantinomatous Craniopharyngioma as a Platform for Drug Discovery

**DOI:** 10.1002/advs.202503924

**Published:** 2025-11-18

**Authors:** Huarong Zhang, Chaohu Wang, Jun Fan, Zexin Chen, Haoying Yu, Yawen Bai, Tingcheng Zhang, Qianchao Zhu, Yiwen Feng, Peirong Niu, Jiaqian Chen, Liping Yang, Xueying Li, Lei Yu, Songtao Qi, Yi Liu

**Affiliations:** ^1^ Department of Neurosurgery Nanfang Hospital Southern Medical University Guangzhou Guangdong 510515 China; ^2^ Guangdong Research Center of Organoid Engineering and Technology Guangzhou Guangdong 510535 China; ^3^ Department of Pharmacy Shenzhen Qianhai Taikang Hospital Shenzhen Guangdong 518000 China

**Keywords:** adamantinomatous craniopharyngioma, ceritinib, drug screening, IGF‐1R/PI3K/AKT/GSK3β/β‐catenin signaling axis, insulin‐like growth factor 1 receptor, patient‐derived organoids

## Abstract

Adamantinomatous craniopharyngioma (ACP), a benign yet clinically challenging neoplasm situated in sellar‐suprasellar region, frequently causes hypothalamic dysfunction. Despite the identification of molecular alterations in ACP, the absence of robust research models has impeded the advancement of targeted therapies. Herein, the development of a large‐scale ACP biobank comprising 54 patient‐derived organoids (PDOs) is presented, achieves with a notable 90% success rate. Comprehensive characterization using hematoxylin and eosin (H&E) staining, immunofluorescence staining, and whole‐exome sequencing (WES) demonstrates that PDOs faithfully recapitulate key histoarchitectural features, molecular marker expression profiles, and somatic mutational landscapes of corresponding parental tumors. Drug sensitivity screening reveals diverse responses of PDOs to the drugs tested, with Ceritinib exhibiting potent and consistent anti‐tumor activity across seventeen PDOs evaluated. Further mechanistic investigations utilizing RNA transcriptomic sequencing have elucidated that Ceritinib inhibits PDO growth by downregulating the IGF‐1R/PI3K/AKT/GSK‐3β/β‐catenin signaling axis. Additionally, a retrospective analysis of two Ceritinib‐treated clinical cases reveals tumor growth with treatment before any possible therapeutic effects are observed, highlighting the need for caution and careful monitoring in treating ACP patients. Collectively, these findings demonstrate that ACP PDOs effectively preserve the biological characteristics of original tumors, thereby providing a valuable platform for developing precision therapies for ACP patients.

## Graphical abstract

This study successfully establishes adamantinomatous craniopharyngioma (ACP) patient‐derived organoids (PDOs) that preserve the histopathological and genetic features of the original tumors. Through drug sensitivity assays and subsequent mechanistic analyses, the study demonstrates that Ceritinib exerts its inhibitory effects on ACP PDO growth by downregulating the IGF‐1R/PI3K/AKT/GSK‐3β/β‐catenin axis. Concurrently, clinical case studies provide preliminary, hypothesis‐generating evidence suggesting that Ceritinib may have therapeutic potential against ACP.

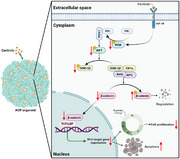

## 1 Introduction

Adamantinomatous craniopharyngioma (ACP), the predominant subtype of craniopharyngioma (CP), is a rare intracranial neoplasm that, despite its histologically benign classification, exhibits clinically aggressive behavior. It originates in the sellar‐suprasellar region and presents significant clinical challenges due to its proximity to vital structures such as the hypothalamus, pituitary stalk, optic nerve, optic chiasm, and internal carotid artery.^[^ ^1^, ^2^ ^]^ ACP is the most prevalent pituitary tumor in pediatric populations, representing 5%–11% of intracranial tumors in this demographic.^[^ ^3^, ^4^, ^5^ ^]^ Although survival rates are generally favorable, affected individuals frequently experience a reduced quality of life due to functional impairments associated with both the tumor and its treatment.^[^ ^3^, ^4^, ^5^ ^]^ Current therapeutic approaches for ACP encompass surgical resection, which may be either gross‐total or subtotal, radiotherapy, radiosurgery using gamma knife technology, stereotactic intracavitary irradiation, and cyst aspiration.^[^ ^2^, ^6^ ^]^ Recent investigations indicate that intracystic administration of interferon‐alpha (IFNα) may retard disease progression, possibly due to its anti‐inflammatory properties, and could present a safer alternative compared to other treatment modalities in pediatric ACP patients.^[^ ^7^ ^]^ In alignment with these findings, systemic administration of interleukin‐6 (IL‐6) inhibitors has demonstrated promising outcomes in the management of cystic ACP.^[^ ^8^ ^]^ Moreover, the potential application of immunotherapy in the treatment of ACP is under exploration; however, it necessitates further preclinical assessment.^[^ ^9^, ^10^, ^11^, ^12^ ^]^

Genetic analyses have demonstrated that nearly all cases of ACP exhibit activating somatic mutations in exon 3 of the *CTNNB1* gene, which encodes β‐catenin. This mutation results in the abnormal nuclear accumulation of β‐catenin, leading to the activation of Wnt target genes.^[^ ^13^, ^14^, ^15^ ^]^ Furthermore, a range of other molecular alterations have been identified in ACP tissues, including the dysregulation of signaling pathways such as sonic hedgehog (SHH), epidermal growth factor receptor (EGFR), mitogen‐activated protein kinase (MAPK), vascular endothelial growth factor (VEGF), Axin 2, Bone Morphogenetic Protein 4 (BMP4), transforming growth factor‐β (TGF‐β), and fibroblast growth factor (FGF).^[^ ^16^, ^17^, ^18^, ^19^, ^20^, ^21^, ^22^ ^]^ Emerging evidence also suggests a link between the expression of insulin‐like growth factor 1 receptor (IGF‐1R) and pro‐inflammatory signaling, as well as the maintenance of stemness in ACP, identifying it as a potential therapeutic target.^[^ ^23^ ^]^ Insulin‐like growth factor 1 (IGF‐1) has been demonstrated to enhance growth in CP cell cultures characterized by elevated expression of IGF‐1R.^[^ ^24^, ^25^ ^]^ Nevertheless, the scarcity of well‐established ACP cell lines and pertinent animal models has impeded functional studies aimed at determining whether these molecular changes constitute viable therapeutic targets. Notably, IGF‐1R, is under investigation as a therapeutic target across various cancers, with clinical trials currently underway to evaluate the efficacy of IGF‐1R inhibitors in diverse tumor types.

In recent years, 3D culture systems, known as “organoids”, have emerged as influential tools in cancer research.^[^ ^26^, ^27^ ^]^ Patient‐derived organoids (PDOs) have been effectively utilized to model a variety of cancers, including pancreatic cancer, hepatocellular carcinoma (HCC), lung cancer, bladder cancer, gastric cancer (GC), colorectal cancer (CRC), and glioma.^[^ ^28^, ^29^, ^30^, ^31^, ^32^, ^33^, ^34^ ^]^ PDOs have proven to be robust preclinical models for personalized therapy testing and tumor biology analyses. PDOs accurately replicate key characteristics of the tissues from which they are derived, such as differentiation capacity toward tissue‐specific lineages and stem cell self‐renewal.^[^ ^26^, ^27^ ^]^ Compared to traditional tumor cell lines, PDOs are regarded as superior models for emulating the characteristics of native tumors, thus making them ideal for the identification of potential therapeutic agents.^[^ ^30^, ^31^ ^]^ Additionally, the establishment of PDOs has been reported to be more efficient than that of tumor cell lines or patient‐derived xenograft (PDX) models.^[^ ^35^ ^]^ Consequently, PDOs represent a promising and reliable platform for both basic and preclinical research in ACP.

In this study, we successfully established 54 PDOs from 60 ACP tissue samples, achieving a commendable success rate of 90.0%. These PDOs demonstrated morphological and molecular characteristics that closely mirrored those of their respective parental tumors. Through drug sensitivity assays and subsequent mechanistic investigations utilizing these PDOs, Ceritinib, an inhibitor of the IGF‐1R, was identified as a potent agent that suppresses PDO growth by downregulating the IGF‐1R/PI3K/AKT/GSK‐3β/β‐catenin signaling axis. Preliminary clinical administration results of Ceritinib suggest its potential anti‐tumor efficacy in ACP. Our findings propose that IGF‐1R is a viable therapeutic target for ACP and underscore the utility of ACP PDOs as a valuable platform for preclinical research and the advancement of precision medicine.

## 2 Results

### 2.1 Patient Samples and Clinical Characteristics

Tumor tissue specimens from 60 patients, histologically diagnosed with ACP, were surgically resected and collected at the Department of Neurosurgery, Nanfang Hospital, between December 2021 and July 2024 (**Table** **1**). The cohort comprised 27 adolescents and 33 adults, including 24 males and 36 females. Among these patients, 45 were newly diagnosed and treatment‐naïve, while 15 presented with recurrent tumors. Of the recurrent cases, 13 had previously undergone surgical resection, 1 had received γ‐knife radiosurgery, and 1 had been treated with a combination of surgery, γ‐knife, and chemotherapy. Hormonal disorders were identified in 58 patients, whereas 2 patients exhibited normal hormone levels. The 60 tissue samples included 47 solid‐cystic tumors, 11 cystic tumors, and 2 solid tumors. Calcification was detected in 59 tumor tissue samples, with only 1 sample lacking significant calcification (Figure S1A, Supporting Information). Comprehensive baseline characteristics are detailed in Table 1. Histopathological diagnoses were validated by an experienced neuropathologist.

**Table 1:** Clinicopathologic Characteristics of 60 Patient‐derived ACP Organoids and Corresponding Parental Tumors.

No.	Organoid No.	Patient No.	Gender	Age (years)	Subtype	Prior treatment received	Presentation	Tumor morphology	Calcification	Size (cm)	Hormone disorders	Acqusition method	Qrganoid establishment
1	PDO_01	ACP_01	M	2	ACP	None	Newly diagnosed	Solid‐cystic	Yes	2.3 × 2.4 × 2.7	Yes	Surgery	Success
2	PDO_02	ACP_02	F	14	ACP	Surgery	Recurrent	Solid‐cystic	Yes	5.0 × 4.2 × 3.0	Yes	Surgery	Success
3	PDO_03	ACP_03	M	5	ACP	None	Newly diagnosed	Solid‐cystic	Yes	2.7 × 3.2 × 3.9	Yes	Surgery	Success
4	PDO_04	ACP_04	F	4	ACP	None	Newly diagnosed	Solid‐cystic	Yes	5.5 × 2.8 × 5.6	Yes	Surgery	Success
5	PDO_05	ACP_05	M	36	ACP	None	Newly diagnosed	Solid‐cystic	Yes	4.1 × 3.1 × 3.5	Yes	Surgery	Success
6	PDO_06	ACP_06	M	6	ACP	Surgery	Recurrent	Solid	Yes	0.7 × 0.9 × 1.6	Yes	Surgery	Success
7	PDO_07	ACP_07	M	5	ACP	None	Newly diagnosed	Cystic	Yes	2.7 × 3.7 × 3.7	Yes	Surgery	Success
8	PDO_08	ACP_08	M	59	ACP	None	Newly diagnosed	Cystic	Yes	1.4 × 1.5 × 1.5	Yes	Surgery	Failed
9	PDO_09	ACP_09	M	17	ACP	Surgery	Recurrent	Solid‐cystic	Yes	6.0 × 3.6 × 2.6	Yes	Surgery	Success
10	PDO_10	ACP_10	M	8	ACP	None	Newly diagnosed	Solid‐cystic	Yes	4.6 × 3.7 × 3.0	Yes	Surgery	Success
11	PDO_11	ACP_11	M	5	ACP	None	Newly diagnosed	Cystic	Yes	3.9 × 4.3 × 7.8	Yes	Surgery	Success
12	PDO_12	ACP_12	F	8	ACP	None	Newly diagnosed	Cystic	Yes	4.1 × 1.7 × 3.1	Yes	Surgery	Success
13	PDO_13	ACP_13	M	48	ACP	Surgery	Recurrent	Solid‐cystic	Yes	3.1 × 3.3 × 2.9	Yes	Surgery	Success
14	PDO_14	ACP_14	M	1	ACP	None	Newly diagnosed	Cystic	Yes	3.5 × 3.5 × 3.1	Yes	Surgery	Success
15	PDO_15	ACP_15	M	39	ACP	None	Newly diagnosed	Solid‐cystic	Yes	2.4 × 1.6 × 2.6	Yes	Surgery	Success
16	PDO_16	ACP_16	M	57	ACP	Surgery	Recurrent	Solid‐cystic	Yes	4.1 × 4.7 × 7.2	Yes	Surgery	Success
17	PDO_17	ACP_17	M	9	ACP	None	Newly diagnosed	Solid‐cystic	Yes	2.5 × 1.9 × 2.1	Yes	Surgery	Success
18	PDO_18	ACP_18	F	33	ACP	Surgery+γ‐knife+ chemotherapy	Recurrent	Solid‐cystic	Yes	4.5 × 3.9 × 3.3	Yes	Surgery	Success
19	PDO_19	ACP_19	M	5	ACP	Surgery	Recurrent	Solid‐cystic	Yes	3.7 × 3.2 × 3.6	Yes	Surgery	Success
20	PDO_20	ACP_20	F	33	ACP	None	Newly diagnosed	Solid‐cystic	No	1.9 × 1.8 × 2.2	Yes	Surgery	Success
21	PDO_21	ACP_21	M	6	ACP	None	Newly diagnosed	Solid‐cystic	Yes	4.5 × 4.1 × 5.3	Yes	Surgery	Success
22	PDO_22	ACP_22	M	53	ACP	None	Newly diagnosed	Solid‐cystic	Yes	2.4 × 2.0 × 2.3	Yes	Surgery	Success
23	PDO_23	ACP_23	M	61	ACP	Surgery	Recurrent	Solid‐cystic	Yes	2.9 × 1.5 × 4.5	Yes	Surgery	Success
24	PDO_24	ACP_24	F	4	ACP	None	Newly diagnosed	Cystic	Yes	4.7 × 3.5 × 3.7	Yes	Surgery	Success
25	PDO_25	ACP_25	M	32	ACP	None	Newly diagnosed	Cystic	Yes	2.2 × 2.2 × 1.9	Yes	Surgery	Success
26	PDO_26	ACP_26	F	50	ACP	None	Newly diagnosed	Solid‐cystic	Yes	3.3 × 2.8 × 2.8	Yes	Surgery	Failed
27	PDO_27	ACP_27	F	7	ACP	None	Newly diagnosed	Solid‐cystic	Yes	2.6 × 1.8 × 4.2	Yes	Surgery	Success
28	PDO_28	ACP_28	F	48	ACP	None	Newly diagnosed	Solid‐cystic	Yes	1.9 × 1.6 × 1.8	Yes	Surgery	Success
29	PDO_29	ACP_29	M	68	ACP	Surgery	Recurrent	Solid‐cystic	Yes	3.2 × 3.4 × 3.2	Yes	Surgery	Success
30	PDO_30	ACP_30	F	54	ACP	None	Newly diagnosed	Solid‐cystic	Yes	2.2 × 2.9 × 4.3	Yes	Surgery	Success
31	PDO_31	ACP_31	F	71	ACP	None	Newly diagnosed	Solid‐cystic	Yes	2.4 × 2.0 × 3.0	Yes	Surgery	Success
32	PDO_32	ACP_32	F	37	ACP	None	Newly diagnosed	Cystic	Yes	3.6 × 4.0 × 3.4	Yes	Surgery	Success
33	PDO_33	ACP_33	M	30	ACP	None	Newly diagnosed	Solid‐cystic	Yes	3.7 × 2.9 × 4.3	Yes	Surgery	Success
34	PDO_34	ACP_34	M	12	ACP	None	Newly diagnosed	Solid‐cystic	Yes	2.4 × 3.0 × 3.6	Yes	Surgery	Success
35	PDO_35	ACP_35	M	20	ACP	None	Newly diagnosed	Solid‐cystic	Yes	2.7 × 1.9 × 2.5	Yes	Surgery	Success
36	PDO_36	ACP_36	M	55	ACP	None	Newly diagnosed	Solid‐cystic	Yes	3.4 × 3.3 × 3.4	Yes	Surgery	Success
37	PDO_37	ACP_37	M	18	ACP	Surgery	Recurrent	Solid‐cystic	Yes	7.0 × 5.3 × 4.9	Yes	Surgery	Success
38	PDO_38	ACP_38	F	38	ACP	Surgery	Recurrent	Solid‐cystic	Yes	2.9 × 3.3 × 4.4	Yes	Surgery	Success
39	PDO_39	ACP_39	M	48	ACP	None	Newly diagnosed	Cystic	Yes	3.4 × 1.8 × 1.9	Yes	Surgery	Success
40	PDO_40	ACP_40	M	20	ACP	None	Newly diagnosed	Solid‐cystic	Yes	5.5 × 4.2 × 6.0	Yes	Surgery	Success
41	PDO_41	ACP_41	M	4	ACP	None	Newly diagnosed	Solid‐cystic	Yes	2.7 × 2.1 × 3.2	Yes	Surgery	Success
42	PDO_42	ACP_42	F	25	ACP	None	Newly diagnosed	Solid‐cystic	Yes	2.3 × 2.8 × 4.5	Yes	Surgery	Success
43	PDO_43	ACP_43	M	25	ACP	None	Newly diagnosed	Solid‐cystic	Yes	6.3 × 4.0 × 4.0	Yes	Surgery	Success
44	PDO_44	ACP_44	M	16	ACP	None	Newly diagnosed	Solid‐cystic	Yes	3.8 × 3.1 × 3.3	Yes	Surgery	Success
45	PDO_45	ACP_45	F	4	ACP	None	Newly diagnosed	Solid‐cystic	Yes	5.4×4.0×4.6	Yes	Surgery	Success
46	PDO_46	ACP_46	F	38	ACP	None	Newly diagnosed	Solid‐cystic	Yes	2.5 × 2.4 × 2.4	No	Surgery	Success
47	PDO_47	ACP_47	F	2	ACP	None	Newly diagnosed	Cystic	Yes	2.0 × 1.5 × 3.3	No	Surgery	Success
48	PDO_48	ACP_48	M	37	ACP	None	Newly diagnosed	Solid‐cystic	Yes	6.2 × 4.6 × 4.5	Yes	Surgery	Failed
49	PDO_49	ACP_49	F	13	ACP	None	Newly diagnosed	Solid‐cystic	Yes	4.0 × 3.5 × 3.1	Yes	Surgery	Success
50	PDO_50	ACP_50	F	5	ACP	None	Newly diagnosed	Solid‐cystic	Yes	2.5 × 3.1 × 3.6	Yes	Surgery	Failed
51	PDO_51	ACP_51	M	24	ACP	Surgery	Recurrent	Solid‐cystic	Yes	2.3 × 1.6 × 2.2	Yes	Surgery	Success
52	PDO_52	ACP_52	F	4	ACP	None	Newly diagnosed	Solid‐cystic	Yes	2.1 × 3.2 × 2.3	Yes	Surgery	Success
53	PDO_53	ACP_53	F	41	ACP	Surgery	Recurrent	Solid‐cystic	Yes	6.6 × 5.2 × 5.8	Yes	Surgery	Success
54	PDO_54	ACP_54	M	28	ACP	None	Newly diagnosed	Solid‐cystic	Yes	4.1 × 3.3 × 3.1	Yes	Surgery	Success
55	PDO_55	ACP_55	M	55	ACP	γ‐knife	Recurrent	Cystic	Yes	5.8 × 3.9 × 6.0	Yes	Surgery	Success
56	PDO_56	ACP_56	F	17	ACP	None	Newly diagnosed	Solid‐cystic	Yes	4.2 × 3.8 × 3.9	Yes	Surgery	Success
57	PDO_57	ACP_57	F	44	ACP	None	Newly diagnosed	Solid‐cystic	Yes	4.1 × 2.6 × 2.9	Yes	Surgery	Success
58	PDO_58	ACP_58	F	54	ACP	None	Newly diagnosed	Solid‐cystic	Yes	2.5 × 2.3 × 2.7	Yes	Surgery	Failed
59	PDO_59	ACP_59	M	23	ACP	Surgery	Recurrent	Solid	Yes	6.2 × 3.6 × 3.2	Yes	Surgery	Success
60	PDO_60	ACP_60	M	6	ACP	None	Newly diagnosed	Solid‐cystic	Yes	1.7 × 1.7 × 2.9	Yes	Surgery	Failed

### 2.2 Establishment of Patient‐Derived ACP Organoid Models and Biobank

**Figure 1:**
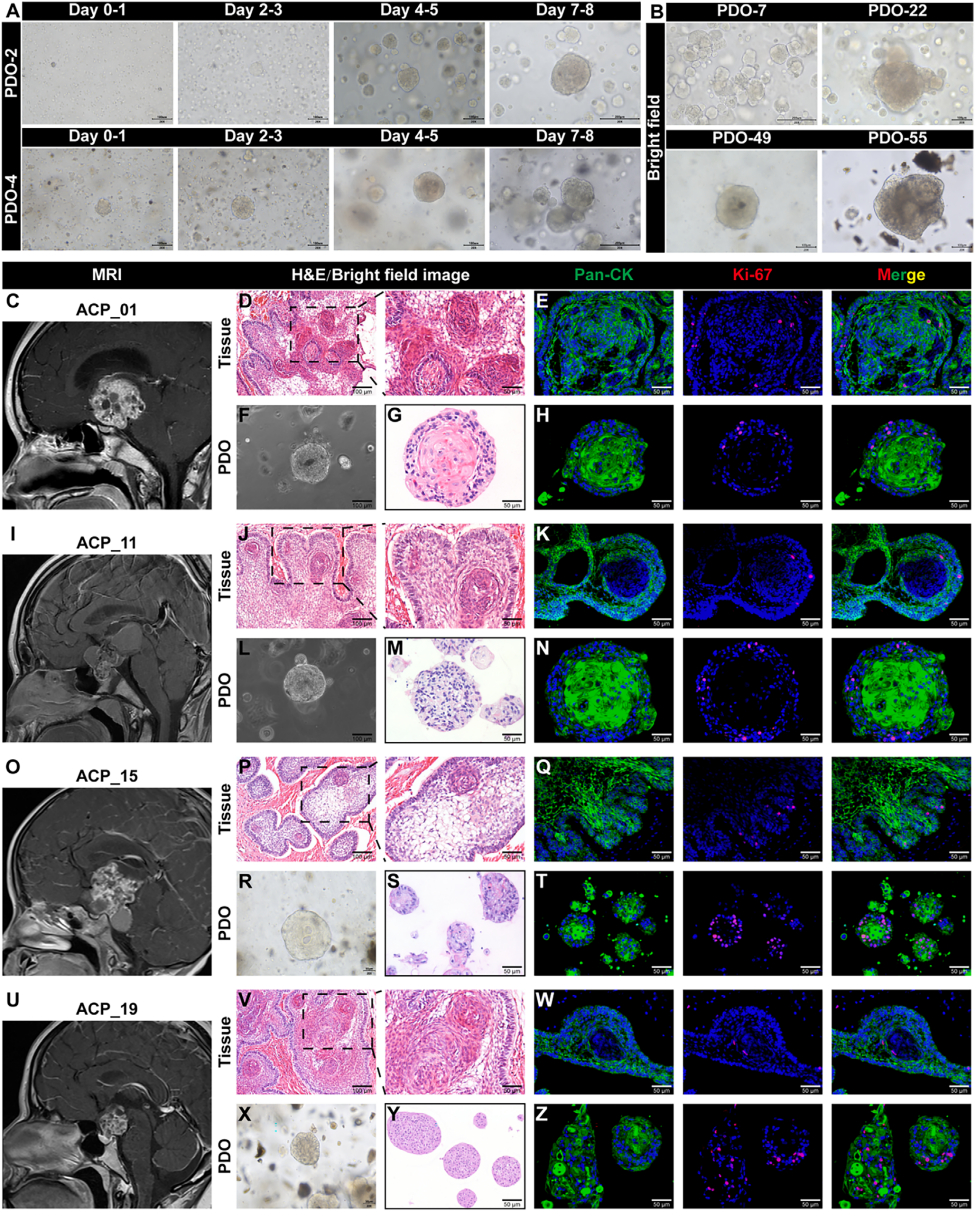
Establishment of a patient‐derived organoid (PDO) biobank for adamantinomatous craniopharyngioma (ACP). A) Representative bright‐field micrographs of ACP PDOs at various time points demonstrate the progression of ACP PDOs. The organoid cultures were derived from PDO_2 and PDO_4, respectively. B) Representative bright‐field micrographs of ACP organoid models generated from PDO_7, PDO_22, PDO_49, and PDO_55, respectively. C, I, O, U) Representative sagittal contrast‐enhanced T1‐weighted magnetic resonance (MR) images acquired preoperatively from patients ACP_01 C), ACP_11 I), ACP_15 O), and ACP_19 U), respectively. D, J, P, V) Representative hematoxylin and eosin (H&E) stained sections obtained postoperatively from samples of patients ACP_01 (D), ACP_11 (J), ACP_15 (P), and ACP_19 (V), respectively. Boxed area is enlarged and presented on the right. E, K, Q, W) Representative dual immunofluorescence images of Pan‐CK and Ki‐67 in patient samples ACP_01 E), ACP_11 K), ACP_15 Q), and ACP_19 W), respectively. F, G, L, M, R, S, X, Y) Representative bright‐field images F, L, R, X) and H&E staining G, M, S, Y) of PDOs derived from patient samples ACP_01, ACP_11, ACP_15, and ACP_19, respectively. H,N,T,Z) Representative dual immunofluorescence images of Pan‐CK and Ki‐67 in PDO samples PDO_01, PDO_11, PDO_15, and PDO_19, respectively.

Patient‐derived organoids (PDOs) from ACP were established in accordance with the protocol outlined in the “Experimental Section”. We successfully generated 54 distinct ACP organoid lines from 60 ACP tissue samples, achieving an overall success rate of 90.0% (Table 1). The PDOs typically emerged within 1 to 2 days post‐seeding in Matrigel and consistently self‐organized into spherical structures. Over time, the ACP PDOs increased in size and were passaged every 7–10 days (**Figure** **1**A). Although ACP PDOs from different patients exhibited similar spherical morphologies, they demonstrated significant variations in size under 3D culture conditions (Figure 1B). Instances of organoid culture failure, as documented in some cases (Figure S1B, Supporting Information), were primarily attributed to two factors: excessive calcification of tumor tissue and insufficient tumor parenchyma, particularly in cystic ACP. To establish a biobank of ACP PDOs, it is often necessary for these PDOs to undergo cryopreservation. In order to evaluate the impact of cryopreservation on the viability and biological characteristics of the PDOs, we conducted a comparative analysis of the number, size, and proliferation index (as indicated by Ki‐67 expression) of ACP PDOs before and after the cryopreservation process. Our findings demonstrated that cryopreservation did not result in significant alterations in Ki‐67 expression or organoid growth (*P* > 0.05) (Figure S1C–F, Supporting Information). Consequently, ACP PDOs can be effectively recovered from cryopreserved stocks and subsequently passaged in Matrigel. In summary, we have successfully established a biobank of ACP PDOs, which serves as a valuable resource for fundamental research on ACP.

### 2.3 PDO Models of ACP Recapitulate the Histopathologic Characteristics of Parental Tumors

**Figure 2:**
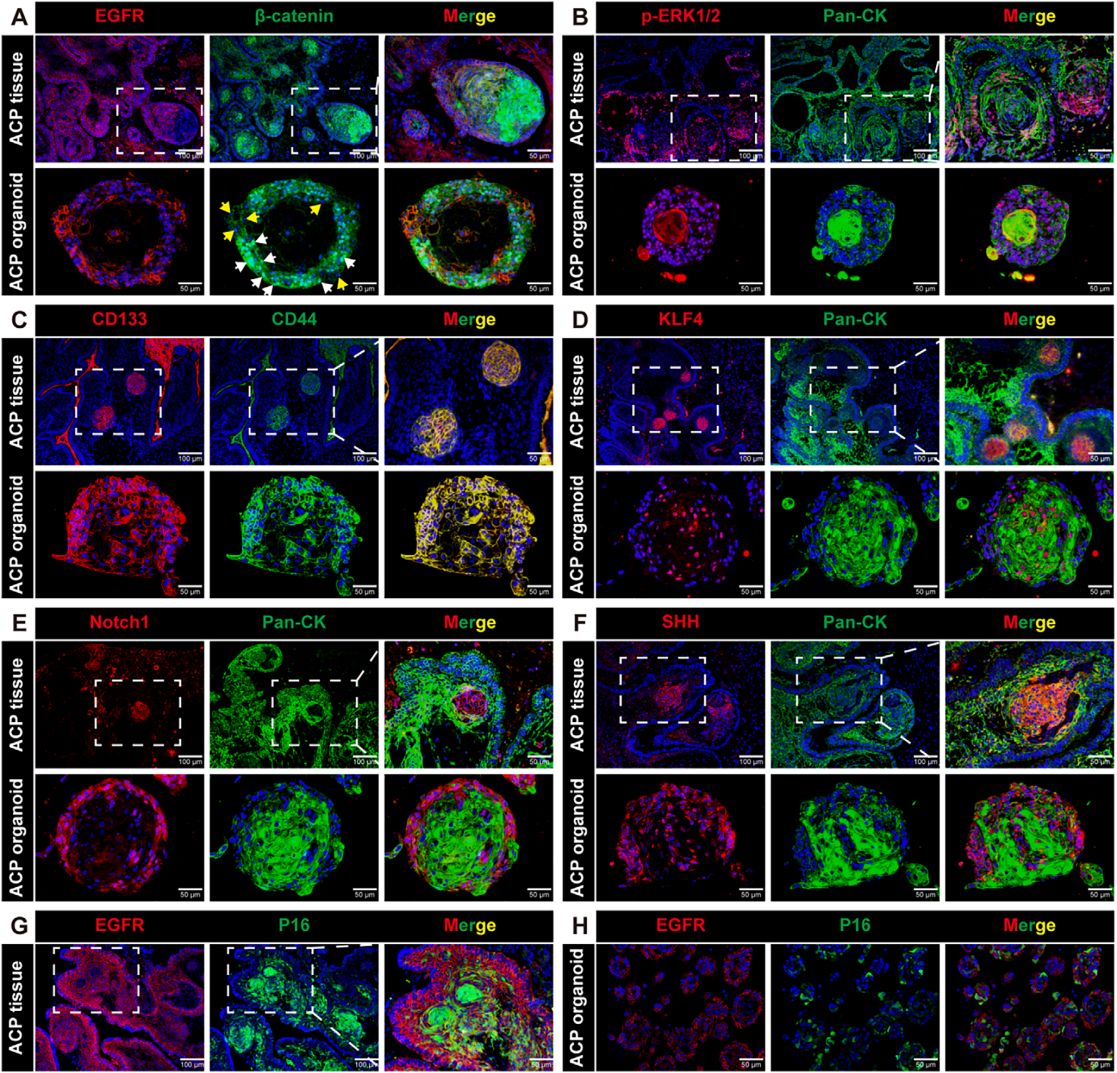
Established ACP organoid models accurately replicate the histopathological characteristics of corresponding parental tumors. A) Immunofluorescence staining of ACP tissue samples (upper panel) and ACP PDO samples (lower panel), representative images of β‐catenin and epidermal growth factor receptor (EGFR) staining. Boxed area is enlarged and presented on the right. White arrows denote cells with distinct nuclear localization, while yellow arrows denote cells with predominant membrane localization. B) Immunofluorescence staining of ACP tissue samples (upper panel) and ACP PDO samples (lower panel), representative images of Pan‐CK and phosphorylated‐ERK1/2 (p‐ERK1/2) staining. Boxed area is enlarged and presented on the right. C) Immunofluorescence staining of ACP tissue samples (upper panel) and ACP PDO samples (lower panel), representative images of CD44 and CD133 staining. Boxed area is enlarged and presented on the right. D) Immunofluorescence staining of ACP tissue samples (upper panel) and ACP PDO samples (lower panel), representative images of Pan‐CK and Kruppel‐like factor 4 (KLF4) staining. Boxed area is enlarged and presented on the right. E) Immunofluorescence staining of ACP tissue samples (upper panel) and ACP PDO samples (lower panel), representative images of Pan‐CK and Notch1 staining. Boxed area is enlarged and presented on the right. F) Immunofluorescence staining of ACP tissue samples (upper panel) and ACP PDO samples (lower panel), representative images of Pan‐CK and Sonic Hedgehog (SHH) staining. Boxed area is enlarged and presented on the right. G) Immunofluorescence staining of ACP tissue samples, representative images of EGFR and P16 staining. Boxed area is enlarged and presented on the right. H) Immunofluorescence staining of ACP PDO samples, representative images of EGFR and P16 staining.

To assess whether ACP PDOs retained the histological characteristics of their corresponding parental tumors, we conducted hematoxylin and eosin (H&E) staining and immunofluorescence analysis to compare the morphological features of four pairs of ACP PDOs and their original tumors. As illustrated in Figure 1C–Z, ACP PDOs demonstrated histological patterns akin to those of the originating tumor tissues. Notably, structures resembling wet keratin and/or ghost cells, which are commonly observed in ACP tissue samples, were also identified in ACP PDOs, as indicated by H&E staining (Figure S2A‐D, Supporting Information). Additionally, immunofluorescence staining revealed positive expression of the keratinized epithelial cell marker Pan‐cytokeratin (Pan‐CK) in ACP PDOs, with Ki‐67 proliferation index expression levels comparable to those observed in their parental tumors (Figure 1E,H,K,N,Q,T,W,Z). We further conducted an in‐depth analysis of the expression profiles of various biomarkers in ACP PDOs and their corresponding ACP tissues. The expression patterns of these biomarkers were largely conserved in ACP PDOs relative to the original tumors. Notably, immunostaining demonstrated a heterogeneous pattern of β‐catenin localization within ACP PDOs. In alignment with primary ACP tumors, a subset of epithelial whorls exhibited distinct nuclear translocation of β‐catenin, whereas in other cells, β‐catenin predominantly localized at the cell membrane (**Figure** **2**A). Additionally, ACP PDOs displayed positive expression of the epidermal growth factor receptor (EGFR), and phosphorylated ERK1/2 (p‐ERK1/2), suggesting activation of the EGFR and p42/44 MAPK signaling pathways, consistent with observations in the parental tumors (Figure 2A,B). Furthermore, the expression levels of stem cell‐associated markers, including CD44, CD133, Kruppel‐like factor 4 (KLF4), Notch1, and Sonic Hedgehog (SHH), were comparable between ACP PDOs and the matched tumor samples (Figure 2C–F). Additionally, the expression of P16 was consistent between the PDOs and the tumor tissues (Figure 2G,H).

The composition of ACP predominantly consists of three distinct epithelial cell types: whorl‐like epithelium (WE), keratinized‐like epithelium (KE), and palisade‐like epithelium (PE). To further elucidate the cellular composition within ACP PDOs, we performed transcriptomic sequencing on four ACP PDO cases, each with three biological replicates. This data was integrated with existing single‐cell RNA sequencing (scRNA‐seq) data from ACP using deconvolution techniques, specifically Cell‐type Identification By Estimating Relative Subsets Of RNA Transcripts (CIBERSORT) and MUlti‐Subject Single Cell deconvolution (MuSiC). Both deconvolution analyses revealed that WE is the predominant cell type in ACP PDOs, with a minor presence of KE, whereas the proportion of PE was significantly reduced or undetectable (*P* < 0.001) (Figure S3A–F, Supporting Information). This phenomenon may be attributed to the current cultivation conditions being more favorable for the proliferation of WE compared to PE.

To further explore whether ACP PDOs replicate the characteristic secretome of primary ACP, we conducted a re‐analysis of our bulk RNA‐sequencing data from ACP PDOs alongside existing bulk RNA‐sequencing data from ACP tissues (GSE94349). Additionally, we assessed the mRNA expression levels of *CTNNB1*, *LEF1*, *TCF1*, *AXIN2*, *SHH*, *PTCH1*, *TGFB1*, *BMP2*, *BMP4*, and *FGF3* in nine cases of ACP PDOs and their corresponding parental tissues using qPCR. These analyses collectively confirmed that ACP PDOs and primary ACP tissues exhibit comparable transcriptional levels of key secreted factors implicated in ACP pathogenesis, including members of the FGF (e.g., FGF3, FGF4), BMP (e.g., BMP4, BMP7), and TGF‐β (e.g., TGFB1, TGFB2) families (*P* > 0.05) (Figure S4A–L, Supporting Information). Furthermore, the expression levels of the WNT (*LEF1*, *TCF1*, *AXIN2*) and SHH (*PTCH1*) signaling pathways in ACP PDOs were found to be similar to those in primary ACP tissues (*P* > 0.05) (Figure S4A–L, Supporting Information). Consequently, these findings suggest that ACP PDOs accurately reflect the secretory phenotypic characteristics of the WE in primary ACP.

These findings provide compelling evidence that PDOs effectively preserve the histological architecture, morphological, and functional diversity of primary ACP tumors, with the WE identified as the predominant cell type within ACP PDOs.

### 2.4 PDO Models of ACP Retain the Genetic Characteristics of Original Tumor Tissues

**Figure 3:**
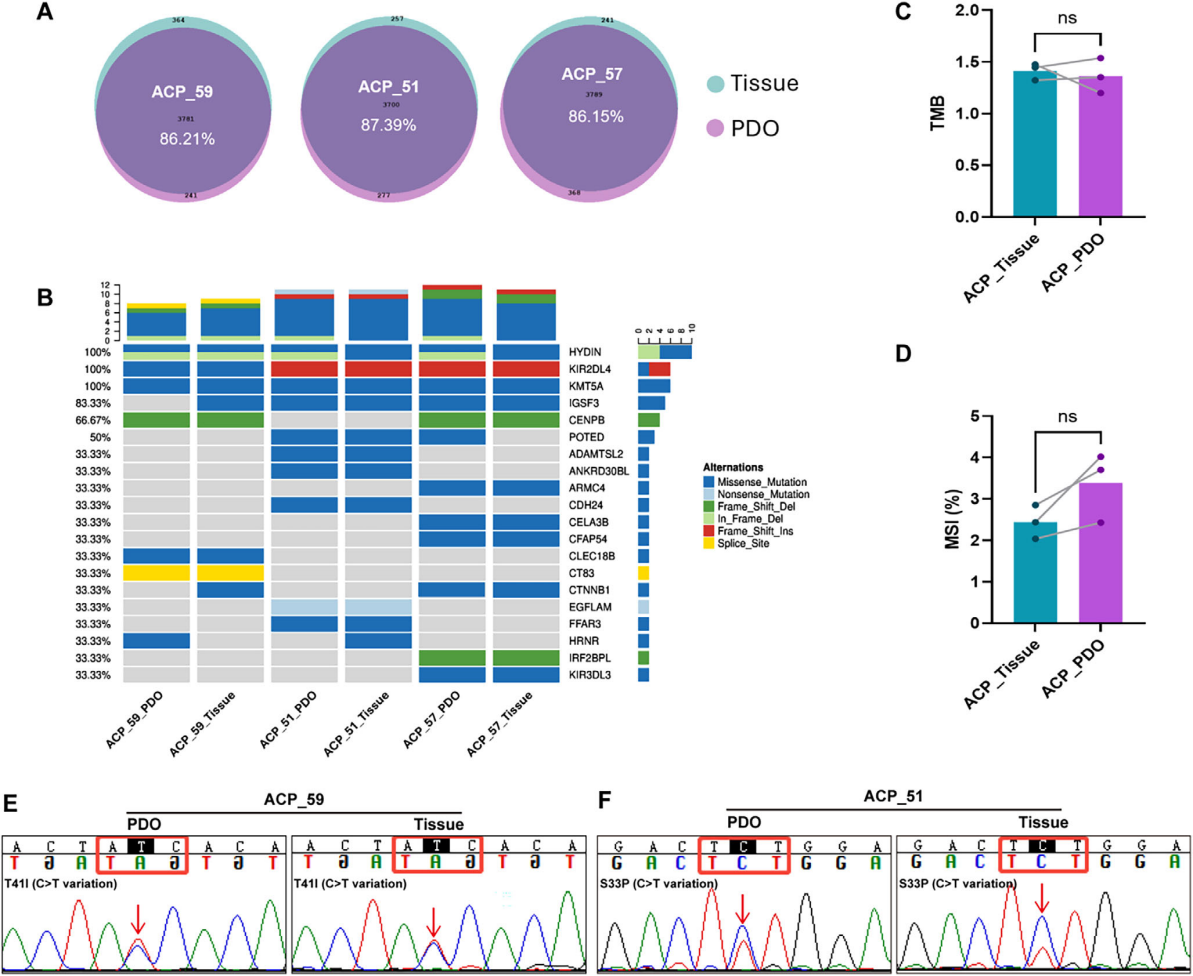
Whole‐exome sequencing (WES) reveals the consistency between ACP PDOs and corresponding parental tumors. A) Venn diagram of the accordance of ACP tumors and PDOs in patients 51, 57, and 59. B) Heatmap of the key somatic mutations of ACP tumors and PDOs in patients 51, 57, and 59. C) The tumor mutational burden (TMB) of ACP tumors and PDOs in patients 51, 57, and 59 (*n* = 3/group). D) The microsatellite instability (MSI) of ACP tumors and PDOs in patients 51, 57, and 59 (*n* = 3/group). E) Sanger sequencing chromatogram showing a typical point mutation in the *CTNNB1* gene (Thr41Ile) in PDOs and parental tumor tissue from patient ACP_59. F) Sanger sequencing chromatogram showing a typical point mutation in the *CTNNB1* gene (Ser33Phe) in PDOs and parental tumor tissue from patient ACP_51. In all graphs, data are presented as mean ± standard error of mean (SEM). Data between two groups are compared by an independent‐sample two‐tailed Student's *t*‐test for (C,D). *p < 0.05, **p < 0.01, ***p < 0.001, ns: not significant.

Previous studies have shown that tumor‐derived organoids accurately replicate the genomic characteristics of their corresponding tumors.^[^ ^28^, ^29^, ^30^, ^31^, ^32^, ^33^, ^34^ ^]^ To verify the genomic fidelity of ACP PDOs, we conducted whole‐exome sequencing (WES) on three matched sets of PDOs and primary tumor tissue samples from patients 51, 57, and 59. The analysis revealed that the PDOs preserved the majority of mutations found in the original tumor tissues, with an average concordance rate exceeding 85% across all three patient samples (**Figure** **3**A). Additionally, the mutation spectra of the tumors and their corresponding PDOs were highly similar (Figure S5A–C, Supporting Information), further corroborating the genetic fidelity of the PDO models. In relation to somatic mutations, all three patient samples demonstrated high‐frequency mutations in both tumor tissues and PDOs, aligning with typical ACP mutation profiles (Figure 3B). The tumor mutational burden (TMB) was also comparable across this exploratory cohort of three pairs of tumor tissues and PDOs, suggesting a faithful preservation of the genetic landscape of the original tumors within the PDOs (*P* > 0.05) (Figure 3C). Although microsatellite instability (MSI) was marginally elevated in PDOs compared to tumor tissues, this difference did not reach statistical significance (*P* > 0.05) (Figure 3D).

In a critical validation of our findings, we confirmed the presence of the hallmark *CTNNB1* mutation. Specifically, WES verified the identical activating mutation in *CTNNB1* in both the primary tumor and the PDOs from Patient 57 (Figure 3B). In contrast, for Patient 59, WES did not detect the specific low variant allele frequency (VAF) *CTNNB1* mutation (Thr41Ile) present in the primary tumor within the corresponding PDO. This absence is likely due to clonal selection during culture or the technical limitations of WES coverage depth for low‐frequency variants. However, WES did identify an alternative nonsense somatic *CTNNB1* variant within the same organoid line. Sanger sequencing corroborated the presence of the point mutation in exon 3 of *CTNNB1* (Thr41Ile) in the parental tissue, which was also retained in the PDOs (Figure 3E). Although WES did not identify any *CTNNB1* mutations in the PDOs and parental tissues of ACP_51, Sanger sequencing confirmed the presence of consistent *CTNNB1* mutations (Ser33Phe) in both the PDOs and parental tissues (Figure 3B and F). This finding highlights that while specific subclones may undergo evolutionary changes, the core genetic driver pathway remains conserved. The high degree of genetic fidelity, particularly concerning key driver pathways, affirms that our PDOs serve as robust models of the original tumors. In patient 51, *DMD* was the only driver mutation shared between the tumor and PDOs, whereas in patient 57, *SH3PXD2A* was the sole common mutation. Interestingly, neither of these genes has been previously associated with ACP, thereby adding layer of complexity to our understanding of the molecular mechanisms driving the disease. The absence of commonly recognized tumor driver genes in ACP further complicates the investigation of tumorigenesis and the development of therapeutic strategies. Despite these challenges, PDOs have demonstrated the ability to preserve key genetic characteristics of the original tumors, indicating their potential as a robust in vitro model for detailed study of ACP progression and treatment. These findings highlight the value of PDOs in capturing the genetic heterogeneity of ACP, positioning them as a promising tool for advancing our understanding of this rare and complex tumor.

### 2.5 Drug Response of PDO Models of ACP

**Figure 4:**
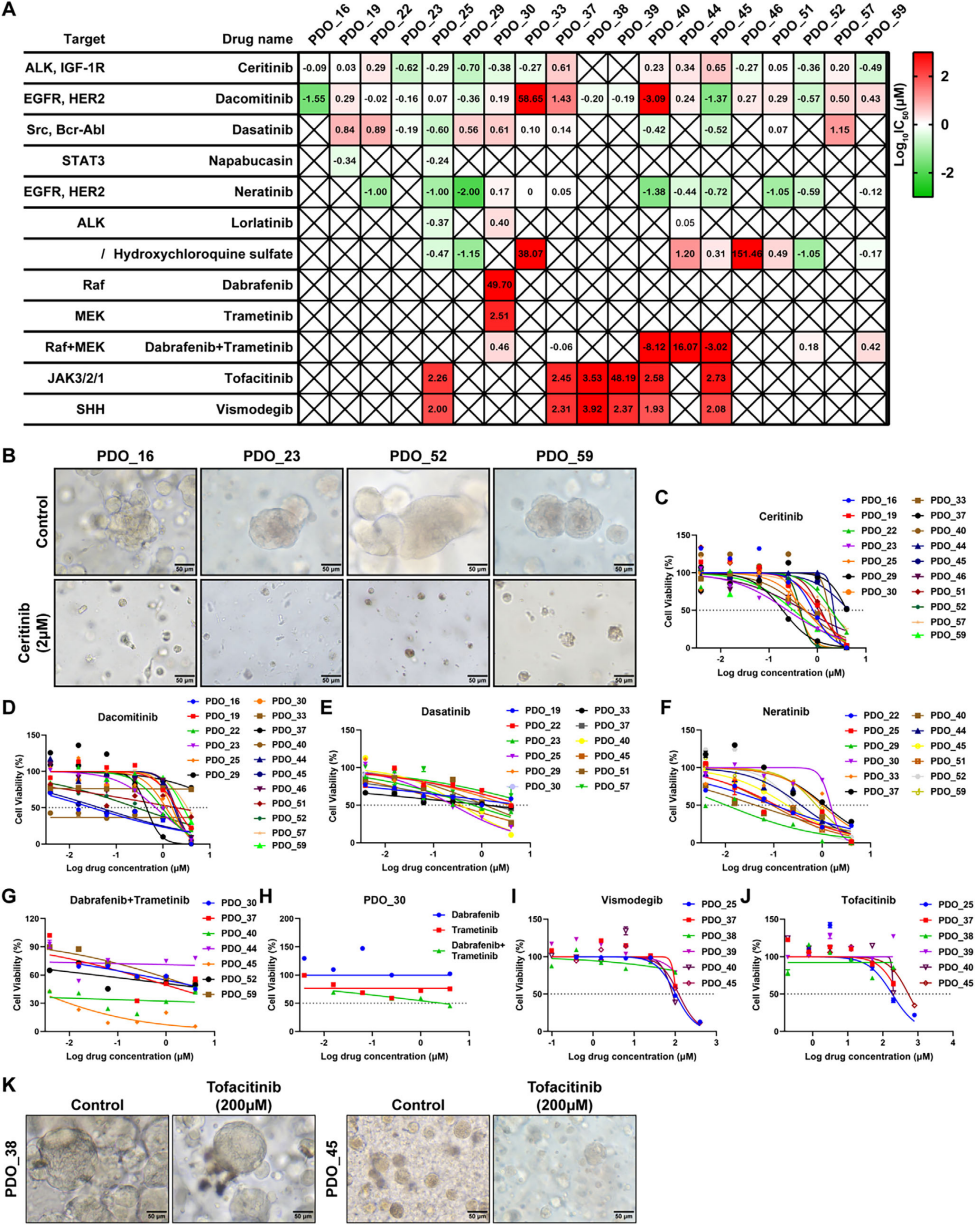
Drug response of ACP organoid models. A) Heatmap of log_10_IC_50_ values calculated from drug response analyses of PDO models derived from ACP patients by applying nonlinear regression (curve fitting). The putative drug targets of the tested compounds are listed on the left. B) Representative bright‐field images of Ceritinib‐treated ACP PDOs. C–J) Representative dose‐response curves for Ceritinib C), Dacomitinib D), Dasatinib E), Neratinib F), Dabrafenib combined with or without Trametinib G and H), Vismodegib I), and Tofacitinib J) treated with the indicated ACP PDO models. K) Representative bright‐field images of Tofacitinib‐treated ACP PDOs. In all graphs, data are presented as mean ± SEM. ALK, anaplastic lymphoma kinase; IGF‐1R, insulin‐like growth factor 1 receptor; EGFR, epidermal growth factor receptor; HER2, human epidermal growth factor receptor 2; STAT3, signal transducer and activator of transcription 3; MEK, mitogen‐activated protein kinase kinase; JAK, janus kinase; SHH, sonic hedgehog.

To investigate the potential application of ACP PDOs as preclinical models for evaluating drug responses and identifying therapeutic vulnerabilities, we conducted in vitro drug sensitivity assays using early passage ACP PDOs (passages 2–3). Notably, Sanger sequencing revealed that all PDOs harbor point mutations in the *CTNNB1* gene, thereby verifying the authenticity of the organoid origin (**Table** **2**; Figure S6A‐P, Supporting Information). We curated a panel of drugs based on their clinical relevance in cancer treatment, including investigational agents undergoing clinical trials, as well as their capacity to target specific signaling pathways or molecules of interest. The selected pharmacological agents comprised Ceritinib, which targets anaplastic lymphoma kinase (ALK) and insulin‐like growth factor 1 receptor (IGF‐1R); Dacomitinib and Neratinib, which target EGFR and human epidermal growth factor receptor 2 (HER2); Dasatinib, which targets Src/Bcr‐Abl; Napabucasin, which targets signal transducer and activator of transcription 3 (STAT3); Lorlatinib, which targets ALK; Dabrafenib, which targets Raf; Trametinib, which targets mitogen‐activated protein kinase kinase (MEK); Tofacitinib, which targets janus kinase 3/2/1 (JAK3/2/1); and Vismodegib, which targets the sonic hedgehog (SHH) pathway (**Figure** **4**A).

**Table 2:** Summary of *CTNNB1* Mutation Analysis of Patient‐derived ACP Organoids and Corresponding Parental Tumors.

Organoid No.	Patient No.	*CTNNB1* mutation			
		Nucleotide change		Effect of mutation	
		Primary tissue	PDOs	Primary tissue	PDOs
PDO_10	ACP_10	GGA>CGA (c.100G>C)	GGA>CGA (c.100G>C)	p.Gly34Arg	p.Gly34Arg
PDO_13	ACP_13	No mutation	TCT>TAT (c.98C>A)	No mutation	p.Ser33Try
PDO_14	ACP_14	GAC>AAC (c.94G>T)	GAC>AAC (c.94G>T)	p.Asp32Tyr	p.Asp32Tyr
PDO_16	ACP_16	GAC>AAC (c.94G>A)	GAC>AAC (c.94G>A)	p.Asp32Ala	p.Asp32Ala
PDO_18	ACP_18	Not detected	GCT>TCT (c.97G>T)	Not detected	p.Ser33Ala
PDO_19	ACP_19	ACC>GCC (c.121A>G)	ACC>GCC (c.121A>G)	p.Thr41Ala	p.Thr41Ala
PDO_22	ACP_22	GAC>GTC (c.95A>T)	GAC>GTC (c.95A>T)	p.Asp32Val	p.Asp32Val
PDO_23	ACP_23	ACC>ATC (c.122C>T)	ACC>ATC (c.122C>T)	p.Thr41Ile	p.Thr41Ile
PDO_25	ACP_25	ACC>ATC (c.122C>T)	ACC>ATC (c.122C>T)	p.Thr41Ile	p.Thr41Ile
PDO_27	ACP_27	Not detected	TCT>GCT (c.109T>G)	Not detected	p.Ser37Ala
PDO_29	ACP_29	TCT>TTT (c.98C>T)	TCT>TTT (c.98C>T)	p.Ser33Phe	p.Ser33Phe
PDO_30	ACP_30	GAC>CAC (c.94G>C)	GAC>CAC (c.94G>C)	p.Asp32His	p.Asp32His
PDO_33	ACP_33	GAC>AAC (c.94G>A)	GAC>AAC (c.94G>A)	p.Asp32Asn	p.Asp32Asn
PDO_37	ACP_37	ACC>ATC (c.122C>T)	ACC>ATC (c.122C>T)	p.Thr41Ile	p.Thr41Ile
PDO_38	ACP_38	TCT>TTT (c.110C>T)	TCT>TTT (c.110C>T)	p.Ser37Phe	p.Ser37Phe
PDO_39	ACP_39	ACC>ATC (c.122C>T)	ACC>ATC (c.122C>T)	p.Thr41Ile	p.Thr41Ile
PDO_40	ACP_40	ATC>AGC (c.104T>G)	ATC>AGC (c.104T>G)	p.Ile35Ser	p.Ile35Ser
PDO_44	ACP_44	GGA>GAA (c.101G>A)	GGA>GAA (c.101G>A)	p.Gly34Glu	p.Gly34Glu
PDO_45	ACP_45	TCT>TTT (c.110C>T)	TCT>TTT (c.110C>T)	p.Ser37Phe	p.Ser37Phe
PDO_46	ACP_46	No mutation	TCT>TTT (c.134C>T)	No mutation	p.Ser45Phe
PDO_51	ACP_51	TCT>TTT (c.98C>T)	TCT>TTT (c.98C>T)	p.Ser33Phe	p.Ser33Phe
PDO_52	ACP_52	No mutation	GGA>AGA (c.100G>A)	No mutation	p.Gly34Arg
PDO_59	ACP_59	ACC>ATC (c.122C>T)	ACC>ATC (c.122C>T)	p.Thr41Ile	p.Thr41Ile

Drug sensitivity assays were conducted on 19 ACP PDO lines, with drug sensitivity quantified using IC_50_ values. Due to inherent variations in growth kinetics and material availability across PDO lines, a complete matrix of all drugs tested on every line was not feasible. Dose‐response curves were employed to depict the reactions of the 19 PDOs to the 11 drugs. The findings revealed a heterogeneous response profile of the ACP PDOs to the drug panel (Figure 4A; Figure S7A–Q, Supporting Information). Notably, a pronounced sensitivity to the IGF‐1R inhibitor Ceritinib was consistently observed across all 17 tested PDO lines, with IC_50_ values ranging from 0.20 µm in PDO_29 to 4.50 µm in PDO_45 (Figure 4B,C). Conversely, the response to the EGFR inhibitor Dacomitinib exhibited greater variability. Eighteen out of nineteen ACP PDO lines demonstrated sensitivity to Dacomitinib, with IC_50_ values ranging from 0.0008 µm in PDO_40 to 26.91 µm in PDO_37. However, PDO_33 displayed marked resistance to Dacomitinib, with an IC_50_ exceeding 100 µm (Figure 4D). Sensitivity to Dasatinib was observed in PDO_19, PDO_22, PDO_23, PDO_25, PDO_29, PDO_30, PDO_33, PDO_37, PDO_40, PDO_45, PDO_51, and PDO_57, with IC_50_ values ranging from 0.25 µm in PDO_25 to 14.27 µm in PDO_57 (Figure 4E). Furthermore, PDO_22, PDO_25, PDO_29, PDO_30, PDO_33, PDO_37, PDO_40, PDO_44, PDO_45, PDO_51, PDO_52, and PDO_59 exhibited sensitivity to Neratinib, with IC_50_ values spanning from 0.01 µm in PDO_29 to 1.48 µm in PDO_30 (Figure 4F). Finally, PDO_30, PDO_37, PDO_40, PDO_45, PDO_52, and PDO_59 were sensitive to the combination therapy of Dabrafenib and Trametinib, whereas PDO_44 was resistant to this treatment regimen (Figure 4G). Interestingly, our investigation revealed that the individual administration of Dabrafenib or Trametinib to PDO_30 resulted in IC_50_ values exceeding 100 µm. However, when these agents were combined, the IC_50_ value decreased significantly to 2.87 µm (Figure 4H), suggesting a potential synergistic interaction between Dabrafenib and Trametinib. The precise mechanisms underlying this synergy warrant further exploration. Additionally, the organoid drug sensitivity assays demonstrated that PDOs exhibited relatively low sensitivity to Vismodegib and Tofacitinib, as evidenced by their comparatively higher IC_50_ values (Figure 4I–K). These findings suggest that ACP PDOs respond variably to the pharmacological agents evaluated in this study and underscore their utility as a robust model for drug screening.

### 2.6 Ceritinib Inhibits the Growth of PDO Models of ACP by Targeting IGF‐1R

**Figure 5:**
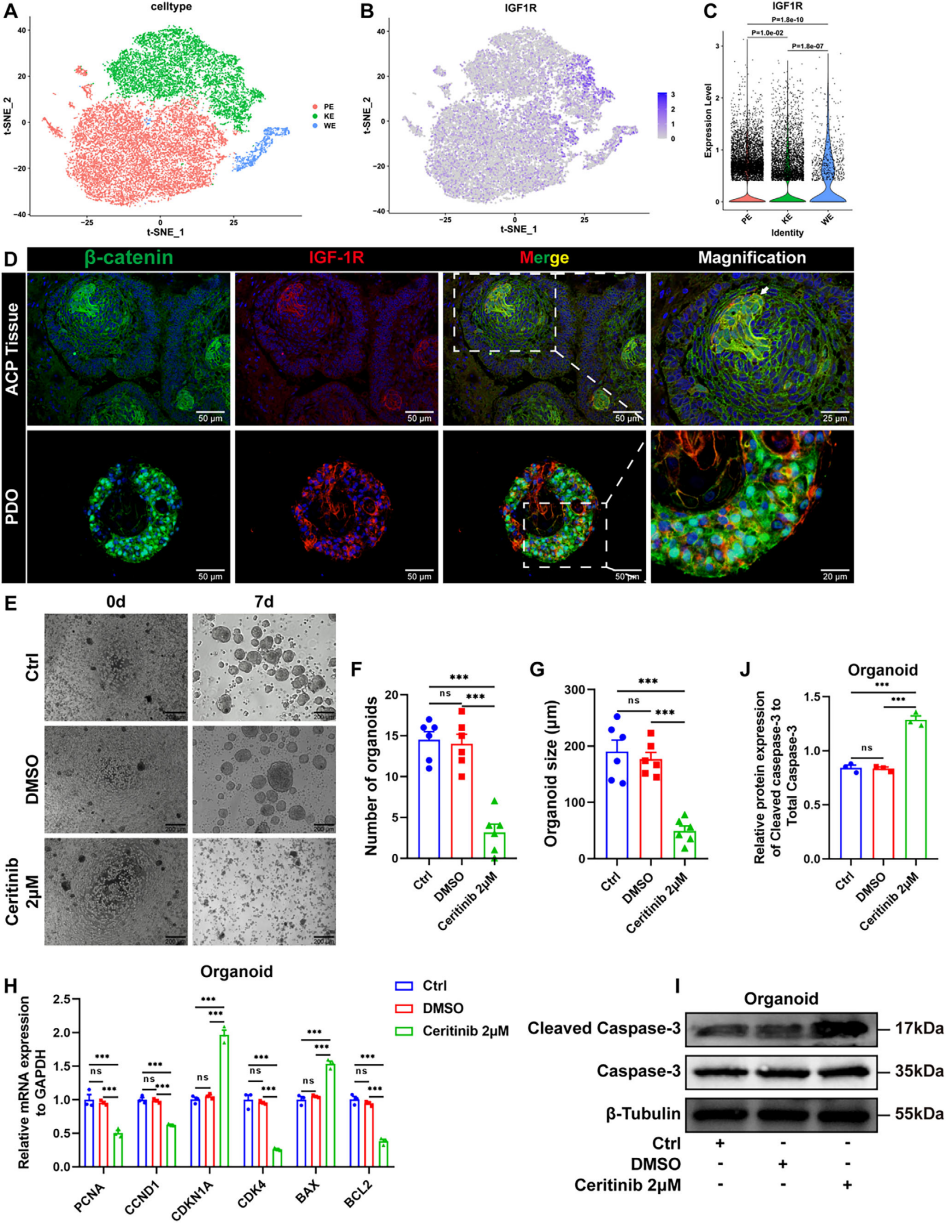
Inhibition of ACP PDO Growth by Ceritinib. A) t‐distributed stochastic neighbor embedding (t‐SNE) clustering plot of publicly available single‐cell RNA sequencing data of ACP showing the distribution of tumor cell clusters based on marker gene expression. B,C) t‐SNE clustering plot B) and violin plot C) highlighting the expression patterns of the insulin‐like growth factor 1 receptor (IGF‐1R) across three distinct cell types. D) Dual immunofluorescence staining of β‐catenin and IGF‐1R of ACP tissue samples (upper panel) and PDOs (lower panel), representative images are shown. Boxed area is enlarged and presented on the right. E) ACP PDOs were treated with or without 2 µm Ceritinib, representative reflection microscopy images of 0 day and 7 days are shown. F,G) Quantification of ACP PDOs as described in (E); F) number, and G) diameter (*n* = 6/group). H) Relative mRNA expression levels of *PCNA*, *CCND1*, *CDKN1A*, *CDK4*, *BAX*, and *BCL2* in ACP PDOs treated with or without 2 µm Ceritinib (*n* = 3/group). I,J) The protein expression levels of Cleaved Caspase‐3 in ACP PDOs treated with or without 2 µm Ceritinib were analyzed using Western blotting, and the results were represented as the expression of Cleaved Caspase‐3 normalized to that of total Caspase‐3 (*n* = 3/group). In all graphs, data are presented as mean ± SEM. Data among three groups are compared by an one‐way ANOVA test followed by a Tukey post hoc test for (C), F), G), and J), and compared by a two‐way ANOVA test followed by a Tukey post hoc test for (H). *p < 0.05, ^**^ *p *< 0.01, ^***^ *p *< 0.001, ns: not significant. DMSO, dimethylsulfoxide; PI, propidium iodide; FITC, fluorescein isothiocyanate.

Drug sensitivity assays revealed that 17 ACP PDO lines were sensitive to Ceritinib, as evidenced by relatively low IC_50_ values and high inhibition rates, thereby underscoring Ceritinib's efficacy in inhibiting the proliferation of ACP PDOs (Figure 4A,B). Considering that Ceritinib targets IGF‐1R and ALK, we initially examined the expression of IGF‐1R and ALK in ACP tissues through scRNA sequencing data^[^ ^36^ ^]^ analysis. The findings demonstrated positive expression of IGF‐1R in ACP cells (**Figure** **5**A,B; Figure S8A, Supporting Information). Further analysis indicated that, relative to KE and PE, IGF‐1R expression was elevated in WE, aligning with observations from immunofluorescence staining (*P* < 0.001) (Figure 5C,D). Conversely, the expression level of ALK in ACP cells was significantly lower than that of IGF‐1R (*P* < 0.001) (Figure S8B–D, Supporting Information). To validate the aforementioned single‐cell data analysis results, we employed quantitative PCR (qPCR) and immunohistochemical staining to assess the expression levels of IGF‐1R and ALK in ACP tissues and ACP PDOs. The findings indicated that the expression level of IGF‐1R was significantly elevated compared to that of ALK (*P* < 0.001) (Figure S8E–H, Supporting Information).

To further elucidate that Ceritinib predominantly inhibits the proliferation of ACP PDOs via IGF‐1R, we utilized small interfering RNAs (siRNAs) to specifically target and downregulate IGF‐1R expression in ACP PDOs. Western blot analysis revealed that siIGF‐1R#2 exhibited the most pronounced knockdown effect (*P* < 0.001) (Figure S8I,J, Supporting Information). Subsequently, we observed that IGF‐1R knockdown significantly suppressed the growth of ACP PDOs, induced apoptosis, and inhibited AKT phosphorylation, mirroring the effects of Ceritinib (Figure S8K,L, Supporting Information). Significantly, the addition of Ceritinib to PDOs with knocked‐down IGF‐1R did not yield additional effects (Figure S8K,L, Supporting Information), suggesting that the efficacy of Ceritinib is contingent upon the presence of IGF‐1R. Furthermore, our findings demonstrated that treatment of ACP PDOs with recombinant human IGF‐1 enhanced the phosphorylation of PI3K and AKT. However, pretreatment with Ceritinib effectively inhibited the IGF‐1‐induced activation of the PI3K/AKT pathway (Figure S8M, Supporting Information), indicating that Ceritinib acts as an antagonist to the IGF‐1/IGF‐1R signaling pathway in ACP PDOs. Collectively, these results underscore that IGF‐1R is the primary target of Ceritinib in ACP PDOs.

In the subsequent phase of our study, we examined the impact of Ceritinib on the established ACP cell line, STAM4.^[^ ^37^ ^]^ To verify the mutation status of *CTNNB1* in STAM4 cells, Sanger sequencing was employed, revealing a point mutation (Thr41Ile) in exon 3 of *CTNNB1* (Figure S9A, Supporting Information). The CCK‐8 assay demonstrated that Ceritinib significantly inhibited the proliferation of STAM4 cells in comparison to both control and vehicle (DMSO) groups (*P* < 0.001) (Figure S9B, Supporting Information). To ascertain whether this inhibition of proliferation was attributable to changes in the cell‐cycle profile, we conducted flow cytometry analysis. The findings indicated that Ceritinib induced cell cycle arrest at the S phase in STAM4 cells (Figure S9C,D, Supporting Information). Additionally, Ceritinib significantly promoted apoptosis in STAM4 cells relative to controls, as determined by flow cytometry (*P* < 0.001) (Figure S9E,F, Supporting Information). The effects of Ceritinib were further assessed in vitro on ACP PDOs. Treatment with Ceritinib substantially decreased both the number and size of ACP PDOs compared to control groups (*P* < 0.001) (Figure 5E–G). In alignment with these findings, the mRNA expression levels of genes associated with proliferation and the cell cycle, such as *PCNA*, *CCND1*, and *CDK4*, were markedly downregulated in Ceritinib‐treated STAM4 cells and ACP PDOs, whereas *CDKN1A* expression was significantly upregulated, as determined by qPCR (*P* < 0.001) (Figure 5H; Figure S9G, Supporting Information). Additionally, the expression of the anti‐apoptotic factor *BCL2* was substantially reduced, while the expression of pro‐apoptotic factors *BAX* and cleaved caspase‐3 was significantly elevated in response to Ceritinib treatment in both STAM4 cells and ACP PDOs, as analyzed by qPCR and Western blotting, respectively (*P* < 0.05) (Figure 5H–J; Figure S9G‐I, Supporting Information). Collectively, these results indicate that Ceritinib induces cell cycle arrest and apoptosis in ACP PDOs by targeting the IGF‐1R.

### 2.7 The PI3K/AKT/GSK3β/β‐Catenin Axis is Downregulated in Ceritinib‐Treated PDO Models of ACP

**Figure 6:**
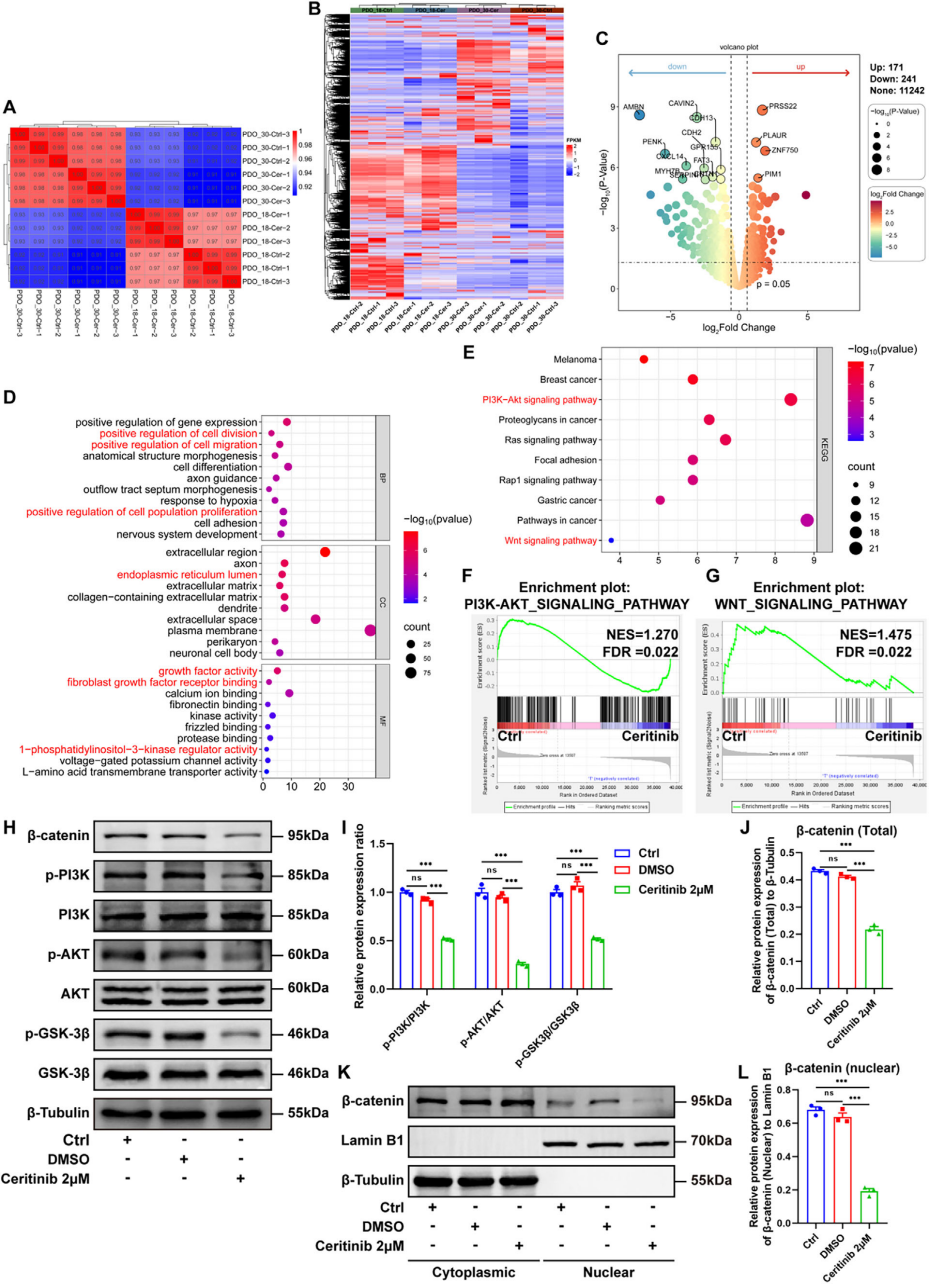
PI3K/AKT/GSK‐3β/β‐catenin pathway is downregulated in Ceritinib‐treated ACP PDOs. A) Correlation of gene expression between Ceritinib (Cer)‐treated ACP PDOs and control (Ctrl) ACP PDOs. B) Gene expression profiling via RNA‐sequencing shows differentially expressed genes (DEGs) between Cer‐treated ACP PDOs and Ctrl‐ACP PDOs based on hierarchical clustering with the following filter criteria: a false discovery rate (FDR) < 0.05 and a fold change > 1.5. C) A volcano plot of *p*‐values as a function of weighted fold change showing the down‐regulated and up‐regulated DEGs between the two groups. D) Gene Ontology (GO) functional clustering analysis of the down‐regulated DEGs of two groups as indicated. E) Kyoto Encyclopedia of Genes and Genomes (KEGG) functional clustering analysis of the down‐regulated DEGs of two groups as indicated. F,G) Gene set enrichment analysis (GSEA) of the PI3K‐AKT signaling pathway F) and the WNT signaling pathway G) between Cer‐treated ACP PDOs and Ctrl‐ACP PDOs. H,I) The phosphorylation levels of PI3K, AKT, and GSK‐3β in ACP PDOs treated with or without 2 µM Ceritinib were analysed by Western blot, and the results were represented as the expression of phosphorylated protein normalized to the expression of total protein, respectively (*n* = 3/group). J) The protein expression of total β‐catenin in ACP PDOs treated with or without 2 µm Ceritinib was analysed by Western blot, and the results were represented as the expression of total β‐catenin normalized to the expression of β‐Tubulin (*n* = 3/group). K,L) The protein expression of nuclear β‐catenin in ACP PDOs treated with or without 2 µm Ceritinib was analysed by Western blot, and the results were represented as the expression of nuclear β‐catenin normalized to the expression of Lamin B1 (*n* = 3/group). In all graphs, data are presented as mean ± SEM. Data among three groups are compared by an one‐way ANOVA test followed by a Tukey post hoc test for (I) and (L), and compared by a two‐way ANOVA test followed by a Tukey post hoc test for (J). *p < 0.05, **p < 0.01, ***p < 0.001, ns: not significant. NES, normalized enrichment score; BP, biological processes; CC, cellular components; MF, molecular functions.

To further explore the alterations in gene expression induced by Ceritinib and to elucidate the underlying mechanisms, we conducted RNA transcriptomic sequencing analysis on ACP PDOs from both Ceritinib‐treated and control groups. Gene expression within each group of biological replicates exhibited high correlation, with correlation coefficients equal to or greater than 0.99 (**Figure** **6**A). In contrast, comparisons between the groups revealed correlation coefficients ranging from 0.91 to 0.97 between the Ceritinib‐treated and control ACP PDOs (Figure 6A). A hierarchical clustering heatmap further illustrated significant differences in gene expression between the Ceritinib‐treated and control groups (Figure 6B). Differential gene expression analysis identified a total of 412 differentially expressed genes (DEGs). In comparison to the control group, 171 genes were upregulated and 241 genes were downregulated in the Ceritinib‐treated group (Figure 6C). The Gene Ontology (GO) analysis demonstrated that the downregulated DEGs were significantly enriched in biological processes (BP), cellular components (CC), and molecular functions (MF) predominantly associated with the positive regulation of cell division, cell migration, and cell population proliferation, as well as growth factor activity, fibroblast growth factor receptor binding, and 1‐phosphatidylinositol‐3‐kinase regulator activity (Figure 6D). Furthermore, the results of the Kyoto Encyclopedia of Genes and Genomes (KEGG) pathway analysis are presented in Figure 6E, elucidating the interactions of signaling pathways between the Ceritinib‐treated and control groups. These findings highlight the pivotal role of Ceritinib in modulating the PI3K/Akt and Wnt signaling pathways (Figure 6E). Gene Set Enrichment Analysis (GSEA) is employed as a powerful analytical tool to explore the complex relationship between gene functions and their expressions. Our results confirmed a significant downregulation of both the PI3K/Akt and Wnt signaling pathways in the Ceritinib‐treated group compared to controls, indicating a substantial inhibition of these two signaling pathways by Ceritinib (Figure 6F,G).

To corroborate the findings from RNA sequencing, we investigated the impact of Ceritinib on the PI3K/AKT/Wnt signaling pathway through Western blotting and qPCR. The Western blot results demonstrated that Ceritinib significantly reduced the phosphorylation ratios of p‐PI3K/PI3K, p‐AKT/AKT, and p‐GSK3β/GSK3β in comparison to the controls (*P *< 0.001) (Figure 6H,I). Additionally, Ceritinib markedly diminished both the overall levels and the nuclear translocation of β‐catenin, a downstream target of AKT (*P* < 0.001) (Figure 6H,J–L). qPCR analysis further corroborated these findings by showing a significant reduction in the mRNA expression levels of *PIK3CA*, *AKT1*, and *CTNNB1* in ACP PDOs treated with Ceritinib compared to controls (*P* < 0.05) (Figure S10A–C, Supporting Information). Collectively, these results suggest that Ceritinib effectively inhibits the PI3K/AKT/GSK‐3β/β‐catenin signaling axis at both post‐transcriptional phosphorylation and transcriptional levels.

**Figure 7:**
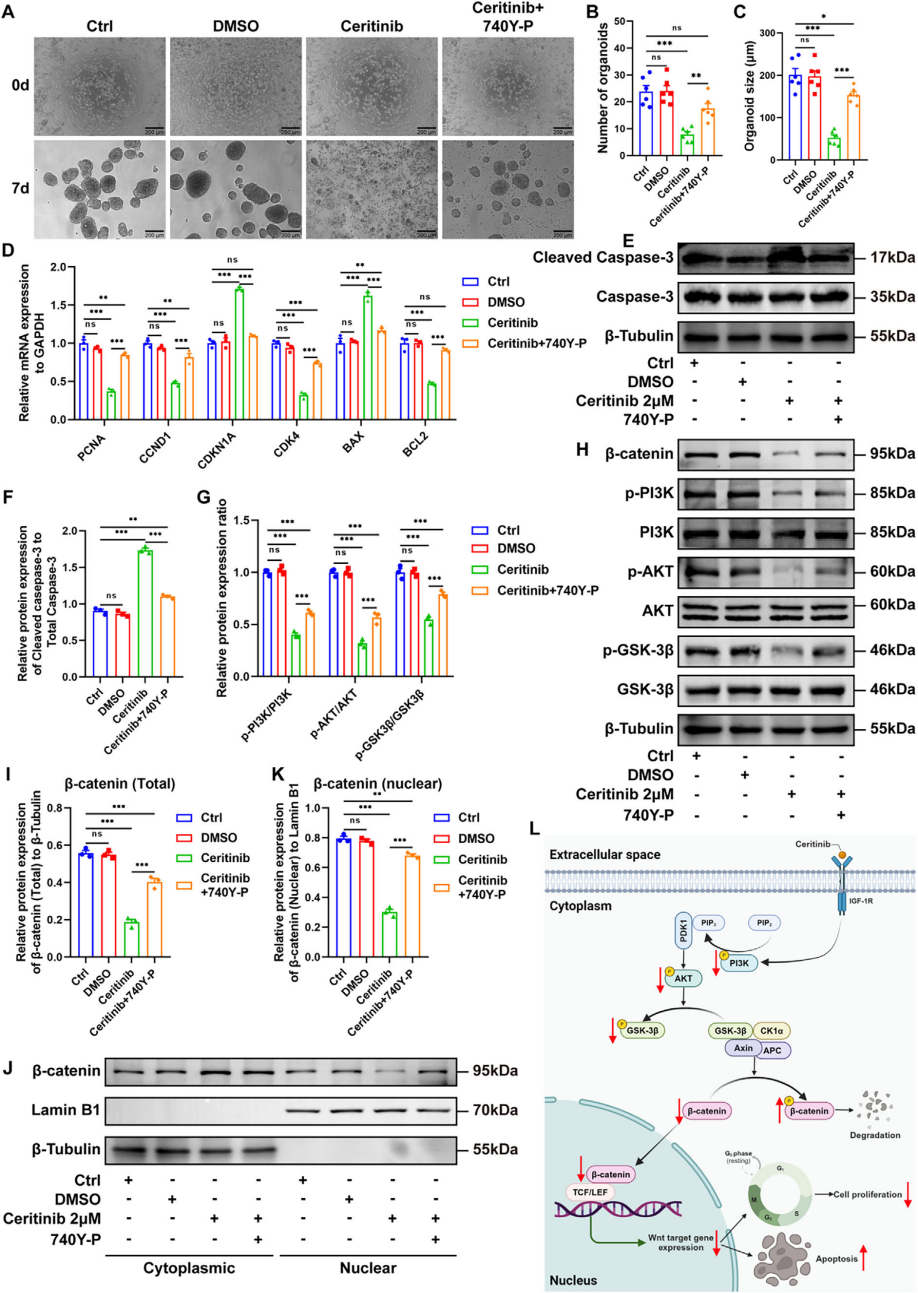
Ceritinib inhibits the growth of ACP PDOs by downregulating PI3K/AKT/GSK‐3β/β‐catenin pathway. A) ACP PDOs were treated with either 2 µm Ceritinib, 2 µm Ceritinib combined with 30 µg mL^−1^ 740Y‐P, or untreated controls, representative reflection microscopy images of 0 day and 7 days are shown. B, C) Quantification of ACP PDOs as described in (A); B) number, and C) diameter (*n* = 6/group). D) Relative mRNA expression levels of *PCNA*, *CCND1*, *CDKN1A*, *CDK4*, *BAX*, and *BCL2* in ACP PDOs treated with either 2 µm Ceritinib, 2 µm Ceritinib combined with 30 µg/ml 740Y‐P, or untreated controls (*n* = 3/group). E,F) The protein expression of Cleaved Caspase‐3 in ACP PDOs treated with either 2 µm Ceritinib, 2 µm Ceritinib combined with 30 µg/ml 740Y‐P, or untreated controls was analysed by Western blot, and the results were represented as the expression of Cleaved Caspase‐3 normalized to the expression of total Caspase‐3 (*n* = 3/group). G, H) The phosphorylation levels of PI3K, AKT, and GSK‐3β in ACP PDOs treated with either 2 µm Ceritinib, 2 µm Ceritinib combined with 30 µg mL^−1^ 740Y‐P, or untreated controls were analysed by Western blot, and the results were represented as the expression of phosphorylated protein normalized to the expression of total protein, respectively (*n* = 3/group). I) The protein expression of total β‐catenin in ACP PDOs treated with either 2 µm Ceritinib, 2 µm Ceritinib combined with 30 µg mL^−1^ 740Y‐P, or untreated controls was analysed by Western blot, and the results were represented as the expression of total β‐catenin normalized to the expression of β‐Tubulin (*n* = 3/group). J,K) The protein expression of nuclear β‐catenin in ACP PDOs treated with either 2 µm Ceritinib, 2 µm Ceritinib combined with 30 µg mL^−1^ 740Y‐P, or untreated controls was analysed by Western blot, and the results were represented as the expression of nuclear β‐catenin normalized to the expression of Lamin B1 (*n* = 3/group). L) A schematic representation of the signaling pathways involving Ceritinib‐mediated PI3K, AKT, GSK‐3β, β‐catenin, cell cycle arrest, and apoptosis in ACP PDOs (Created with BioRender.com). In all graphs, data are presented as mean ± SEM. Data among four groups are compared by an one‐way ANOVA test followed by a Tukey post hoc test for (B), (C), (F), (I), and (K), and compared by a two‐way ANOVA test followed by a Tukey post hoc test for (D) and (G). *p < 0.05, **p < 0.01, ***p < 0.001, ns: not significant.

To further elucidate the role of the PI3K/AKT/GSK‐3β/β‐catenin signaling pathway in mediating Ceritinib‐induced cell cycle arrest and apoptosis in ACP PDOs, we conducted experiments using ACP PDOs treated with Ceritinib both in the presence and absence of 740Y‐P, a known PI3K agonist. In vitro organoid culture assays demonstrated that 740Y‐P significantly mitigated the effects of Ceritinib on the reduction of both the number and size of ACP PDOs (*P* < 0.01) (**Figure** **7**A–C). Subsequently, we examined whether 740Y‐P could counteract the influence of Ceritinib on cell cycle and apoptosis‐related molecular markers in ACP PDOs. Treatment with 740Y‐P resulted in an attenuation of Ceritinib's effects, as evidenced by an increase in the mRNA expression levels of *PCNA*, *CCND1*, *CDK4*, and *BCL2*, alongside a decrease in the mRNA levels of *CDKN1A* and *BAX*, and a reduction in the protein levels of cleaved caspase‐3 (*P* < 0.001) (Figure 7D–F). Finally, the impact of 740Y‐P on the modulation of the PI3K/AKT/GSK‐3β/β‐catenin signaling pathway in ACP PDOs following Ceritinib treatment was evaluated using Western blotting and qPCR. Relative to the group treated solely with Ceritinib, 740Y‐P was observed to enhance the phosphorylation levels of PI3K, AKT, and GSK‐3β, as well as to mitigate the reduction in both total and nuclear β‐catenin levels induced by Ceritinib (*P* < 0.001) (Figure 7G–K). However, the mRNA expression levels of *PIK3CA*, *AKT1*, and *CTNNB1* in ACP PDOs co‐treated with Ceritinib and 740Y‐P did not significantly differ from those in ACP PDOs treated with Ceritinib alone (*P* > 0.05) (Figure S10D–F, Supporting Information). This suggests that 740Y‐P may not exert its regulatory effects on the PI3K/AKT/GSK‐3β/β‐catenin signaling axis at the transcriptional level.

Taken together, these findings indicate that Ceritinib induces cell cycle arrest and apoptosis in ACP PDOs through the downregulation of the PI3K/AKT/GSK‐3β/β‐catenin signaling axis (Figure 7L).

### 2.8 Pharmacotyping of ACP PDOs Reflects the Clinical Treatment Response of ACP Patients

**Figure 8:**
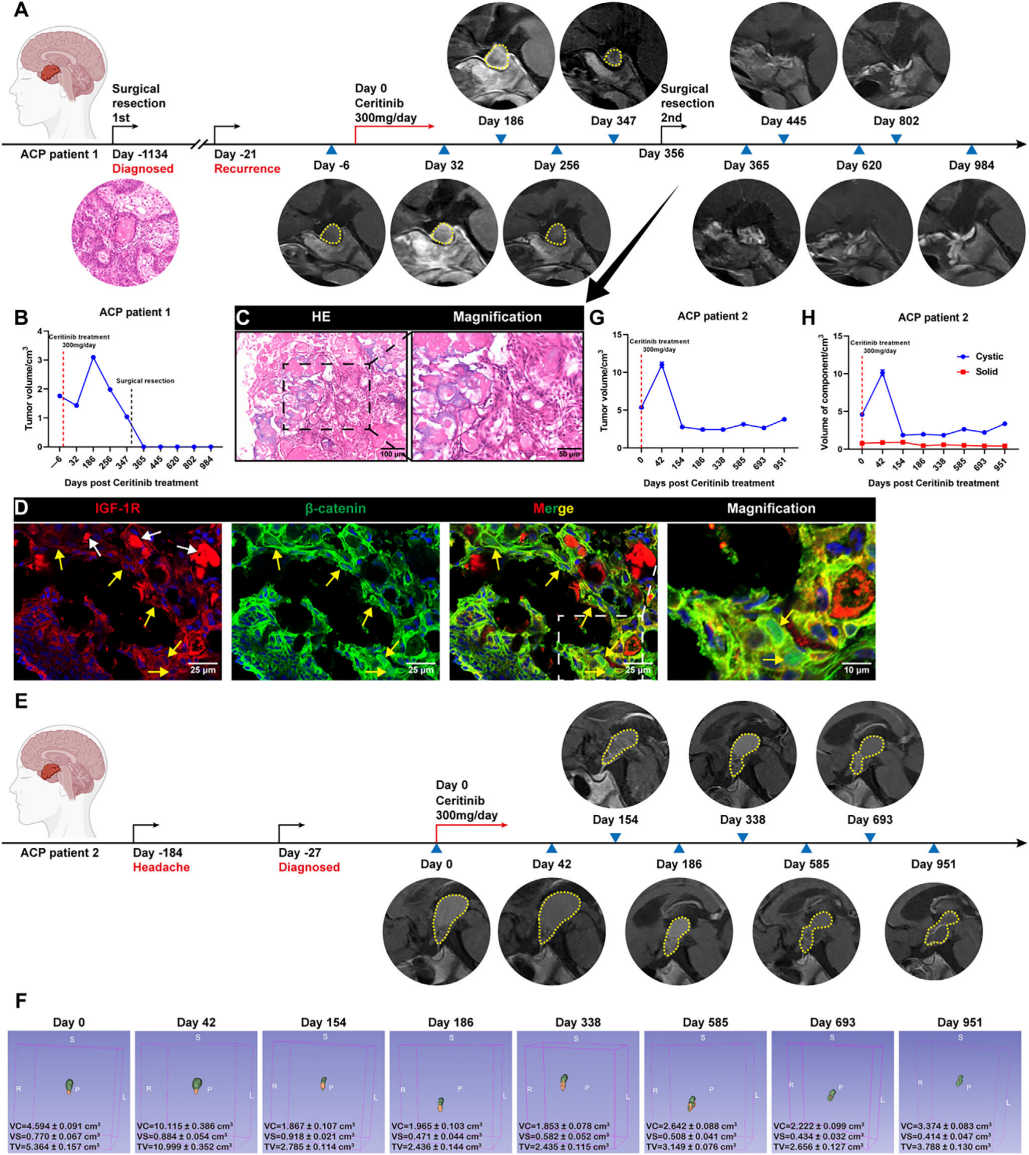
ACP PDOs reflect the clinical therapeutic responses of ACP patients. A) Timeline from the first presentation is indicated in days. The treatment procedure and the sequential contrast‐enhanced T1‐weighted (sagittal) magnetic resonance imaging (MRI) scans of ACP patient 1, spanning from initial diagnosis to the most recent follow‐up. Medical therapy with Ceritinib was started on Day 0. B) The temporal changes in tumor volume for patient 1 throughout the follow‐up period. C) Representative H&E staining of tissue samples obtained from patient 1 following the second surgical resection. Boxed area is enlarged and presented on the right. D) Dual immunofluorescence staining of β‐catenin and IGF‐1R in tissue samples from patient 1 post‐second surgical resection, representative images are shown. Yellow arrows indicate tumor cells exhibiting nuclear translocation of β‐catenin, while white arrows denote false positive staining due to wet keratin and calcification. Boxed area is enlarged and presented on the right. E) Timeline from the first presentation is indicated in days. The treatment procedure and the sequential contrast‐enhanced T1‐weighted (sagittal) MRI scans of ACP patient 2, from diagnosis to the last follow‐up. Medical therapy with Ceritinib was started on Day 0. F) The sequential 3D reconstruction images of the tumor of ACP patient 2 using the 3D Slicer software, from diagnosis to the last follow‐up. The green part indicates the cystic components within the tumor tissue, while the orange part denotes the solid components. G) The temporal changes in tumor volume for patient 2 over the follow‐up period based on 3D reconstruction performed by three senior radiologists (*n* = 3). H) The temporal changes in volume of the solid and cystic component of the tumor for patient 2 over the follow‐up period based on 3D reconstruction performed by three senior radiologists (*n* = 3). In all graphs, data are presented as mean ± SEM. VC, Volume of cystic component. VS, Volume of solid component. TV, Tumor volume.

To evaluate whether pharmacotyping of ACP PDOs accurately reflects patient drug responses, we analyzed two ACP cases with retrospective clinical data available. Patient 1 received a clinical diagnosis of ACP and underwent initial surgical resection in July 2019 (Day ‐1134, **Figure** **8**A). During routine follow‐up, tumor recurrence was identified in August 2022 (Day ‐21, Figure 8A). Magnetic resonance imaging (MRI) revealed that the tumor was adherent to critical peripheral structures, presenting a high risk for surgical intervention. Consequently, after obtaining informed consent from Patient 1 and their family, Ceritinib (300 mg once daily) was administered to reduce tumor size before a second surgical procedure. Regular assessments of the patient's liver and kidney functions indicated that the patient did not experience any intolerable side effects and demonstrated good tolerance to the treatment. Following Ceritinib administration, the tumor volume initially fluctuated but subsequently decreased progressively, reaching its minimum size (1.04 cm^3^) before the second surgery in August 2023 (Day 347, Figure 8A,B; Figure S11, Supporting Information). The postoperative pathological examination of the excised tumor tissue revealed significant fibrosis and collagenous fiber, abundant wet keratin and calcification, and sparsely distributed tumor cells (Figure 8C). Concurrently, we assessed the expression of IGF‐1R in the tumor tissue obtained from the second surgical resection of Patient 1 using immunofluorescence staining. The findings indicated positive expression of IGF‐1R in the residual tumor cells, providing a structural basis for the potential antitumor activity of Ceritinib (Figure 8D). Patient 2 presented with headache symptoms in February 2022 (Day ‐184) and was diagnosed with ACP via MRI in July 2022 (Day ‐27, Figure 8E). Given the large tumor size and hypothalamic involvement, there was a high anticipated risk of postoperative hypothalamic dysfunction. To reduce surgical risks, a targeted treatment regimen of Ceritinib at a dosage of 300 mg once daily was commenced following the acquisition of informed consent from both the patient and their family members. Subsequent imaging assessments, including 3D reconstruction, revealed an initial reactive increase in tumor volume at the onset of treatment, followed by a gradual reduction. By July 2023 (Day 338), the tumor volume had reached its minimum (2.435 ± 0.115 cm^3^) and subsequently demonstrated a trend toward stabilization (Figure 8E–G; Figures S12 and S13, Supporting Information). We further analysed the volume changes of the cystic and solid components within the tumor. The results showed that although the volume of the cystic component was fluctuating, the volume of the tumor's solid component had decreased to 53.77% of its pre‐treatment level at the last follow‐up (Figure 8H). While these two cases provide the first clinical context for our in vitro findings, the ambiguous radiological outcomes, particularly the initial tumor growth, are a significant concern. Therefore, these results should be interpreted with extreme caution and viewed strictly as a preliminary, hypothesis‐generating observation. They underscore the urgent need for well‐designed, prospective clinical trials to determine if Ceritinib has any true therapeutic role in ACP.

## 3 Discussion

In the present study, we successfully established a robust platform of patient‐derived ACP organoids and demonstrated its utility for drug screening, identifying Ceritinib as a promising therapeutic agent for ACP. Our objective was to establish patient‐derived ACP organoids to address the paucity of reliable in vitro models for this challenging disease. Ultimately, we successfully generated ACP PDOs using the current advanced organoid culture techniques with certain modifications. These PDOs faithfully maintained the morphological, histological, and molecular characteristics of human ACP over extended culture periods. In vitro drug sensitivity assays conducted with these PDOs revealed that the IGF‐1R inhibitor, Ceritinib, significantly inhibited the growth of ACP PDOs. Mechanistically, Ceritinib exerted its inhibitory effect primarily by downregulating the IGF‐1R/PI3K/AKT/GSK‐3β/β‐catenin signaling axis, suggesting the potential therapeutic efficacy of IGF‐1R inhibitors in ACP patients. Furthermore, preliminary clinical observations of Ceritinib treatment in two ACP patients suggest its potential anti‐tumor capabilities against ACP.

In cancer research, frequently utilized models encompass 2D‐cultured tumor cells, including primary tumor cells and immortalized cell lines, as well as animal models such as xenograft models. However, conventional culture techniques for human primary ACP cells often lead to limited lifespans and low success rates, in contrast to those observed in malignant tumors. To date, only one immortalized ACP cell line has been established,^[^ ^37^ ^]^ which significantly restricts the range of available models and consequently impedes the advancement of ACP research. Although ACP‐derived xenograft models^[^ ^38^ ^]^ and transgenic mouse models^[^ ^39^ ^]^ have been documented, their extensive application remains limited. Xenograft models are constrained by the availability of clinical tissue specimens, while transgenic mouse models are challenged by interspecies differences. Recent studies have demonstrated that tumor organoid models effectively replicate the cellular heterogeneity of their corresponding tumor tissues, including tumor cells, cancer‐associated fibroblasts (CAFs), microvasculature, immune cells, and stromal cells.^[^ ^34^, ^40^, ^41^, ^42^, ^43^ ^]^ Consequently, PDOs present a valuable tool for biological investigations and the assessment of therapeutic drug efficacy in ACP. Furthermore, compared to patient‐derived xenograft (PDX) models, PDOs provide advantages such as shorter research cycle times, reduced costs, and enhanced efficiency, rendering them a more practical and scalable platform for preclinical studies.

In a previous study, Wang et al. successfully isolated CD44^+^ ACP stem cell‐like cells (CSLCs) from primary ACP cells utilizing magnetic‐activated cell sorting (MACS).^[^ ^44^ ^]^ Their findings revealed that these CSLCs were capable of forming tumor spheres characterized by positive expression of CD44 and CD133, as well as exhibiting nuclear translocation of β‐catenin. Moreover, the CSLCs demonstrated osteogenic and adipogenic differentiation potential in vitro, alongside tumor initiation capacity in human‐mouse xenograft models.^[^ ^44^ ^]^ These CSLCs underscore the features of tumor stem‐like cells, suggesting their potential as therapeutic targets for ACP. In contrast, the PDOs generated in this study were directly sourced from parental tumors, maintaining the histological and molecular attributes of the original tumor tissues. Our findings indicate that, despite the relatively low proliferation index of ACP, ACP PDOs are capable of being passaged within a week. This is likely attributable to the robust self‐renewal capacity of the stem‐like cells within these PDOs, facilitating their growth even under conditions of low proliferation. Consistent with these findings, the deconvolution analysis corroborated that the predominant cell type present in the ACP PDOs was the whorl‐like epithelium (WE). This cell cluster, characterized by stem cell properties, has been previously identified in ACP.^[^ ^44^ ^]^ The WE is frequently considered to be in a state of senescence, as evidenced by the expression of senescence‐related markers such as P16, P21, P53, GLB1, and γ‐H2A.X, a finding corroborated by prior studies.^[^ ^18^, ^39^, ^45^, ^46^ ^]^ Subsequently, the senescent WE can facilitate the proliferation of the palisade‐like epithelium (PE) through the senescence‐associated secretory phenotype (SASP) (Figure S14A–C, Supporting Information).^[^ ^46^ ^]^ Our further investigations revealed the existence of two distinct subgroups within the WE: one subgroup comprises WE in a senescent state with nuclear translocation of β‐catenin, while the other consists of WE in a proliferative state lacking nuclear translocation of β‐catenin. This differentiation was determined through the expression of the senescence marker Lamin B1, the proliferation marker MCM2, and the classic WNT signaling pathway marker β‐catenin (Figure S14C,D, Supporting Information). This study further suggests that WE with nuclear translocation of β‐catenin not only enhance the proliferation of the PE through SASP, but also stimulate the proliferation of neighboring WE lacking nuclear translocation of β‐catenin. This finding underscores the functional heterogeneity within the WE cell population. Consequently, while some WE in ACP exhibit a senescent state, others can compensate for the loss of WE due to senescence by proliferating, thereby preserving the relative stability of this cell population. Collectively, these findings highlight the critical role of WE in the pathogenesis and progression of ACP. In contrast, the low prevalence of PE in ACP PDOs may be attributed to culture conditions that preferentially support the growth of WE over PE. Consequently, these characteristics substantially enhanced the PDOs’ capacity to accurately reflect the drug response of the original tumors.

This study further demonstrated significant activation of the Wnt and Sonic Hedgehog (SHH) pathways in ACP. Previous research has demonstrated that AXIN2 (associated with the Wnt pathway) and GLI2 and PTCH1 (associated with the SHH pathway) exhibit significant hypomethylation in ACP as evidenced by methylation and gene expression analyses. This hypomethylation correlates with elevated gene expression in the corresponding tumors.^[^ ^47^ ^]^ Microarray analysis also revealed that multiple members of the FGF family, TGF family, and BMP family were significantly upregulated in the cell clusters with nuclear translocation of β‐catenin. This suggests that these secreted signaling molecules may act on themselves or neighbouring cells through autocrine or paracrine mechanisms, which may be related to the tumorigenesis of ACP.^[^ ^48^ ^]^ The RNA sequencing data analysis of ACP PDOs and ACP tissues revealed that the expression levels of these secreted signaling molecules were comparable between the ACP PDOs and ACP tissues, suggesting that ACP PDOs are not only morphologically but also functionally representative of ACP tissues. Our findings further indicate that, despite the relatively low mutational burden characteristic of ACP, PDOs accurately maintain the genetic profile of the original tumors. This genetic fidelity underscores the utility of ACP PDOs as a valuable model for investigating ACP biology and evaluating therapeutic responses.

Recently, two studies concerning ACP have focused on the application of ACP PDOs in precision therapy. For instance, research has demonstrated that the Axl inhibitor Bemcentinib significantly suppresses the proliferation of ACP PDOs, suggesting that Axl could serve as a potential therapeutic target for ACP patients.^[^ ^49^ ^]^ In another study, it was observed that while the B7‐H3‐targeted antibody‐DM1 conjugate exhibited substantial tumor suppression in both ACP cells and ACP PDOs, B7‐H3‐targeted CAR‐T cells displayed limited anti‐tumor efficacy in the organoid model compared to the traditional cell culture model.^[^ ^12^ ^]^ This finding highlights the differences between cell models and PDOs. However, neither study conducted a comprehensive characterization of the ACP PDOs utilized.

At present, although ACP is classified as a benign tumor, its treatment remains a subject of debate due to its propensity to invade adjacent structures with finger‐like protrusions, particularly affecting the hypothalamic‐pituitary axis.^[^ ^1^, ^2^ ^]^ On one hand, gross‐total resection (GTR) can result in damage to surrounding structures, potentially causing complications such as hypothalamic dysfunction, poor quality of life etc. On the other hand, while subtotal resection (STR) alone may substantially elevate the probability of tumor recurrence, its combination with radiation therapy is as effective as GTR at controlling the tumor, with reduced overall morbidity, though radiotherapy is associated with other side effects. Therefore, due to the infiltrative and unpredictable growth pattern of ACP, achieving safe resection is significantly challenging.^[^ ^50^, ^51^, ^52^ ^]^ This challenge has prompted researchers to urgently explore novel treatment strategies for ACP. Several molecular alterations, including β‐catenin, mitogen‐activated protein kinase (MAPK), epidermal growth factor receptor (EGFR), and vascular endothelial growth factor (VEGF), have been identified as proven to be essential potential therapeutic targets of novel therapies for ACP.^[^ ^17^, ^18^, ^19^, ^20^, ^21^, ^44^ ^]^ Targeted therapies addressing these molecular alterations are currently under investigation in various solid tumors.^[^ ^53^, ^54^, ^55^, ^56^ ^]^ However, to our knowledge, only a limited number of drugs targeting MAPK are presently undergoing clinical trials for ACP, including the clinical trial evaluating the therapeutic efficacy of Binemetinib in recurrent ACP (NCT05286788) and the clinical trial investigating Tovarafanib for ACP treatment (NCT05465174), both spearheaded by the CONNECT and PNOC clinical trial consortia.^[^ ^57^ ^]^ Furthermore, a Phase 2 clinical trial is presently underway, utilizing ACTEMRA (Tocilizumab) to evaluate the therapeutic efficacy of IL‐6 receptor inhibition in pediatric patients diagnosed with recurrent ACP,s including those who have undergone surgery and/or radiation therapy (NCT05233397) led by the CONNECT clinical trial consortia.

In the present study, drug sensitivity screening identified Ceritinib, an Food and Drug Administration (FDA)‐approved ALK/IGF‐1R inhibitor, as a potentially effective agent against ACP PDOs. Ceritinib demonstrated potent inhibitory effects across all tested PDO lines, indicating its potential therapeutic value for ACP. Although Ceritinib is currently approved as an ALK inhibitor for the clinical treatment of ALK‐positive patients with advanced metastatic non‐small cell lung cancer (NSCLC).^[^ ^58^, ^59^ ^]^ Notably, the research conducted by Kuenzi et al. identified Ceritinib sensitivity in ALK‐negative lung cancer cell lines and revealed a polypharmacological mechanism involving non‐canonical targets, such as ribosomal protein S6 kinase A3 (RPS6KA3), CAMKK2, and YBX1.^[^ ^60^ ^]^ ALK is not recognized as a major driver mutation in ACP. Immunohistochemical staining and qPCR assays further revealed that the expression of ALK in ACP was significantly lower than that of IGF‐1R. Additionally, IGF‐1R has been reported to play a role in the pathogenesis of ACP.^[^ ^23^, ^24^ ^]^ In this study, we postulated that IGF‐1R serves as the principal target of Ceritinib, a hypothesis that was subsequently confirmed through our research findings. Further mechanistic investigations demonstrated that Ceritinib impedes the proliferation of ACP PDOs by downregulating the IGF‐1R/PI3K/AKT/GSK‐3β/β‐catenin signaling axis. IGF‐1R, a transmembrane receptor tyrosine kinase, consists of two alpha and two beta subunits and primarily mediates the signaling of insulin‐like growth factors IGF‐1 and IGF‐2.^[^ ^61^, ^62^ ^]^ The overexpression of IGF‐1R is associated with the activation of several signaling cascades, including PI3K/AKT/mTOR, Ras/Raf/MEK/ERK, and JAK/STAT pathways, which are implicated in the regulation of tumor cell proliferation, apoptosis, and resistance to chemotherapy in various cancers such as breast cancer, NSCLC, colorectal cancer (CRC), and prostate cancer.^[^ ^63^, ^64^, ^65^, ^66^, ^67^, ^68^, ^69^ ^]^ Activation of the PI3K/AKT pathway facilitates the phosphorylation of GSK‐3β, resulting in the stabilization and nuclear translocation of β‐catenin, thereby activating Wnt signaling.^[^ ^56^ ^]^ Our findings indicate that Ceritinib effectively disrupts this signaling cascade in ACP cells, ultimately resulting in the inhibition of ACP cell growth. Further investigation, particularly through in vivo experiments, is necessary to confirm the anti‐tumor effects of Ceritinib on ACP. Such research may lead to the development of a treatment strategy targeting the upregulated IGF‐1R/PI3K/AKT/GSK‐3β/β‐catenin axis in ACP patients.

According to the FDA's prescribing information for Ceritinib capsules (Zykadia), an oral dosage of 450 mg once daily is recommended. However, the ASCEND‐4 clinical trial, as published in The Lancet, employed a dosage of 750 mg once daily for the treatment of patients with stage IIIB/IV ALK‐rearranged non‐squamous NSCLC. The majority of patients tolerated this higher dosage, with gastrointestinal discomfort being the most frequently reported adverse reaction.^[^ ^58^ ^]^ Furthermore, in a separate phase 1 clinical trial, Ceritinib was administered orally in doses ranging from 50 to 750 mg once daily to patients with advanced cancers characterized by genetic alterations in ALK. The most prevalent adverse events observed were nausea, diarrhea, vomiting, fatigue, and elevated alanine aminotransferase levels. Pharmacokinetic analyses indicated that Ceritinib exposure increased proportionally with the dosage.^[^ ^70^ ^]^ According to the phase 1 clinical study, the oral dosage and pharmacokinetic parameters of Ceritinib in Asian populations are detailed as follows: At an oral dose of 450 mg, the maximal plasma concentration (C_max_) ranged from 858 to 982 µg L^−1^, equivalent to 1.5372 to 1.7594 µm. At an oral dose of 750 mg, the C_max_ ranged from 1220 to 1470 µg L^−1^, equivalent to 2.1858 to 2.6337 µm.^[^ ^71^ ^]^ In this study, the IC_50_ value of Ceritinib for ACP PDOs ranged from 0.20 to 4.50 µm, with 82.35% (14 out of 17) of the values being lower than 2.0 µm. Consequently, when patients administer Ceritinib for the treatment of ACP, the resultant drug concentration in their bodies can achieve levels necessary to effectively target ACP tumor cells, indicating that the IC_50_ value of Ceritinib for ACP PDOs is within a clinically attainable range. In an in vitro study investigating the effects of Ceritinib on hepatocellular carcinoma (HCC) PDOs, it was observed that PDOs harboring the *CTNNB1* mutation exhibited increased sensitivity to Ceritinib compared to their wild‐type counterparts. This was evidenced by a significantly lower IC_50_ value in *CTNNB1 *mutated PDOs (5.33 ± 2.74 µm) relative to *CTNNB1 *wild‐type PDOs (27.28 ± 19.58 µm).^[^ ^72^ ^]^ Furthermore, Ceritinib demonstrated cytotoxic effects in triple‐negative breast cancer (TNBC) cells in both a dose‐ and time‐dependent manner with IC_50_ values of 1.19 µm for MDA‐MB‐453 cells and 1.24 µm for MFM223 cells.^[^ ^73^ ^]^ Additionally, previous research indicated that the IC_50_ value of Ceritinib in treatment‐naive patient‐derived cell lines (PDCs) from a ROS1‐TKI naive NSCLC patient with CD74–ROS1 was 0.412 µm.^[^ ^74^ ^]^ This comparison suggests that the efficacy of Ceritinib in our PDO models is comparable to its potency in other cancer types known to be sensitive to this compound.

ACP frequently invades adjacent structures, particularly the hypothalamic‐pituitary axis.^[^ ^1^, ^2^ ^]^ Both the tumor itself and surgical intervention often result in hypothalamic dysfunction and hypopituitarism in patients with ACP.^[^ ^3^, ^4^, ^5^ ^]^ In pediatric patients, growth hormone deficiency (GHD) frequently leads to stunted growth, necessitating the exogenous administration of growth hormone (GH) to facilitate growth in these individuals.^[^ ^75^, ^76^ ^]^ GH is converted into IGF‐1 in the liver, which poses a potential risk of promoting tumor growth through its action on the IGF‐1R. Although a few previous clinical studies have generally concluded that GH therapy does not increase the recurrence rate of ACP in pediatric patients,^[^ ^77^, ^78^, ^79^ ^]^ these studies have certain limitations. Research has demonstrated that treating primary ACP cells with high IGF‐1R expression using IGF‐1R inhibitors reduces the phosphorylation level of AKT and arrests primary cell growth. In contrast, the growth of ACP cells with low IGF‐1R expression is only marginally affected by IGF‐1R inhibition, suggesting that IGF‐1R may play a role in the proliferation of ACP cells.^[^ ^25^ ^]^ Furthermore, the silencing of IGF‐1R in CD44^+^ CSLCs within ACP has been shown to markedly decrease both the formation of tumor stem cell spheres and the migratory capacity of tumor cells. This indicates that IGF‐1R plays a critical role in maintaining the stemness and facilitating the migration of ACP cells, along with other deleterious biological behaviors.^[^ ^23^ ^]^ Consequently, it is theoretically plausible that GH may indirectly stimulate mitotic activity in ACP cells via IGF‐1. Nonetheless, in clinical settings, GH administration remains relatively safe for pediatric ACP patients. We hypothesize that this safety may be attributed to the relatively weak tumor‐promoting effects of physiological doses of GH on IGF‐1R in ACP cells, which may coincide with the natural progression of the tumor, thereby rendering the tumor‐promoting effects of GH negligible. In this study, the concentration of Ceritinib employed in vitro was comparatively higher, resulting in a pronounced anti‐tumor effect. Thus, employing an IGF‐1R inhibitor such as Ceritinib to target the IGF‐1R signaling pathway may represent a pivotal strategy to mitigate the potential tumor‐promoting risks associated with essential GH replacement therapy.

EGFR inhibitors, such as Dacomitinib and Neratinib, have demonstrated efficacy against ACP PDOs, although their sensitivity varies across different PDO lines. EGFR signaling has been implicated in facilitating ACP tumor cell migration.^[^ ^17^ ^]^ Nevertheless, resistance to EGFR inhibitors remains a prevalent obstacle in cancer treatment.^[^ ^65^ ^]^ Additionally, Dasatinib, an inhibitor of Src/Bcr‐Abl, has shown activity in certain PDO lines. Src family kinases are integral to several cellular processes, including proliferation, adhesion, and migration, and may play a role in the pathogenesis of ACP. The differential drug responses observed among PDO lines underscore the interpatient heterogeneity of ACP and highlight the potential of PDOs in advancing personalized medicine strategies. Conversely, Vismodegib, a SHH pathway inhibitor, exhibits limited efficacy in inhibiting the growth of ACP PDOs, with an IC_50_ value ≈100 times greater than that of Ceritinib. Notably, PDO_38 is entirely unresponsive to Vismodegib, with an estimated IC_50_ value of 8315 µm. Previous studies have demonstrated that Vismodegib treatment leads to the early development of highly proliferative and vascularized undifferentiated tumors in mouse models. This results in a significant reduction in median survival and a marked increase in tumor cell proliferation in both explant cultures and in PDX models.^[^ ^80^ ^]^ These findings suggest that the expression of a target alone is insufficient to confer therapeutic benefit and may, in fact, contribute to harm and tumor progression. Notably, Vismodegib showed a lack of efficacy in ACP PDOs rather than a pro‐tumorigenic effect. This contrasts with in vivo findings, where it promoted tumor growth, a critical observation that highlights the limitations of in vitro systems, which lack a complex microenvironment. It underscores that results from PDO models, while valuable, cannot be directly extrapolated to clinical settings and require in vivo validation.

The two pharmacotyping cases discussed in this study offer preliminary evidence suggesting that drug responses observed in ACP PDOs may correlate with clinical outcomes in patients. Patient 1, who was administered Ceritinib for recurrent ACP, demonstrated tumor regression and pathological changes, reflecting the drug's potent activity observed in our PDOs. Similarly, Patient 2 exhibited a potential reduction in tumor volume following Ceritinib treatment. However, as a preliminary and exploratory observation, the observed significant tumor growth is a major concern, suggesting that the in vivo response is more complex and that Ceritinib cannot be recommended for use without further close study and within a closely monitored clinical trial. These cases also underscore the urgent need for well‐designed, prospective clinical trials to determine if Ceritinib has any true therapeutic role in ACP.

Nevertheless, we recognize certain limitations inherent in our study. First, although PDOs successfully recapitulate key aspects of ACP biology, these ACP PDOs are exclusively epithelial and do not encompass the complete tumor microenvironment, including immune cell components, vascular structures, neurons, microglial cells, and astrocytes. Two primary factors may contribute to this limitation. First, during the cultivation process of PDOs, an artificial selection effect may occur, whereby non‐proliferative cell subpopulations, such as senescent cells and immune cells, are excluded, while only cells with robust proliferative capacity or stem cell characteristics are retained. This phenomenon likely accounts for the predominance of WE cells in the ACP PDOs. Second, the normal brain tissue components at the periphery of the ACP tissue samples obtained from surgery are insufficiently represented. Furthermore, the lack of immune components in PDOs constrains their utility in immunotherapy development. Recent studies have demonstrated the successful construction of cerebral organoids from human pluripotent stem cells,^[^ ^81^ ^]^ suggesting that future research should investigate more intricate co‐culture systems to more accurately replicate the in vivo tumor environment. Additionally, this study did not detect *CTNNB1* mutations in two of the PDO samples by using WES. This absence does not necessarily indicate that the sample is not derived from tumor epithelial cells; rather, it may be attributed to the limited number of PDOs sequenced, the low variant allele frequency (VAF) of *CTNNB1* in the sample, and the limitations of WES depth in identifying very low‐abundance subclonal variations, which was evidenced by the results of Sanger sequencing. Although the genomic analyses provided valuable insights, they were conducted on an exploratory cohort; thus, further studies with larger sample sizes are necessary to comprehensively elucidate the genomic landscape of ACP. It is also crucial to recognize that, despite its advancements, the organoid culture system remains a simplified model and may not fully capture the intricate in vivo tumor biology and microenvironment. Third, the intrinsic characteristics of ACP, characterized by its slow growth and often cystic nature, present challenges in generating the substantial cell numbers necessary for establishing intracranial xenografts. Consequently, the success rate of intracranial tumor formation is low. As a result, this study did not include animal experiments for the formation of intracranial xenografts using PDOs. Furthermore, the pharmacotyping data are derived from only two clinical cases, providing preliminary and hypothetical evidence that necessitates validation through larger‐scale prospective studies. Despite these limitations, our study contributes a valuable resource to ACP research. The establishment of an ACP organoid biobank and the identification of Ceritinib as a potential therapeutic agent represent significant advancements in this field.

In conclusion, we have successfully established a robust PDO platform for ACP that accurately replicates the key characteristics of primary ACP tumors. Through organoid‐based drug screening, Ceritinib was identified as a potent inhibitor of ACP PDO growth, primarily by downregulating the IGF‐1R/PI3K/AKT/GSK‐3β/β‐catenin signaling axis. These findings indicate that IGF‐1R represents a promising therapeutic target for ACP, and ACP PDOs offer substantial potential for preclinical drug evaluation and the development of personalized medicine strategies for this rare and challenging tumor. Prospective clinical trials are necessary to confirm the efficacy of Ceritinib and other IGF‐1R inhibitors in ACP, as well as to investigate the potential of organoid‐based pharmacotyping in guiding personalized treatment strategies. Further research is essential to validate these findings and to explore the clinical applicability of ACP organoid‐based pharmacotyping in informing treatment decisions for ACP patients.

## 4 Conclusion

**Figure 9:**
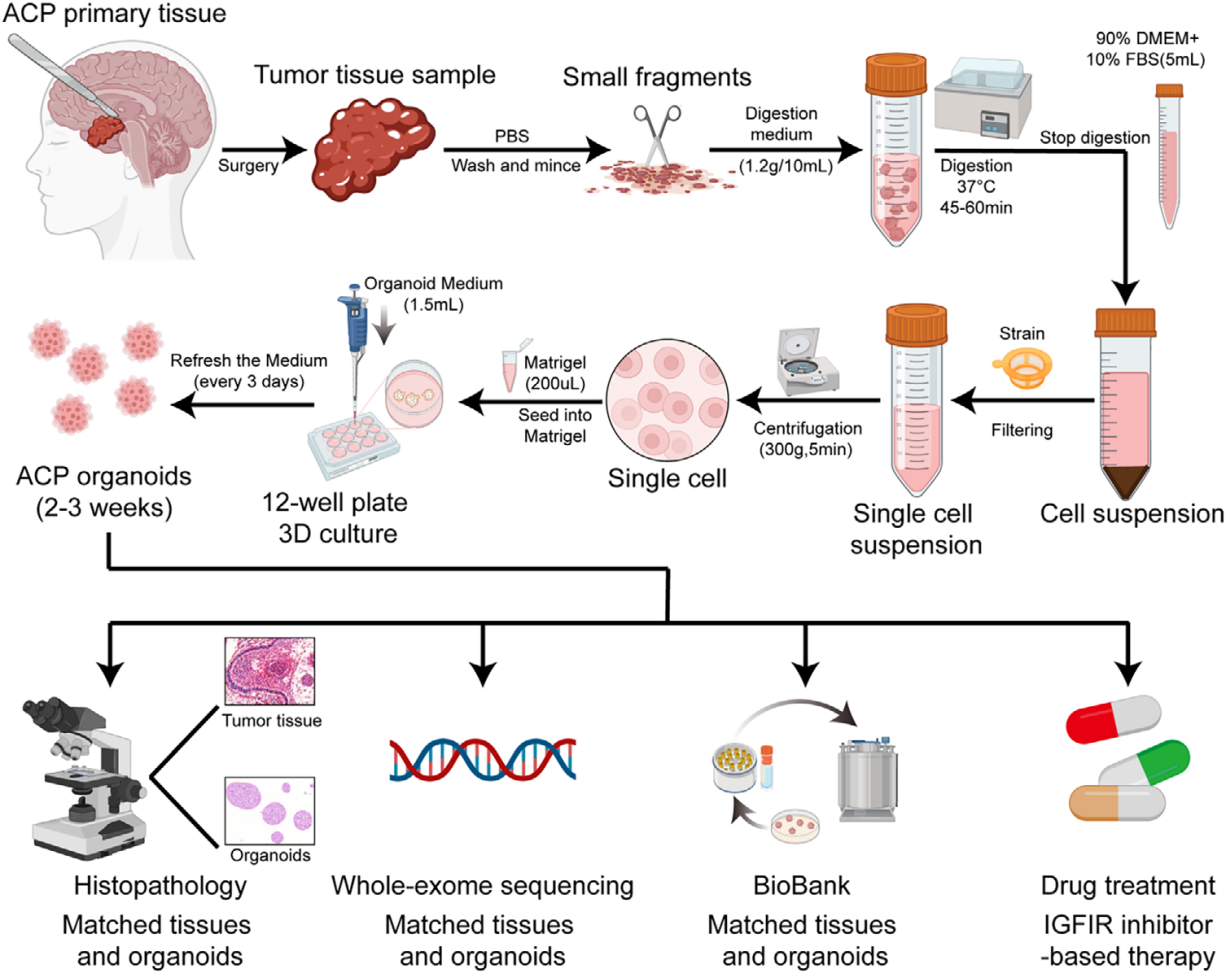
Patient‐derived ACP organoid platform for drug screening and personalized medicine. ACP PDOs faithfully replicate the characteristics of the original tumors, allowing for drug sensitivity testing. This capability significantly enhances preclinical research and aids in the development of personalized treatment strategies.

In conclusion, we have developed and thoroughly validated a robust ACP organoid platform that functions as a high‐fidelity preclinical model for this challenging disease. This significant resource has facilitated the identification of IGF‐1R signaling as a critical therapeutic target in ACP, thereby highlighting Ceritinib as a promising and clinically translatable treatment strategy for affected patients (**Figure** **9**). However, the case study suggests that tumors might grow during Ceritinib treatment before any therapeutic effects appear. Thus, until large‐scale trials verify its effectiveness for ACP patients, Ceritinib should be used cautiously with close monitoring.

## 5 Experimental Section

### Human Tissue Samples and Clinical Case Studies

Human ACP tissue samples were collected intraoperatively from patients undergoing surgical procedures at the Department of Neurosurgery, Nanfang Hospital, Southern Medical University. Before participation, all patients provided written informed consent. The study adhered to the ethical guidelines set forth by the Ethics Committee of Nanfang Hospital, Southern Medical University, and complied with all provisions of the World Medical Association Declaration of Helsinki. Histopathological evaluation confirmed the ACP diagnosis for all samples. An independent histopathologist verified each diagnosis using routine hematoxylin and eosin (H&E)‐stained slides. Additionally, clinicopathological data were gathered, and the clinical information of the patients is detailed in **Table** **1**.

The clinical administration of Ceritinib, informed by PDO pharmaco‐phenotyping, was designed to serve as clinical case studies. This investigation received assessment and approval from the Ethics Committee of Nanfang Hospital, Southern Medical University (approval number: NFEC201096). In accordance with the principles of the Declaration of Helsinki, all patients provided written informed consent. For 3D reconstruction, tumor segmentation was performed in 3D Slicer software (version 5.9.0), selecting the sagittal, coronal, and axial slices with the tumor cross‐section as the region of interest (ROI). Three senior radiologists with ten years of experience in intracranial tumors, blinded to clinical information and pathological subtype, performed image segmentation. The solid and cystic lesions were manually segmented slice by slice on contrast‐enhanced T1‐weighted magnetic resonance imaging (MRI) images, and their volumes were separately calculated. All segmentations were confirmed by a senior radiologist, and disagreements were resolved by consensus.

### Establishment of Patient‐Derived Organoid (PDO) Models of ACP—Tumor Tissue Dissociation

The fresh tumor tissues were initially washed three times with cold phosphate buffer solution (PBS, Solarbio) (pH 7.4) supplemented with 2% penicillin–streptomycin (Thermo Fisher Scientific). Subsequently, the tissues were minced into pieces measuring 1–3 mm^3^ using the Brain Tumor Dissociation Kit (Miltenyi Biotec, Bergisch Gladbach, Germany). The above‐mentioned digestion buffer was added to the tissue fragments in a volume proportional to the tissue size (1.2 g of tumor tissue per 10 mL of digestion buffer). The tumor tissues were then dissociated on a shaker at 37 °C for 45–60 min, with occasional pipetting, until the visible tissue fragments were no longer discernible. Following incubation, the suspensions were combined with a complete medium consisting of 90% Dulbecco's Modified Eagle's Medium (DMEM) and 10% fetal bovine serum (FBS), filtered through 100 µm cell strainers, and centrifuged at 300 g for 5 min. If the suspensions exhibited a visible red coloration, erythrocyte lysis was performed using red blood cell (RBC) lysis buffer (eBiosciences) for an additional 3 min.

### Establishment of Patient‐Derived Organoid (PDO) Models of ACP—Organoid Culture and Passage

The dissociated cell clusters in suspension were quantified using microscopy and subsequently subjected to centrifugation at 300 g for 5 min. The cell clusters were then resuspended in 500 µL of 50% ice‐cold Matrigel (Corning) at a 1:1.5 ratio. Subsequently, 50 µL aliquots of the Matrigel‐cell cluster mixture were dispensed onto prewarmed 12‐well culture plates (Corning) at a density of 6–8 × 10^3^ cells per 50 µL of Matrigel per well. The Matrigel droplets were allowed to solidify in an incubator set at 37 °C with 5% CO_2_ for 30 min. Following solidification, 1.5 mL of organoid culture medium was added to each well, with the medium being replenished every 2 to 3 days. Briefly, the culture medium was composed of Dulbecco's Modified Eagle Medium: Nutrient Mixture F‐12 (DMEM/F12), supplemented with Primocin (100 µg mL^−1^, Invitrogen), ‌N‐2‐hydroxyethylpiperazine‐N'‐2‐ethanesulfonic acid (HEPES, 10 mm, Gibco), GlutaMAX (1X, Gibco), A83‐01 (500 nm, Tocris), Y‐27632 (10 µm, AbMole), N‐acetylcysteine (2 mm, Sigma), nicotinamide (10 mm, Sigma), fibroblast growth factor‐10 (FGF10, 10 ng mL^−1^, PeproTech), B27 supplement (1X, Gibco), Epidermal Growth Factor (EGF, 50 ng mL^−1^, PeproTech), Gastrin I (0.01 µm, Tocris Bioscience), Wnt3A conditioned medium (50 ng mL^−1^, R&D Systems), R‐spondin‐1 conditioned medium (250 ng mL^−1^, R&D Systems), and Noggin conditioned medium (100 ng mL^−1^, R&D Systems). The number of ACP PDOs was assessed by culturing them in 12‐well plates followed by microscopic counting. The size of ACP PDOs was quantified by capturing images with a microscope and measuring the diameters using ImageJ software (NIH, Bethesda, MD, USA). The growth of ACP PDOs was monitored and documented at specified time intervals using an inverted microscope (Olympus IX71).

Organoid passaging was conducted weekly, contingent upon the density and size of the PDOs. Initially, the PDOs were resuspended in 0.25% cold trypsin (Invitrogen) through pipetting following the removal of the culture medium, and subsequently incubated at 37 °C for 5 min. Additional pipetting and incubation were performed as necessary. Thereafter, the cells were centrifuged at 300 g for 5 min, resuspended in Matrigel at a 1:1.5 ratio, and seeded into prewarmed 12‐well culture plates, following the previously described culturing protocol. For the preparation of frozen stocks, the trypsin‐digested PDOs were resuspended in freezing medium (New Cell & Molecular Biotech Co. Ltd) and cryopreserved in liquid nitrogen using a gradient cooling method. The cultured PDOs were utilized for a variety of analyses, including histopathological examination, whole exome sequencing (WES), drug sensitivity assays, RNA sequencing, Western blot analysis, and quantitative real‐time PCR (q‐RT‐PCR), among others. However, due to the predominantly cystic nature of ACP, the tumor tissues obtained were relatively small. Furthermore, the drug screening process required completion within a limited timeframe, necessitating the allocation of a substantial proportion of PDOs to drug testing. Consequently, only a limited number of cases had sufficient PDOs available to conduct multiple experimental procedures. Therefore, the prioritization for the utilization of PDOs was established as follows: 1) RNA sequencing and WES; 2) drug sensitivity testing; 3) cryopreservation; 4) continued passaging; 5) histological analysis; and 6) Western blot analysis, among others.

### Drugs and Treatments

Following dissolution of the IGF‐1R inhibitor Ceritinib (MedChemExpress, HY‐15656) in DMSO, the ACP PDOs were exposed to 2 µm Ceritinib for a duration of one week, or alternatively, they were treated with 2 µm Ceritinib 36 h post‐siRNA transfection. In contrast, the STAM4 cells were subjected to 2 µm Ceritinib for either 48 or 96 h. The PI3K agonist 740Y‐P (Selleck, S7865) was also dissolved in DMSO, and the ACP PDOs were pretreated with 30 µg mL^−1^ of 740Y‐P for 12 h before the administration of Ceritinib. For the treatment involving recombinant human IGF‐1 protein (MedChemExpress, HY‐P7018), the ACP PDOs were pretreated with Ceritinib for 12 h before the administration of 100 ng mL^−1^ IGF‐1.

### RNA Interference

Small interfering RNAs (siRNAs) targeting IGF1R along with a scrambled siRNA, were procured from Applied Biosystems (Foster City, CA, USA). The specific sequences utilized in this investigation are detailed in Table S1 (Supporting Information). To mitigate potential off‐target effects, ACP PDOs were transfected with three distinct IGF1R‐targeting siRNAs and one non‐specific control siRNA, employing Lipofectamine RNAiMAX (Invitrogen) in accordance with the manufacturer's protocol. Briefly, the siRNA transfection solution was formulated by combining *IGF‐1R*‐specific or control siRNA with RNAiMAX (Invitrogen) in DMEM (Gibco) supplemented with 10% FBS (Gibco). This solution was directly applied to the PDOs following a two‐day culture period in a 24‐well plate. Subsequently, the medium was refreshed with new organoid culture medium 48 h post‐transfection.

### Organoid Drug Response Assay

For the analysis of drug response in ACP PDOs, organoids were harvested 4 to 5 days post‐passaging and filtered through a 100 µm cell strainer (Corning) to remove larger organoids. The filtered organoids were then resuspended in a 2% Matrigel/organoid culture medium at a concentration of 15000–20000 organoids per milliliter and distributed into ultralow‐attachment 96‐well plates (Corning) in triplicate. After 24 h of incubation, a four‐fold serial dilution of each drug compound was applied, with concentrations ranging from 4 to 0.0039 µmol L^−1^ or 800 to 0.78125 µmol L^−1^, contingent upon the specific properties of each drug. The maximum concentration of DMSO used was 1%. Following a 7‐day incubation period with the drugs, cell viability was assessed using the CellTiter‐Glo 3D assay (Promega), which measures ATP content as a surrogate marker, in accordance with the manufacturer's instructions. The results were normalized against vehicle controls. Data analysis was conducted using GraphPad Prism 9.0 software, and IC_50_ values were determined through nonlinear regression (curve fitting) using the equation for log(inhibitor) versus normalized response (variable slope). The specific targeted drugs utilized in this organoid drug sensitivity assay are detailed in Table S2 (Supporting Information).

### Histology, Immunohistochemistry, and Immunofluorescence Staining

The parental tumor tissues were fixed in 4% paraformaldehyde (PFA) and subsequently embedded in paraffin following standard protocols. For the processing of PDOs, the samples were resuspended in ice‐cold PBS, subjected to centrifugation, and fixed in 4% PFA for a duration of 24 h at 4 °C. Following fixation, the PDOs were washed with PBS, transferred to 70% ethanol, and processed for paraffin embedding. Subsequent steps included sectioning, deparaffinization, dehydration, and staining with hematoxylin and eosin (H&E). Paraffin sections with a thickness of 3 µm were utilized for all analyses, and H&E staining was performed for histopathological evaluation.

For immunohistochemical staining, following paraffin embedding, sectioning, deparaffinization, and dehydration, antigen retrieval was conducted by heating the sections for 20 min in either sodium citrate buffer (pH 6.0; Solarbio) or ethylenediaminetetraacetic acid (EDTA) (pH 8.0; ZSGB‐BIO) using a microwave. Subsequently, slides of parental ACP tissues and ACP organoid samples were incubated with primary antibodies at 4 °C overnight. On the following day, the slides were incubated with the appropriate secondary antibody (Beijing Biosynthesis Biotechnology Co. Ltd.; Beijing, China) for 40 min, followed by staining with 3,3′‐diaminobenzidine (DAB) (ZSGB‐BIO) and counterstaining with hematoxylin. The primary antibodies utilized are detailed in Table S3 (Supporting Information).

For the immunofluorescence staining procedure, following paraffin embedding, sectioning, deparaffinization, and dehydration, antigen retrieval was conducted by heating the sections for 20 min in either sodium citrate buffer (pH 6.0; Solarbio) or EDTA (pH 8.0; ZSGB‐BIO) using a microwave. The parental ACP tissues and ACP organoid samples were incubated with primary antibodies at 4 °C overnight. Subsequently, the primary antibodies were detected using corresponding fluorescent secondary antibodies (# A‐11001, # A‐11012; Thermo Fisher Scientific) at a dilution of 1:1000. Nuclei were counterstained with 4′,6‐diamidino‐2‐phenylindole (DAPI, Thermo Fischer Scientific) for 5 min at room temperature (RT). The primary and fluorescent secondary antibodies utilized for immunofluorescence staining in this study are detailed in Table S3 (Supporting Information). Imaging of PDOs and their parental tumors was performed using an OLYMPUS BX63 microscope (Olympus). Image processing was conducted using Olympus cellSens software and Adobe Photoshop CS4 (8‐bit).

### Whole Exome Sequencing (WES)

Genomic DNA samples were prepared utilizing the DNeasy Blood & Tissue Kit (QIAGEN) in accordance with the manufacturer's protocol. Initial quantification and quality assessment of the DNA were performed to ascertain the integrity and purity of the genomic DNA. For library preparation, 1 µg genomic DNA per sample was used, following fragmentation into 180–280 base pair segments via a hydrodynamic shearing system (Covaris, Massachusetts, USA). The degradation of DNA was evaluated through agarose gel electrophoresis, while the fragment size distribution was analyzed using the Agilent 4200 TapeStation system (Agilent, Santa Clara, CA, USA). DNA purity was assessed using a NanoDrop One/OneC spectrophotometer (Thermo Scientific, Waltham, MA, USA). For the construction of libraries for whole‐exome sequencing, 1 µg of genomic DNA was enzymatically sheared using dsDNA Fragmentase. The process commenced with sequential end‐repairing, 3′‐end A‐tailing, and ligation with indexed adapters, followed by size selection utilizing Agencourt AMPure XP beads (Beckman Coulter Inc., Brea, CA, USA). Library construction was executed using the KAPA Library Preparation Kit (Kapa Biosystems, Inc., USA) in accordance with the manufacturer's protocol. Subsequent purification and quality assessment were conducted using Agencourt AMPure XP beads and the NanoDrop One/OneC spectrophotometer. Target enrichment was achieved through hybridization using the Agilent SureSelect Human All Exon V6 (Agilent Technologies, CA, USA). Post‐capture amplification and library quality analysis were then performed. For the library quality analysis, the concentration of the constructed library was determined using a Qubit 3.0 Fluorometer, and its molar concentration was quantified via QuantStudio 3 qPCR. The distribution of library fragments was assessed with the Agilent 4200 TapeStation. Finally, DNA sequencing was conducted on the Illumina NovaSeq 6000 system employing paired‐end 150 base pair reads, adhering to the manufacturer's instructions.

### Whole Exome Sequencing (WES) Data Analysis

The sequencing data underwent quality control using FastQC (version 0.11.8), followed by adapter trimming with Trim Galore (version 0.5.0). The sequence reads were aligned to the human reference genome GRCh38 utilizing the Burrows‐Wheeler Alignment with maximal exact matches (BWA‐MEM) (version 0.7.17).^[^ ^82^ ^]^ Quality control statistics were aggregated and summarized using MultiQC (version 1.7).^[^ ^83^ ^]^ Alignment files were sorted, and statistics were extracted from BAM files using SAMtools (version 0.1.9).^[^ ^84^, ^85^ ^]^ Data preprocessing was conducted with the Genome Analysis Toolkit (GATK) (version 4.1.1.0) in accordance with the best practice guidelines.^[^ ^86^, ^87^, ^88^ ^]^ DNA variants were comprehensively annotated using Annotate Variation (ANNOVAR),^[^ ^89^ ^]^ which assessed gene structural impacts, cross‐referenced known polymorphisms with population and disease databases, predicted pathogenicity through integrated algorithms, and incorporated functional genomic annotations. The subsequent characterization of the mutational landscape included profiling the trinucleotide‐context mutation spectrum and deconvoluting biological signatures using Sigminer.^[^ ^90^ ^]^ This analysis, based on a 96‐substitution model that considers ±1 base pair flanking sequences, identified distinct mutational signatures indicative of specific etiological processes, such as DNA repair deficiencies, environmental exposures, and enzymatic activities, which contribute to the patterns of somatic single‐nucleotide variants (SNVs) in tumorigenesis. Somatic SNVs were identified using MuTect (version 1.1.7) in a tumor‐only mode.^[^ ^91^ ^]^ To differentiate potential somatic mutations from germline polymorphisms, all variants were meticulously filtered against the Genome Aggregation Database (gnomAD, version 3.1.2), excluding any variant with a minor allele frequency (MAF) greater than 0.001 in any cataloged population, including the East Asian cohort (*n* = 19952). The functional impact of the remaining non‐synonymous SNVs was predicted using in silico algorithms, including Sorting Intolerant From Tolerant (SIFT, http://sift.jcvi.org) and Polymorphism Phenotyping v2 (PolyPhen‐2, http://genetics.bwh.harvard.edu/pph2/). Variants predicted as “deleterious” by SIFT or “probably damaging” by PolyPhen‐2 were prioritized as candidate driver mutations. The identified somatic variants were systematically analyzed against four established cancer driver gene repositories: the Significantly Mutated Genes (SMG) 127‐gene list, the Comprehensive Driver Gene Set (435 genes), the Cancer Gene Census (CGC),^[^ ^92^ ^]^ and the Vogelstein 125 core driver genes.^[^ ^93^ ^]^ This integrative approach prioritized mutations with established oncogenic significance. Driver mutations were characterized as somatic genetic alterations that provide a selective growth advantage to tumor cells, thereby directly contributing to cancer initiation, progression, or maintenance. Building upon this annotated genomic landscape, the tumor mutational burden (TMB) was defined as the presence of somatic SNVs and small insertions and deletions (indels) located within the coding region and its 20 base pairs upstream and downstream. Meanwhile, microsatellite instability (MSI) status was determined based on the indel frequencies at microsatellite loci.

### Sanger Sequencing Analysis

Genomic DNA was extracted from frozen samples of ACP PDOs and the corresponding parental tumor tissues using proteinase K digestion followed by phenol/chloroform extraction. A polymerase chain reaction (PCR) was conducted to amplify a 799‐base pair (bp) fragment encompassing codons A32, S33, G34, I35, S37, T41, and S45 within exon 3 of the *CTNNB1* gene. The primer sequences utilized were as follows: Forward primer: 5′‐CACTGAGCTAACCCTGGCTATCA‐3′, Reverse primer: 5′‐CGTGTGGCAAGTTCTGCATCAT‐3′. Briefly, the PCR amplification was carried out in a 25 µL reaction mixture, which included 100 ng of genomic DNA, 2.5 µL of 10X PCR buffer, 0.8 µL of 10 mm deoxynucleotide triphosphates (dNTPs), 0.25 µL each of the forward and reverse primers, and 0.8 µL of high‐fidelity Taq polymerase (Applied Biosystems). The initial denaturation was conducted at 95 °C for 5 min, followed by 40 amplification cycles comprising denaturation at 95 °C for 60 s, annealing at 60 °C for 45 s, and extension at 72 °C for 1.5 min, with a final extension at 72 °C for 12 min. The products were analyzed via agarose gel electrophoresis and subsequently purified using the MinElute PCR Purification Kit (Qiagen). Bidirectional sequencing was carried out on an ABI‐3730 sequencer (Applied Biosystems). The mutations identified in the *CTNNB1* gene were evaluated using MutationTaster (http://www.mutationtaster.org/) for disease‐causing potential of candidate sequence alterations.

### RNA‐Seq Analysis—Sample Collection and RNA‐Seq Analysis Preparation

Briefly, to elucidate the underlying mechanisms of Ceritinib's anti‐tumor effects on ACP PDOs, both untreated (Control, *n* = 6) and Ceritinib‐treated (Ceri, *n* = 6) ACP PDOs were prepared and subjected to sequencing analysis. For RNA sample preparation, 1 µg of RNA per sample was utilized. Sequencing libraries were constructed using the NEBNext Ultra RNA Library Prep Kit for Illumina (NEB, USA) in accordance with the manufacturer's instructions, with index codes incorporated to assign sequences to each respective sample. Initially, mRNA was isolated from total RNA via poly‐T oligo‐attached magnetic beads. Fragmentation was induced using divalent cations at elevated temperatures in the NEBNext First Strand Synthesis Reaction Buffer (5×). The synthesis of first‐strand cDNA was conducted using random hexamer primers and M‐MuLV Reverse Transcriptase (RNase H^–^). Subsequently, second‐strand cDNA synthesis was executed using DNA polymerase I and RNase H, with any remaining overhangs being converted into blunt ends through exonuclease/polymerase activity. Following the adenylation of the 3′ ends of DNA fragments, the NEBNext Adaptor, characterized by its hairpin loop structure, was ligated to facilitate subsequent hybridization. To selectively enrich for cDNA fragments ranging from 250 to 300 base pairs in length, the library fragments underwent purification using the AMPure XP system (Beckman Coulter, Beverly, USA). Subsequently, 3 µL of USER Enzyme (NEB, USA) was applied to the size‐selected, adaptor‐ligated cDNA at 37 °C for 15 min, followed by a denaturation step at 95 °C for 5 min prior to PCR amplification. The PCR was conducted utilizing Phusion High‐Fidelity DNA polymerase, along with Universal PCR primers and Index (X) Primer. Finally, the PCR products were purified using the AMPure XP system, and the quality of the library was evaluated using the Agilent Bioanalyzer 2100 system.

### RNA‐Seq Analysis—Clustering and Sequencing

The index‐coded samples underwent clustering utilizing a cBot Cluster Generation System, employing the TruSeq PE Cluster Kit v3‐cBot‐HS (Illumina), in strict adherence to the manufacturer's protocols. Subsequent to cluster generation, the library preparations were sequenced on an Illumina NovaSeq platform, resulting in the production of 150 base pair paired‐end reads.

### RNA‐Seq Analysis—Quality Control

Initially, raw data in FASTQ format were processed using in‐house Perl scripts. During this stage, clean data were generated by eliminating reads containing adapters, reads with ploy‐N sequences, and low‐quality reads from the raw dataset. Concurrently, the Q20, Q30, and GC content metrics of the clean data were calculated. Subsequent analyses were conducted using this high‐quality, clean data.

### RNA‐Seq Analysis—Reads Mapping to the Reference Genome

The reference genome and gene model annotation files were obtained directly from the genome database website. The reference genome index was constructed utilizing Hisat2 (version 2.0.5), and paired‐end clean reads were subsequently aligned to the reference genome using Hisat2 version 2.0.5.

### RNA‐Seq Analysis—Quantification of Gene Expression Levels

Feature Counts version 1.5.0‐p3 was employed to quantify the number of reads aligned to each gene. Subsequently, the fragments per kilobase of exon per million mapped fragments (FPKM) for each gene were calculated, taking into account the gene's length and the corresponding read count.

### RNA‐Seq Analysis—Differential Expression Analysis

Differential expression analysis between the two groups was conducted utilizing the DESeq2 R package (version 1.16.1). To control the false discovery rate, *p*‐values were adjusted according to the Benjamini‐Hochberg procedure. Genes identified by DESeq2 as having an adjusted *p*‐value of less than 0.05 and │log_2_Fold change│greater than 0.585 were classified as differentially expressed. Subsequently, Gene Set Enrichment Analysis (GSEA) was employed for functional annotation.

### RNA‐Seq Analysis—Comparison of the Gene Transcriptional Expressions

To compare the transcriptional level differences of the FGF family, BMP family, TGF family, WNT signaling pathway, and SHH signaling pathway in ACP PDOs and primary ACP tissues, the bulk RNA‐sequencing data of ACP PDOs and the RNA sequencing data of ACP tissues in the Gene Expression Omnibus (GEO, https://www.ncbi.nlm.nih.gov/) database (accession no. GSE94349) were analyzed. Briefly, 24 ACP tissue samples from the dataset GSE94349 were included in this analysis. First, the RNA sequencing data of ACP PDOs were uniformly standardized using the aveLogCPM() function of edgeR package (version 4.4.2). Subsequently, the ComBat_seq() function from the sva package (version 3.56.0) was used to remove the batch effects from the RNA sequencing data of ACP PDOs and ACP tissues. Finally, data visualization was performed using the pheatmap package (version 1.0.12) within the R (version 4.4.1) environment.

### Cytoplasmic and Nuclear Protein Extraction

The nuclear and cytoplasmic proteins from ACP organoid samples, subjected to various treatments (either 2 µm Ceritinib alone or in combination with 30 µg mL^−1^ 740Y‐P), were isolated using the NE‐PER Nuclear and Cytoplasmic Extraction Kit (Thermo Scientific, Rockford, IL, USA), following the manufacturer's instructions. Briefly, a cytoplasmic protein lysate, consisting of Cytoplasmic Extraction Reagent I (CER I), protein phosphatase inhibitor, and protease inhibitor phenylmethylsulfonyl fluoride (PMSF) at a ratio of 100:1:1, was introduced to the ACP PDOs across different experimental groups. Subsequently, the samples were homogenized on ice and incubated for 10 min. Following an initial vortexing period of 5 s, cytoplasmic protein lysate CER II was introduced, and vortexing was continued for an additional 5 s. The mixture was then incubated on ice for 1 min. Subsequently, the homogenate underwent centrifugation at 16 000 g min^−1^ for 5 min at 4 °C, after which the supernatant was carefully collected as the cytoplasmic fraction. The remaining pellets were then resuspended in a nucleolysis solution, consisting of Nuclear Extraction Reagent (NER), a protein phosphatase inhibitor, and the protease inhibitor PMSF in a ratio of 100:1:1. This resuspension was maintained on ice for 50 min, with intermittent oscillation for 15 s every 10 min. Following this, the mixture was centrifuged at 16 000 g min^−1^ for 10 min at 4 °C, and the supernatant was collected as the nuclear fraction. The purity of each fraction was verified by assessing the expression of specific markers, including Lamin B1 and β‐tubulin. The Western blot analysis was conducted as outlined in Section **5.12. Western Blotting Assay**.

### Western Blotting Assay

The cultured ACP PDOs from various experimental groups, which were subjected to treatments including 2 µm Ceritinib, 2 µm Ceritinib combined with 30 µg mL^−1^ 740Y‐P, siIGF‐1R, siIGF‐1R with 2 µm Ceritinib, 100 ng mL^−1^ IGF‐1, and 100 ng mL^−1^ IGF‐1 with 2 µm Ceritinib, were resuspended in ice‐cold PBS. They were then centrifuged at 300 g for 3 min, a process repeated twice to achieve a total of three washing steps, thereby ensuring the removal of residual Matrigel. Subsequently, the PDOs from these groups, along with STAM4 cells treated with or without 2 µm Ceritinib, were lysed using RIPA lysis buffer (Beyotime) supplemented with a protease/phosphatase inhibitor cocktail (Coolaber), following the manufacturer's instructions. The lysates underwent sonication for 10 min and were then centrifuged at 4 °C for 15 min at 12 000 g. Protein concentrations were quantified using the BCA Protein Assay Kit (Solarbio), after which the protein samples were heated at 100 °C for 5 min. Denatured protein samples, each containing 30 µg per lane, were resolved through 10% sodium dodecyl sulfate‐polyacrylamide gel electrophoresis (SDS‐PAGE) and subsequently transferred onto 0.22 µm/0.45 µm polyvinylidene fluoride (PVDF) membranes (MERCK Millipore). The PVDF membranes were blocked with 5% bovine serum albumin (BSA; Sigma‐Aldrich) for 1 h at a temperature range of 25 °C–28 °C, followed by incubation with primary antibodies at 4 °C overnight. Subsequently, the membranes were incubated with horseradish peroxidase (HRP)‐conjugated goat anti‐mouse IgG or goat anti‐rabbit IgG antibodies (Jackson ImmunoResearch) for 1 h at 25 °C–28 °C. The detection of protein bands was performed using the Enhanced Chemiluminescence (ECL) Plus blot kit (MERCK Millipore), and the bands were scanned using a ChemiDoc XRS System and analyzed with the ImageJ software (NIH). Quantification of Western blot results was achieved by assessing the densitometry values of immunoreactive bands corresponding to the target proteins, normalized against a loading control. All samples for Western blot analysis were loaded in duplicate, and each analysis was conducted a minimum of three times. Statistical evaluations were based on the results from the duplicate samples across at least three independent experiments. Details of the primary and secondary antibodies utilized in the Western blot analysis are provided in Table S3 (Supporting Information).

### RNA Extraction and Quantitative Real‐Time PCR

Total RNA was isolated from primary ACP tissues, cultured ACP PDOs, and STAM4 cells. The ACP PDOs and STAM4 cells were subjected to various treatment conditions: untreated, treated with 2 µm Ceritinib, or treated with a combination of 2 µm Ceritinib and 30 µg mL^−1^ 740Y‐P for ACP PDOs, and untreated or treated with 2 µm Ceritinib for STAM4 cells. The extraction was conducted using the RNAiso Plus reagent (TaKaRa) following the manufacturer's instructions. Subsequently, first‐strand cDNA synthesis was performed via reverse transcription using the PrimeScript RT reagent Kit (TaKaRa) with oligo (dT) primers, and the resulting cDNA was stored at −20 °C. RT‐PCR analysis was carried out employing the TB Green Premix Ex Taq II (TaKaRa) on an Applied Biosystems 7500 Fast Real‐Time PCR System (Applied Biosystems, Foster City, CA, USA). Each assay was conducted in triplicate, and the expression levels were normalized against glyceraldehyde 3‐phosphate dehydrogenase (GAPDH) as an internal control. The relative expression levels of the target genes were quantified using the 2^‐ΔΔCt^ method. Details of the specific primers utilized in this study are provided in Table S4 (Supporting Information).

### Cell Culture

The STAM4 cell line, an immortalized ACP cell line previously established by our research group,^36^ was cultured in KGM Gold Keratinocyte Growth Medium (Lonza Ltd., Basel, Switzerland) within a humidified incubator maintained at 5% CO_2_. Authentication of the STAM4 cells was performed using short tandem repeat (STR) analysis, Sanger sequencing, and WES, confirming the absence of mycoplasma contamination.

### Cell Proliferation Analysis

Cell proliferation was assessed utilizing a Cell Counting Kit‐8 (CCK‐8; Dojindo, Kumamoto, Japan) assay, conducted across three independent experiments. STAM4 cells were seeded into 96‐well culture plates at a density of 4 × 10^3^ cells per well and incubated at 37 °C in a humidified atmosphere containing 5% CO_2_ and 95% air for durations of 24, 48, 72, and 96 h, both in the presence and absence of 2 µm Ceritinib. On days 1, 2, 3, and 4, the original medium was replaced with a mixture comprising 10% CCK‐8 solution and 90% culture medium. Subsequently, the STAM4 cells were incubated at 37 °C for an additional 2 h, The level of formazan produced was quantified by measuring the optical density (OD) at 450 nm. Finally, cell growth curves were constructed based on the absorbance values obtained.

### Cell Cycle Analysis

The impact of Ceritinib on the cell cycle was validated using flow cytometry. In brief, STAM4 cells were exposed to either 2 µm Ceritinib or a control for 48 h, after which they were collected in ice‐cold PBS and subsequently fixed with 70% cold ethanol at 4 °C overnight. The cells were then stained with a 50 µg mL^−1^ propidium iodide (PI) solution (Keygentec, Nanjing, China) for 30 min in the dark. The DNA content of the cells in each group was subsequently analyzed using flow cytometry (FACS Calibur, Becton Dickinson).

### Cell Apoptosis Assay

The STAM4 cells were subjected to treatment with 2 µm Ceritinib or left untreated for a duration of 48 h. Following this incubation period, the cells were collected and subjected to staining with PI and annexin V‐fluorescein isothiocyanate (FITC) to facilitate apoptotic analysis. The proportion of apoptotic cells was determined by summing the percentages of early and late apoptotic cells, which were identified in the lower right and upper right quadrants, respectively.

### Single‐Cell RNA Sequencing (scRNA‐seq) Data Acquisition and Re‐Analysis

The recently published single‐cell RNA‐sequencing dataset of human ACP tissues was utilized to validate the expression of the *IGF1R* and *ALK* genes. This dataset is available through the OMIX platform at the China National Center for Bioinformation/Beijing Institute of Genomics, Chinese Academy of Sciences (https://ngdc.cncb.ac.cn/omix:accession no. OMIX001061) and corresponds to the published article titled “Single‐cell RNA sequencing highlights intratumor heterogeneity and intercellular network featured in adamantinomatous craniopharyngioma”.^36^ Quality control, dimensionality reduction, and clustering were conducted using Seurat (version 5.1.0, https://github.com/satijalab/seurat) within the R statistical environment (version 4.4.1), adhering to the standards set forth in the original publication. Briefly, the sequencing outputs were provided as demultiplexed fastq files and processed into expression matrices using the “multi” command in CellRanger (10x Genomics, version 6.1.1). Expression data, including gene, protein, and hashtag information, were imported using the Read10X() function in Seurat (version 5.1.0). Subsequently, a SeuratObject was generated via the CreateSeuratObject() function, retaining only those cells expressing a minimum of 500 genes and genes expressed in at least three cells. Cell inclusion criteria also mandated a distinct gene expression range between 200 and 4000. Further filtration of the remaining genes and cells was conducted to exclude doublets/multiplets by removing cells with high gene counts (>5500) and to eliminate dead cells by excluding those with a high proportion of mitochondrial genes (>10%). Transcript expression was normalized using the LogNormalize option within the NormalizeData() function in Seurat (version 5.1.0). Cell clustering was performed using the FindNeighbors() function with the first 20 principal component dimensions, followed by the FindClusters() function at a resolution of 0.2 in Seurat (version 5.1.0). The FindAllMarkers() function (test.use = Wilcox) in Seurat (version 5.1.0) was employed to identify marker genes for each cluster. For each cluster, positive markers were compared with other cell groups, with a significance threshold set at *P*<0.05 and |log2 foldchange|>0.25. Specific gene sets were sourced from the Molecular Signature Database (SingleR; https://www.bioconductor.org/packages/release/bioc/html/SingleR.html). The cell types for each individual cell were visualized through a t‐distributed stochastic neighbor embedding (t‐SNE) plot. Dimensionality reduction was executed using the t‐SNE() function. Annotation of single‐cell RNA sequencing (scRNA‐seq) data (bioconductor.org) was conducted utilizing SingleR. Epithelial cells (*n* = 22816) were isolated using the subset() function based on marker gene expression and subsequently reclustered as previously described. Average gene expression levels were computed using the AverageExpression function in Seurat (version 5.1.0). Data visualization was performed using ggplot2 (version 3.5.1) and Seurat (version 5.1.0) within the R (version 4.4.1) environment. The final clustering results were projected onto a t‐SNE plot using the RunTSNE() function, and gene expression was illustrated using the VlnPlot() function and DotPlot() function.

### Deconvolution Analysis

To estimate the cell‐type proportions within the ACP organoid RNA‐seq dataset, two deconvolution methodologies were utilized: Cell‐type Identification By Estimating Relative Subsets Of RNA Transcripts (CIBERSORT) and MUlti‐Subject Single Cell deconvolution (MuSiC). The original implementations of CIBERSORT and MuSiC were sourced from https://cibersortx.stanford.edu/ (via a Docker image) and https://github.com/xuranw/MuSiC (as an R package), respectively. For the CIBERSORT deconvolution analysis, a single‐cell reference matrix file specific to epithelial cells was employed, which comprising three subtypes (WE, KE, PE), to construct a custom signature matrix. Subsequently, the gene expression and cell fraction analysis modules to the RNA‐seq data were applied. For the analysis of cell fractions, the proportions of distinct cell subpopulations were quantified using RNA‐seq expression profiles. Cell‐type‐specific expression profiles were inferred from bulk tissue transcriptomes through the application of the High‐Resolution mode for gene expression. This analysis yielded estimates of gene expression variation at the sample level among different cell types, facilitating the exploration of gene expression changes across diverse cellular subpopulations. For the MuSiC deconvolution analysis, RNA‐seq data counts and single‐cell profiles derived from scRNA‐seq data were input into the MuSiC algorithm, executed in R (version 4.4.1). Cell types identified from scRNA‐seq were based on established categorizations. The estimated proportions of cell types for each sample were obtained using the music_prop() function. These estimated proportions were subsequently normalized to sum to one across the included cell types.

### Statistical Analysis

Statistical analyses were conducted utilizing GraphPad Prism (version 9.0, GraphPad Software, San Diego, CA) and SPSS software (version 22.0, IBM, Chicago, IL, USA). These analyses encompassed both descriptive statistics and tests of statistical significance, as presented in this study. Experimental data derived from a minimum of three independent replicates, are presented as mean ± standard error of the mean (SEM). Each experiment incorporated at least three biological replicates. Data sets were assessed for normality using the Kolmogorov‐Smirnov test or the D'Agostino‐Pearson omnibus test, as appropriate. For data exhibiting a normal distribution, comparisons between two groups were performed using an unpaired two‐tailed Student's *t*‐test, whereas comparisons involving more than two groups were conducted using one‐way ANOVA followed by Tukey's post hoc test. To analyze the differences among multiple groups across more than one variable, a two‐way ANOVA was conducted, followed by Tukey's post hoc test. The dose‐response curves depicted in Figure 4C–J,L,M, and Figure S5A–P (Supporting Information) were generated using Microsoft Excel and GraphPad Prism (version 9.0, GraphPad Software, San Diego, CA). The cell viability results are presented as mean ± SEM, with drug concentrations transformed using the log_10_ method. Quantification of Western blot experiments was performed using ImageJ software (NIH, Bethesda, MD, USA, https://imagej.nih.gov/ij/). Statistical significance was determined at a threshold of *p* < 0.05, with significance levels denoted as *p < 0.05, **p < 0.01, and ***p < 0.001. Comprehensive statistical details, including *p*‐values and sample sizes (*n*), are provided in the figure legends.

## Conflict of Interest

The authors declare no conflict of interest.

## Supporting information



Supporting Information

## Data Availability

The data that support the findings of this study are available from the corresponding author upon reasonable request.
