# Supplementary material for: A Patient‐Derived Organoid Biobank of Adamantinomatous Craniopharyngioma as a Platform for Drug Discovery
Source: Adv Sci (Weinh). 2025 Nov 18;13(7):e03924. doi: 10.1002/advs.202503924 (PMC12866856; doi:10.1002/advs.202503924)
Supplement: Supplementary file 1 — Supporting Information [file ADVS-13-e03924-s001.docx]

Supporting Information for

A Patient-Derived Organoid Biobank of Adamantinomatous Craniopharyngioma as a Platform for Drug Discovery

*Huarong Zhang†, Chaohu Wang†, Jun Fan†, Zexin Chen†, Haoying Yu†, Yawen Bai, Tingcheng Zhang, Qianchao Zhu, Yiwen Feng, Peirong Niu, Jiaqian Chen, Liping Yang, Xueying Li, Lei Yu, Songtao Qi*, Yi Liu**

H. Zhang, C. Wang, J. Fan, Y. Bai, T. Zhang, Q. Zhu, Y. Feng, P. Niu, J. Chen, L. Yang, X. Li, L. Yu, S. Qi, Y. Liu

Department of Neurosurgery

Nanfang Hospital

Southern Medical University

Guangzhou 510515, Guangdong, China

Z. Chen

Guangdong Research Center of Organoid Engineering and Technology

Guangzhou, 510535, Guangdong, China.

H. Yu

Department of Pharmacy

Shenzhen Qianhai Taikang Hospital

Shenzhen,518000, Guangdong, China.

H. Zhang, C. Wang, J. Fan, Z. Chen and H. Yu contributed equally to this work.

*Corresponding author: S. Qi; Department of Neurosurgery, Institute of Brain Diseases, Nanfang Hospital, Southern Medical University, Guangzhou 510515, Guangdong, China

Email: qisongtaonfyy@126.com

Y. Liu; Department of Neurosurgery, Institute of Brain Diseases, Nanfang Hospital, Southern Medical University, Guangzhou 510515, Guangdong, China

Email: liuyi818@smu.edu.cn

**This word file includes:**

**Figure S1 to Figure S14**

Figure S1 Overview of clinical information of ACP patients and the impact of cryopreservation on ACP PDOs.

Figure S2 Wet keratin/ghost cells in ACP PDOs and corresponding parental tumor tissues.

Figure S3 Deconvolution analysis of ACP PDO samples.

Figure S4 Comparison of transcriptional levels of the FGF family, BMP family, TGF family, WNT signaling pathway and SHH signaling pathway in ACP PDOs and ACP tissues.

Figure S5 Mutation spectrum of tissue and PDO of ACP 51, 57, and 59.

Figure S6 *CTNNB1* mutation analysis of ACP PDOs and corresponding parental tumors.

Figure S7 Drug sensitivity test of targeted drugs on ACP PDOs.

Figure S8 The main target of Ceritinib on ACP PDOs is IGF-1R.

Figure S9 Ceritinib promotes cell cycle arrest and apoptosis in STAM4 cells.

Figure S10 The effect of Ceritinib on the mRNA expression levels of *PIK3CA*, *AKT1* and *CTNNB1*.

Figure S11 Multi-planar (sagittal, coronal, and axial) and multi-slice contrast-enhanced T1-weighted magnetic resonance imaging (MRI) scans of Patient 1 over the follow-up period before second surgical resection.

Figure S12 Multi-planar (sagittal, coronal, and axial) and multi-slice contrast-enhanced T1-weighted MRI scans of Patient 2 over the follow-up period.

Figure S13 Multi-planar (axial, and coronal) and multi-slice T2-weighted MRI scans of Patient 2 over the follow-up period.

Figure S14 Analysis of the characteristics of whorl-like epithelium.

**Table S1 to Table S4**

Table S1 The siRNA sequences used in this study.

Table S2 List of the drugs and compounds used in this study.

Table S3 List of the antibodies used in this study.

Table S4 Primers for Quantitative real-time PCR used in this study.


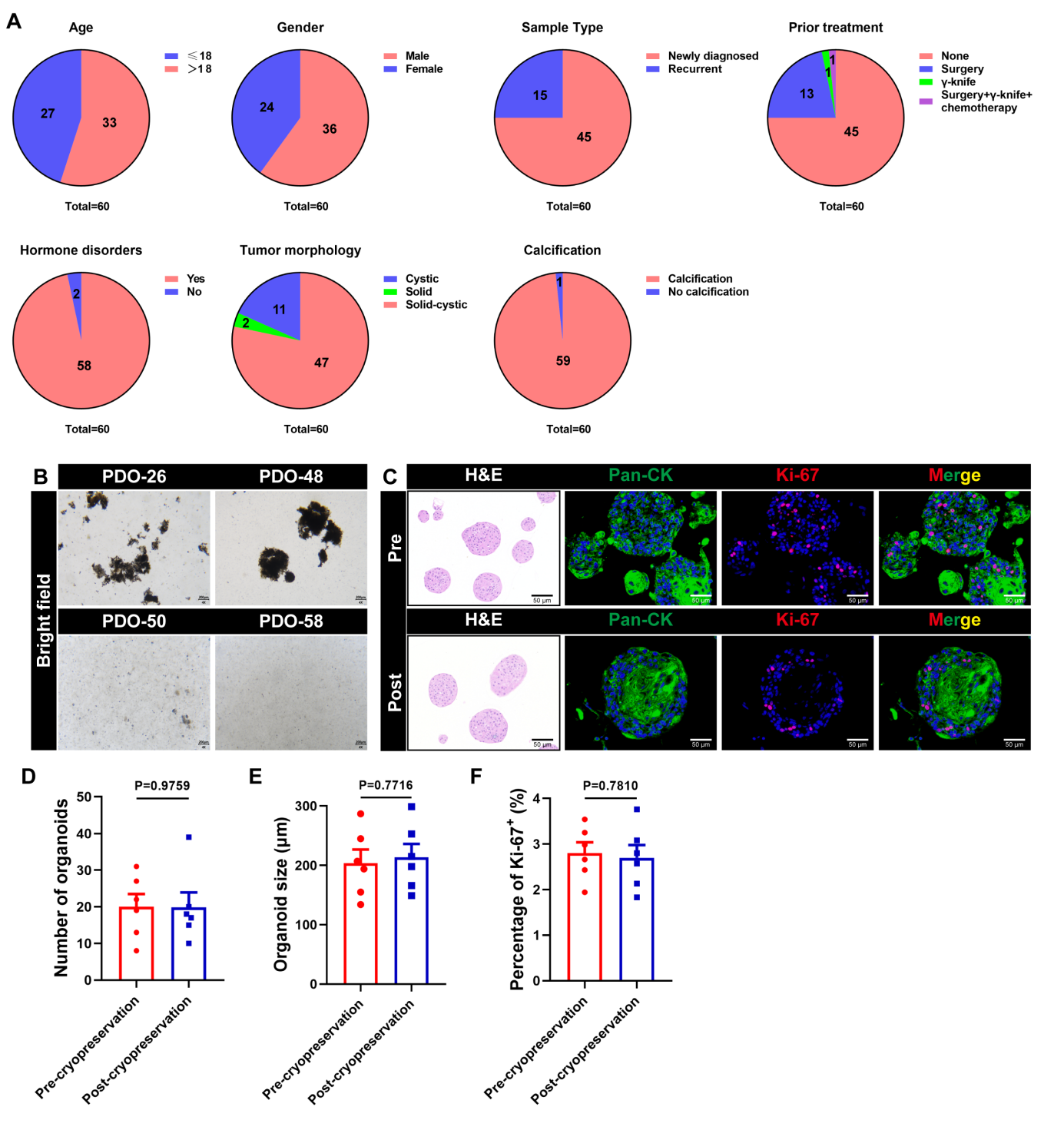


**Figure S1. Overview of clinical information of ACP patients and the impact of cryopreservation on ACP PDOs.** A) Establishment of patient-derived organoid (PDO) models of ACP (including establishment successes and failures) from various ages, genders, sample types, prior treatments, hormone disorders, tumor morphologies, and calcification statuses. B) Representative bright field micrographs of PDO_26, PDO_48, PDO_50, and PDO_58 that failed to establish the ACP organoid models. The image shows that no PDOs were formed after seven days of cultivation, and only a large amount of calcified crystals or impurities were present. C) Hematoxylin and eosin (H&E) staining, and dual immunofluorescence staining of pan Cytokeratin (Pan-CK) and Ki-67 of ACP PDOs before (pre-cryopreservation, upper) or after cryopreservation (post-cryopreservation, lower), representative images are shown. D-E) Quantification of the number (D) and diameter (E) of ACP PDOs before (pre-cryopreservation) or after cryopreservation (post-cryopreservation) (n=6/group). F) Quantification of the proliferation index Ki-67 of ACP PDOs before (pre-cryopreservation) or after cryopreservation (post-cryopreservation) (n=6/group). In all graphs, data are presented as mean ± standard error of mean (SEM). Data between two groups are compared by an independent-sample two-tailed Student’s *t*-test for (D)-(F). **p* < 0.05, ***p* < 0.01, ****p* < 0.001, ns: not significant.


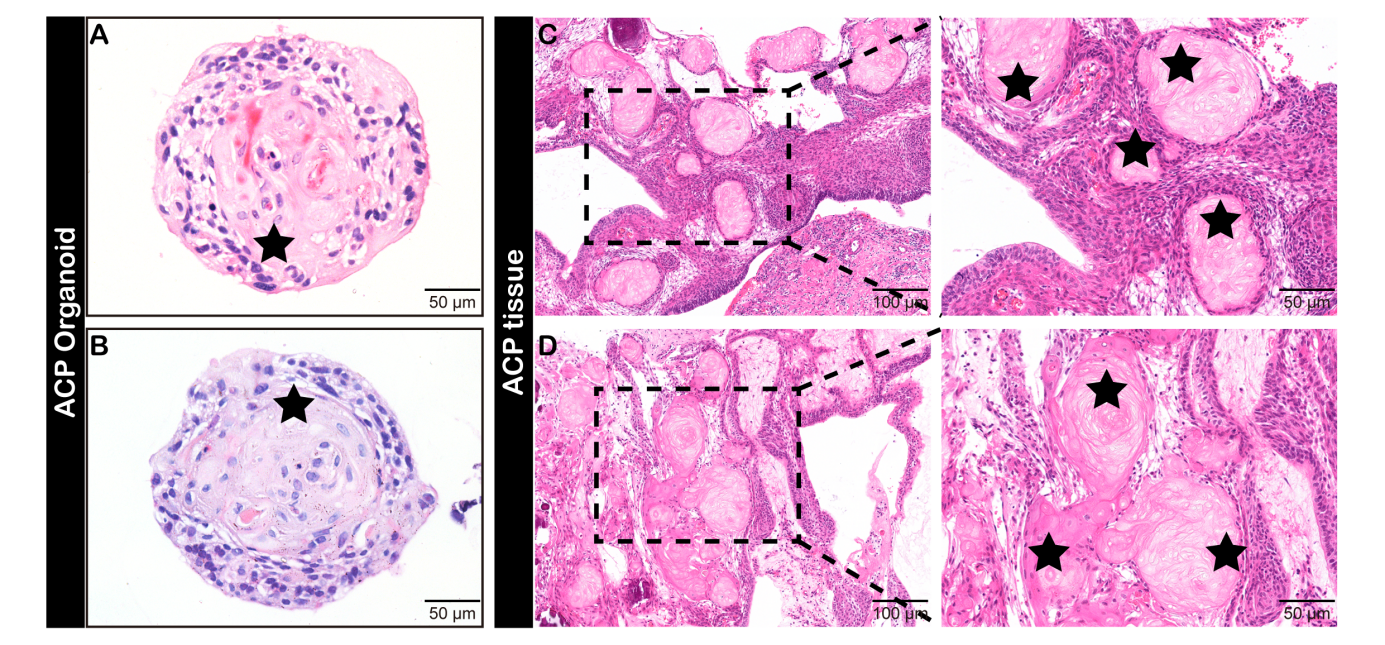


**Figure S2.** **Wet keratin/ghost cells in ACP PDOs and corresponding parental tumor tissues.** A, B) Representative images of H&E staining of ACP PDOs showing the structures similar to wet keratin/ghost cells that existed in ACP tissues. C, D) Representative images of H&E staining of ACP tissues showing the wet keratin/ghost cells. The black stars represent wet keratin/ghost cells.


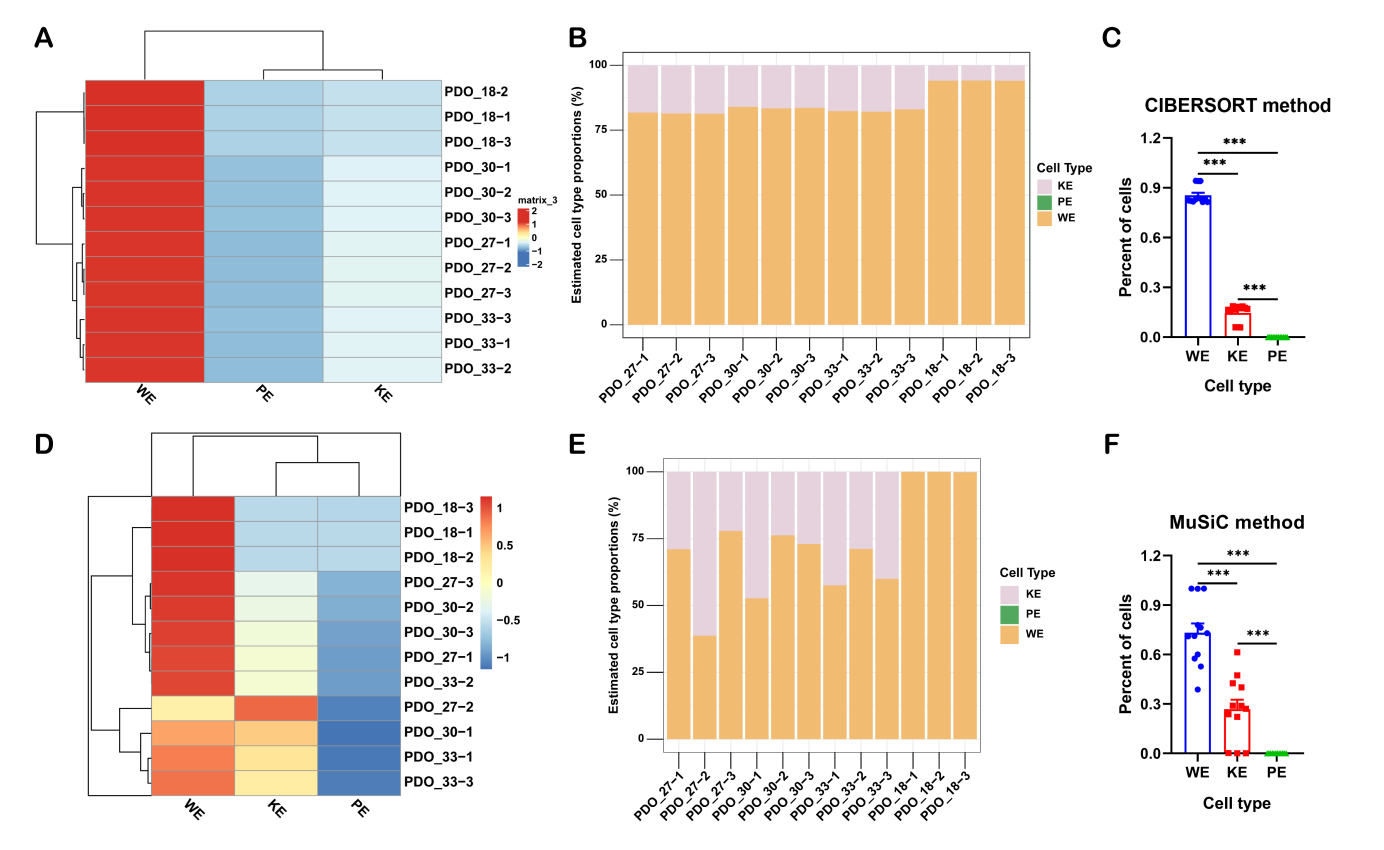


**Figure S3.** **Deconvolution analysis of ACP organoid samples.** A) Heatmap of the signature matrix of the 3 major cell types in 12 ACP organoid samples by Cell-type Identification By Estimating Relative Subsets Of RNA Transcripts (CIBERSORT) deconvolution method. B) Bar plot of the estimated cell type proportions by CIBERSORT deconvolution method for 12 ACP organoid samples. C) The estimated proportion of whorl-like epithelium (WE) was significantly higher than that of keratinized-like epithelium (KE) and palisade-like epithelium (PE) by CIBERSORT deconvolution method (n=12/group). D) Heatmap of the signature matrix of the 3 major cell types in 12 ACP organoid samples by MUlti-Subject SIngle Cell deconvolution (MuSiC) deconvolution method. E) Bar plot of the estimated cell type proportions by MuSiC deconvolution method for 12 ACP organoid samples. F) The estimated proportion of WE was significantly higher than that of KE and PE by MuSiC deconvolution method (n=12/group). In all graphs, data are presented as mean ± SEM. Data among three groups are compared by an one-way ANOVA test followed by a Tukey post hoc test for (C) and (F). **p* < 0.05, ***p* < 0.01, ****p* < 0.001, ns: not significant.


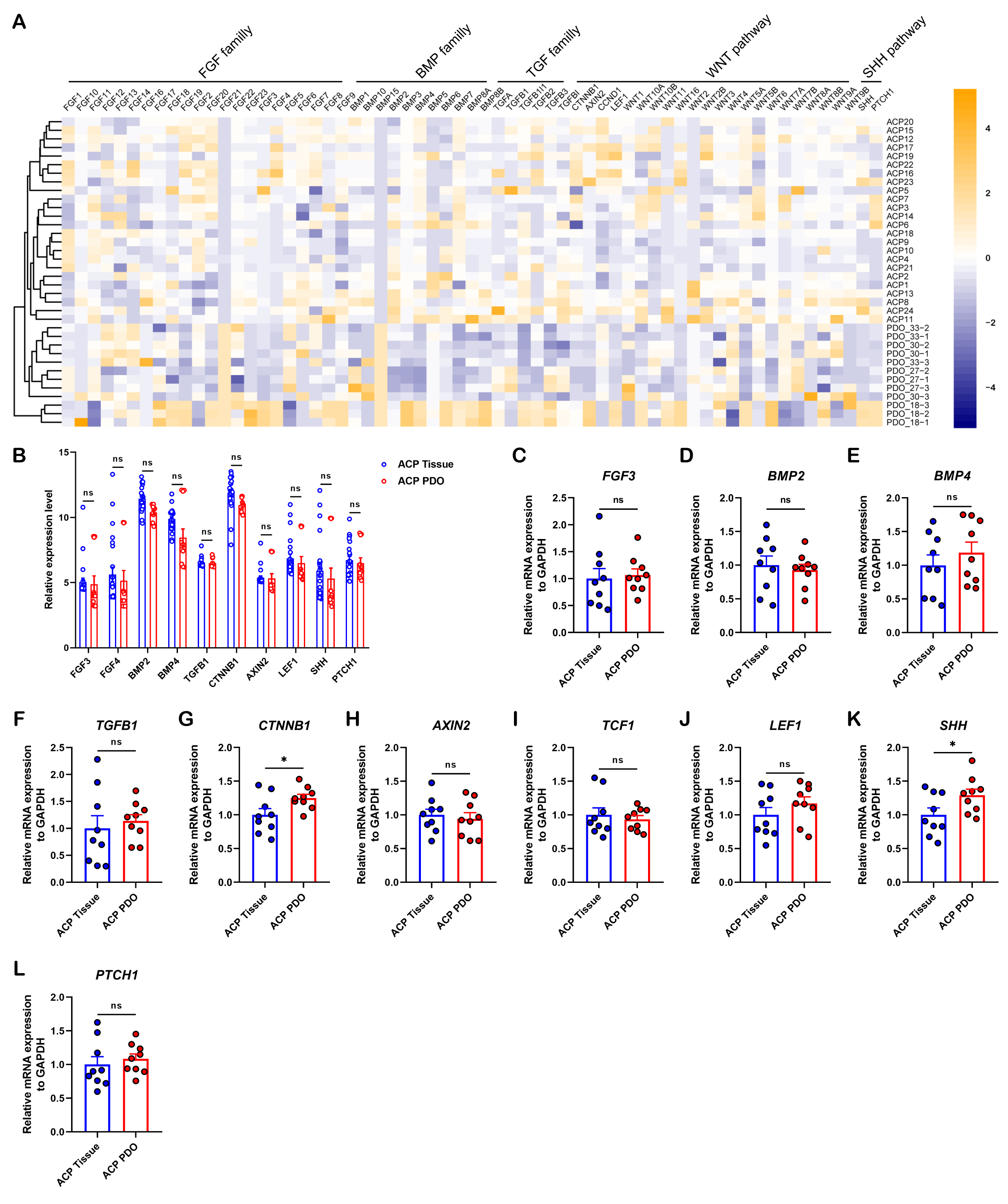


**Figure S4. Comparison of transcriptional levels of the FGF family, BMP family, TGF family, WNT signaling pathway and SHH signaling pathway in ACP PDOs and primary ACP tissues.** A) Comparison of relative transcriptional levels of the FGF, BMP, TGF families, WNT, and SHH signaling pathways in ACP PDOs (n=12) and primary ACP tissues (n=24) using standardized bulk RNA-sequencing data. B) Comparison of expression levels of *FGF3*, *FGF4*, *BMP2*, *BMP4*, *TGFB1*, *CTNNB1*, *AXIN2*, *LEF1*, *SHH*, and *PTCH1* in ACP PDOs (n=12) and primary ACP tissues (n=24) using original bulk RNA-sequencing data. C-L) Relative mRNA expression levels of *FGF3* (C), *BMP2* (D), *BMP4* (E), *TGFB1* (F), *CTNNB1* (G), *AXIN2* (H), *TCF1* (I), *LEF1* (J), *SHH* (K), and *PTCH1* (L) in ACP PDOs and primary ACP tissues (n=9/group). In all graphs, data are presented as mean ± SEM. Data among three groups are compared by an one-way ANOVA test followed by a Tukey post hoc test for (B). Data between two groups are compared by an independent-sample two-tailed Student’s *t*-test for (C)-(L). **p* < 0.05, ***p* < 0.01, ****p* < 0.001, ns: not significant.


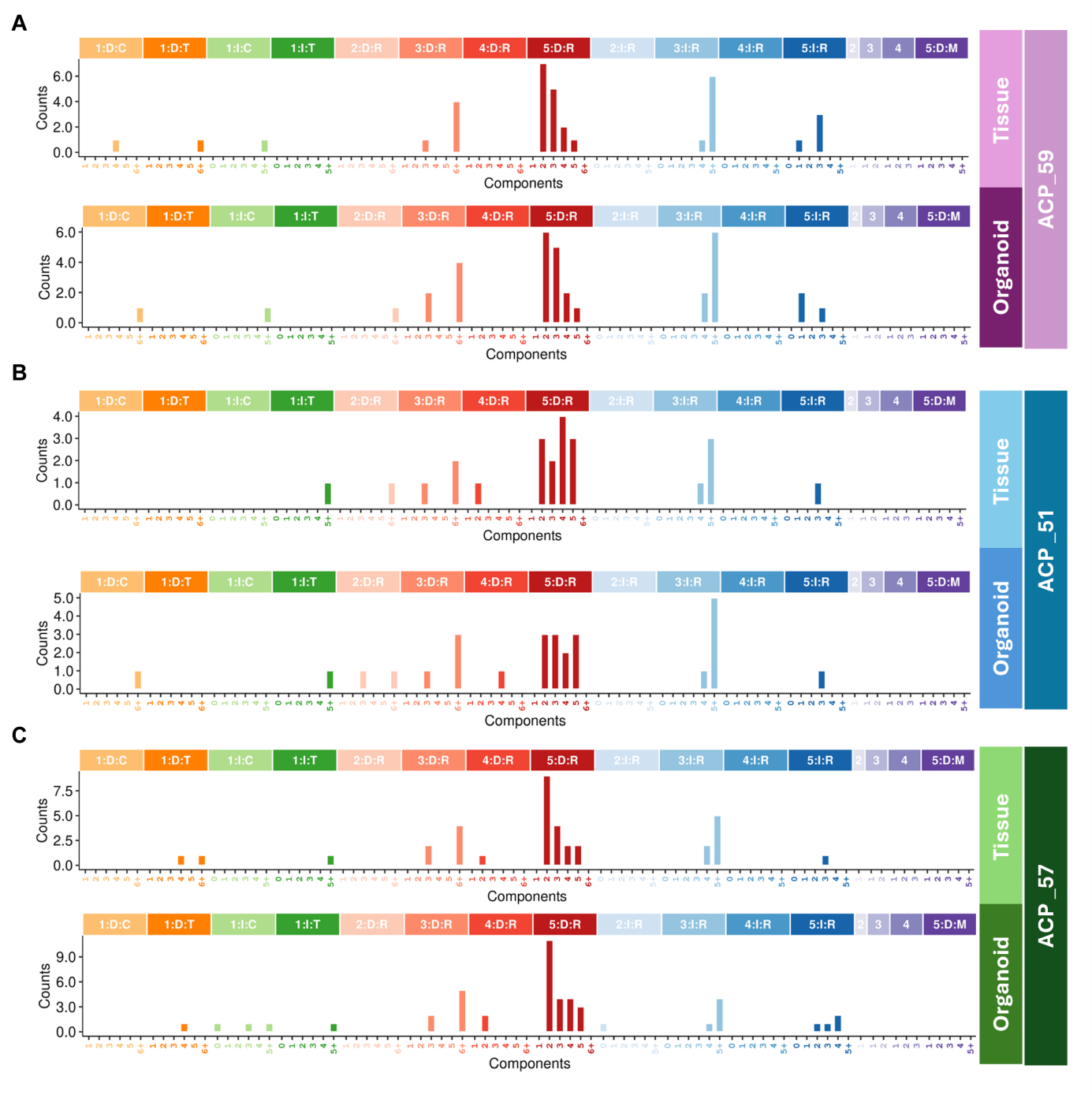


**Figure S5.** **Mutation spectrum of tissue and organoid of ACP 51, 57, and 59.** A-C) Mutation spectrum of tissue and organoid of ACP_59 (A), ACP_51 (B), and ACP_57 (C). The proportional contribution of deconvoluted mutational signatures in PDOs and their matched primary ACP tissues. Signatures were extracted using Sigminer from samples harboring ≥50 somatic single-nucleotide variants (SNVs). The figure enables visual comparison of signature proportions, demonstrating the concordance in underlying mutational processes between PDOs and primary tumors.

**
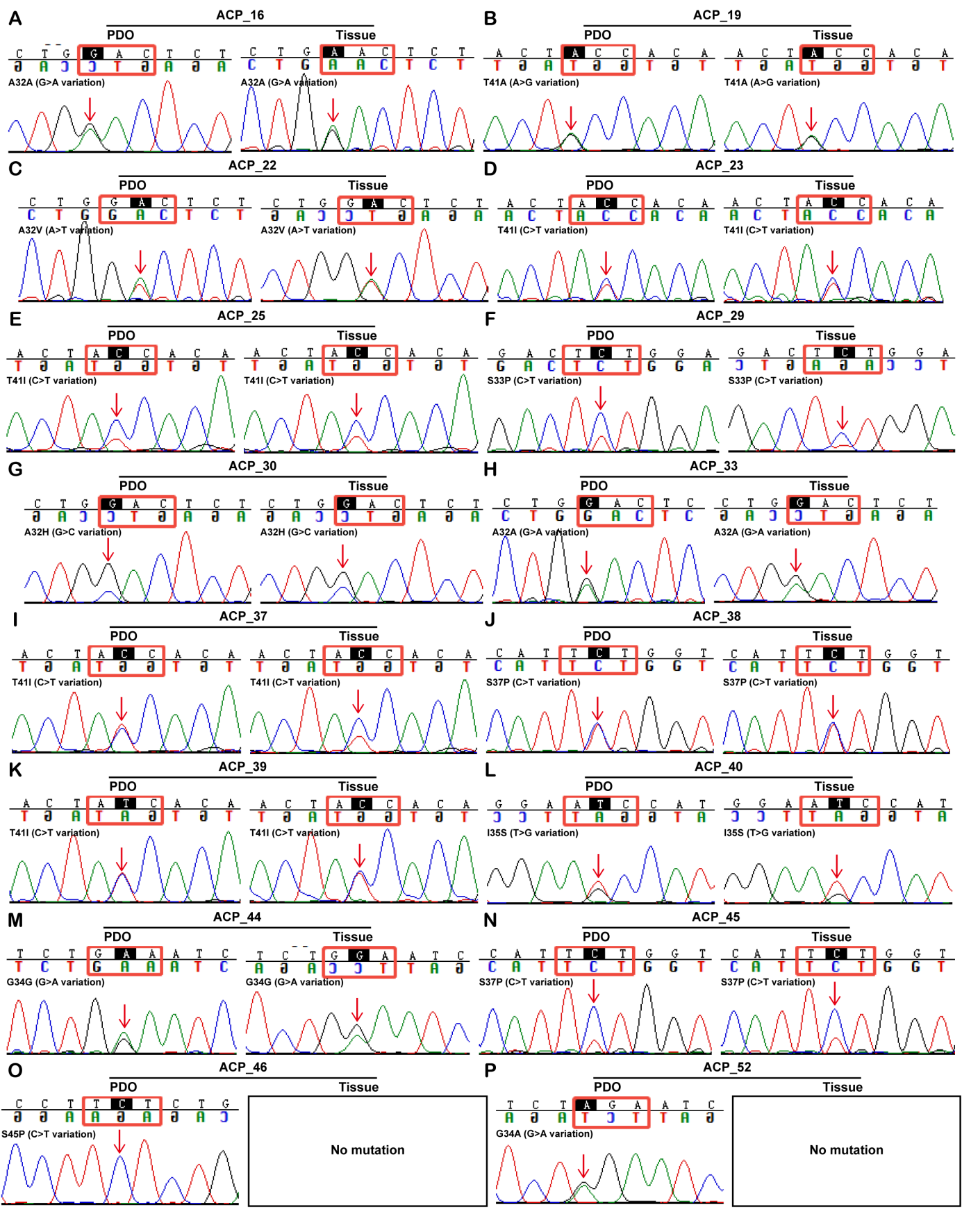
**

**Figure S6.** ***CTNNB1* mutation analysis of ACP PDOs and corresponding parental tumors.** A) Sanger sequencing chromatogram showing a typical point mutation in the *CTNNB1* gene (Asp32Ala) in PDOs and parental tumor tissue from ACP_16. B) Sanger sequencing chromatogram showing a typical point mutation in the *CTNNB1* gene (Thr41Ala) in PDOs and parental tumor tissue from ACP_19. C) Sanger sequencing chromatogram showing a typical point mutation in the *CTNNB1* gene (Asp32Val) in PDOs and parental tumor tissue from ACP_22. D) Sanger sequencing chromatogram showing a typical point mutation in the *CTNNB1* gene (Thr41Ile) in PDOs and parental tumor tissue from ACP_23. E) Sanger sequencing chromatogram showing a typical point mutation in the *CTNNB1* gene (Thr41Ile) in PDOs and parental tumor tissue from ACP_25. F) Sanger sequencing chromatogram showing a typical point mutation in the *CTNNB1* gene (Ser33Phe) in PDOs and parental tumor tissue from ACP_29. G) Sanger sequencing chromatogram showing a typical point mutation in the *CTNNB1* gene (Asp32His) in PDOs and parental tumor tissue from ACP_30. H) Sanger sequencing chromatogram showing a typical point mutation in the *CTNNB1* gene (Asp32Asn) in PDOs and parental tumor tissue from ACP_33. I) Sanger sequencing chromatogram showing a typical point mutation in the *CTNNB1* gene (Thr41Ile) in PDOs and parental tumor tissue from ACP_37. J) Sanger sequencing chromatogram showing a typical point mutation in the *CTNNB1* gene (Ser37Phe) in PDOs and parental tumor tissue from ACP_38. K) Sanger sequencing chromatogram showing a typical point mutation in the *CTNNB1* gene (Thr41Ile) in PDOs and parental tumor tissue from ACP_39. L) Sanger sequencing chromatogram showing a typical point mutation in the *CTNNB1* gene (Ile35Ser) in PDOs and parental tumor tissue from ACP_40. M) Sanger sequencing chromatogram showing a typical point mutation in the *CTNNB1* gene (Gly34Glu) in PDOs and parental tumor tissue from ACP_44. N) Sanger sequencing chromatogram showing a typical point mutation in the *CTNNB1* gene (Ser37Phe) in PDOs and parental tumor tissue from ACP_45. O) Sanger sequencing chromatogram showing a typical point mutation in the *CTNNB1* gene (Ser45Phe) in PDOs from ACP_46. P) Sanger sequencing chromatogram showing a typical point mutation in the *CTNNB1* gene (Gly34Arg) in PDOs from ACP_52.


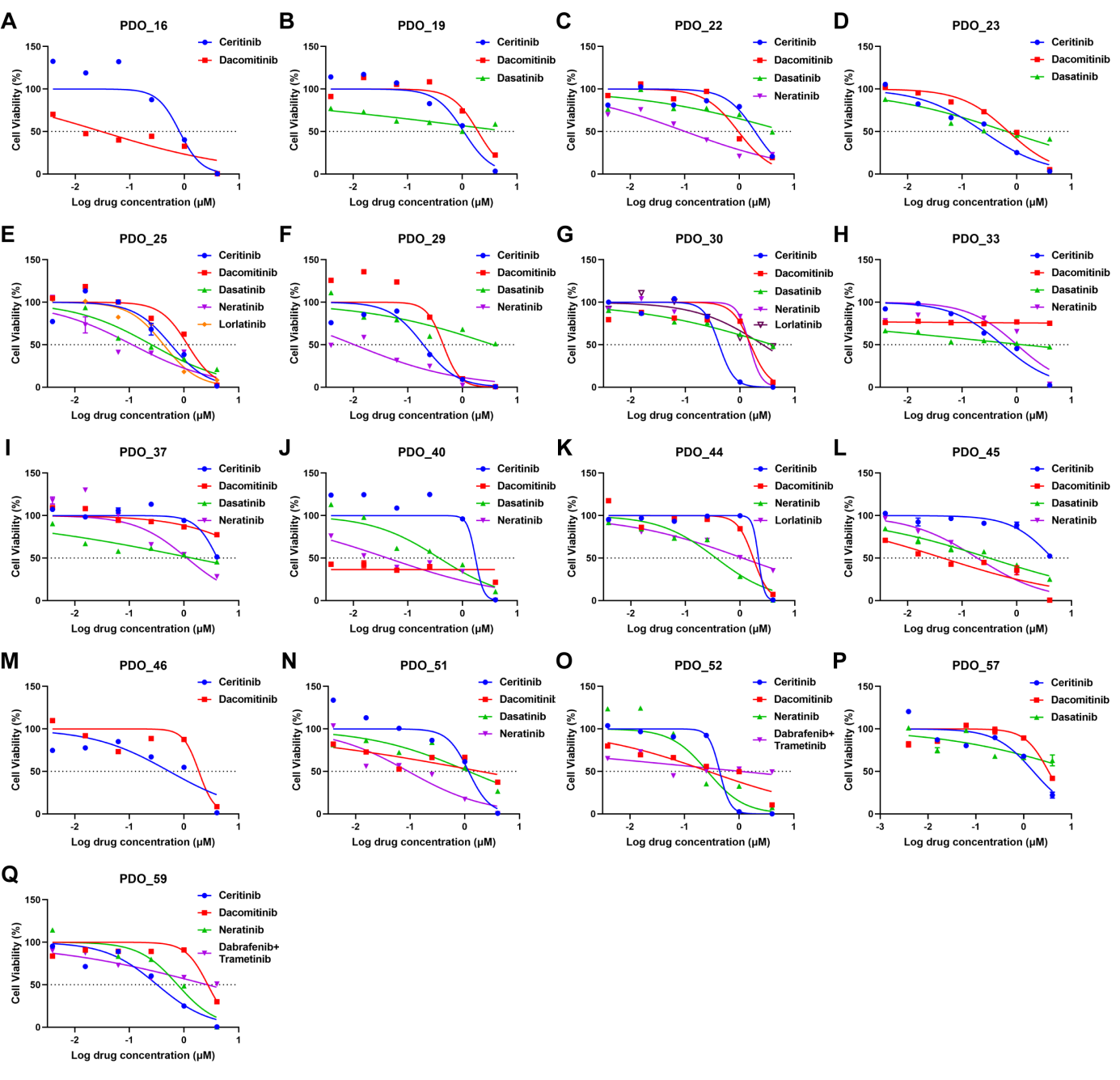


**Figure S7. Drug sensitivity test of targeted drugs on ACP PDOs.** A-Q) Dose response curves for PDO_16 (A), PDO_19 (B), PDO_22 (C), PDO_23 (D), PDO_25 (E), PDO_29 (F), PDO_30 (G), PDO_33 (H), PDO_37 (I), PDO_40 (J), PDO_44 (K), PDO_45 (L), PDO_46 (M), PDO_51 (N), PDO_52 (O), PDO_57 (P), and PDO_59 (Q) organoids treated with the indicated drugs individually or in combination (n=3/group). In all graphs, data are presented as mean ± SEM.


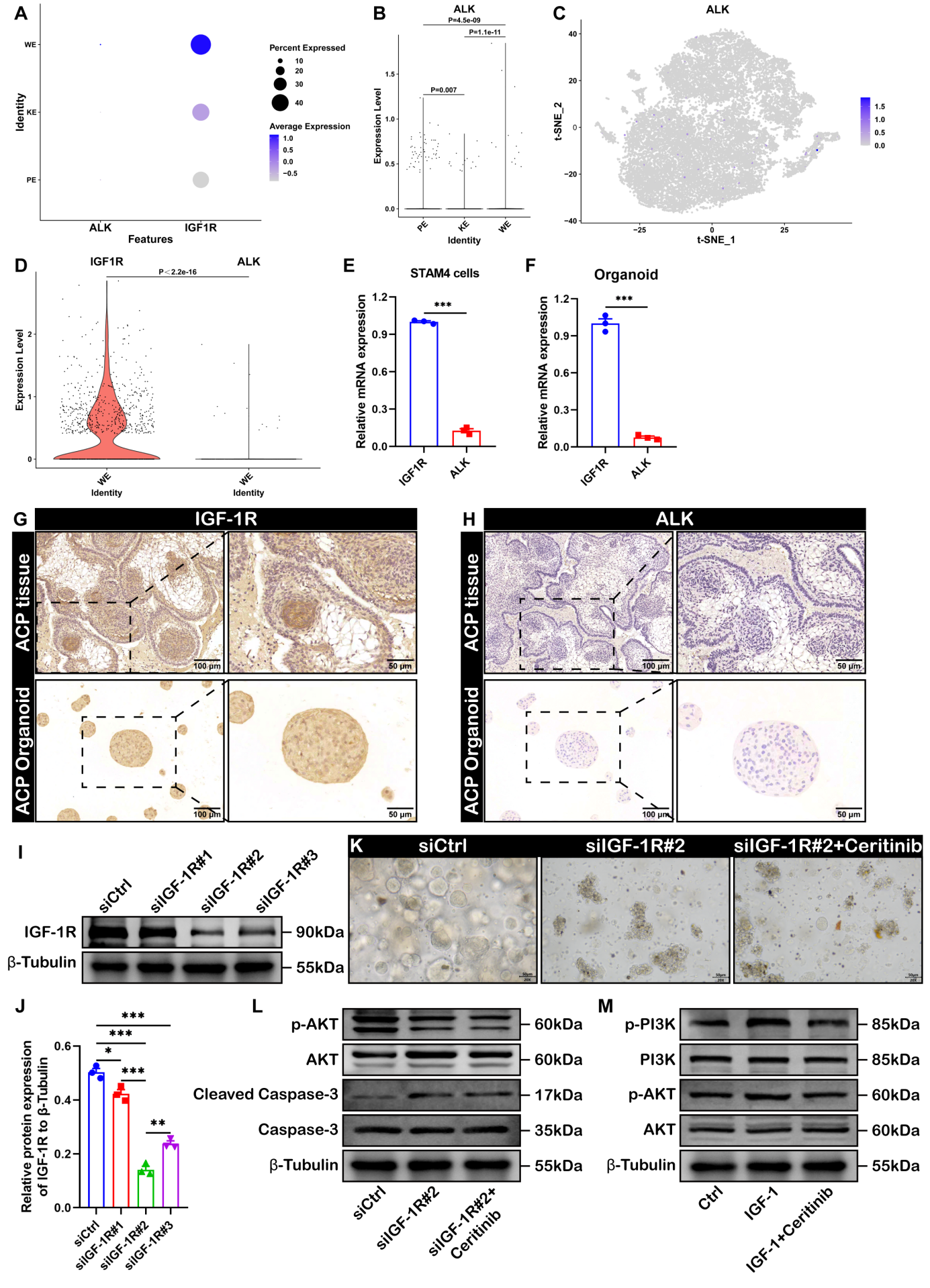


**Figure S8. The main target of Ceritinib on ACP PDOs is IGF-1R.** A) Bubble diagram showing the expression of ALK and insulin-like growth factor 1 receptor (IGF1R) among the three cell types. B) The violin plot comparing the expression values of ALK in WE, KE and PE in ACP tissues (n=1066 for WE group, n=8199 for KE group, n=13551 for PE group). C) t-distributed stochastic neighbor embedding (t-SNE) clustering plot showing the expression of ALK among the three cell types. D) The violin plot comparing the expression values of ALK and IGF1R in WE of ACP tissues (n=1066/group). E, F) Relative mRNA expression levels of *IGF1R* and *ALK* in STAM4 cells (E) and ACP PDOs (F) (n=3/group). G, H) Immumohistochemical staining of IGF-1R (G) and ALK (H) of ACP tissue samples (upper) or ACP PDOs (lower), representative images are shown. Boxed area is enlarged and presented on the right. I, J) The protein expression of IGF-1R in ACP PDOs treated with or without IGF-1R small interfering RNA (siRNA) were analysed by Western blot, and the results were represented as the expression of IGF-1R normalized to the expression of β-Tubulin (n=3/group). K) Representative bright-field microscopy images showing the morphological changes of ACP PDOs between siCtrl, siIGF-1R#2 and siIGF-1R#2+2μM Ceritinib group after 7 days of treatment. L) Detection of phosphorylation-AKT (p-AKT), AKT, Cleaved Caspase-3 and Caspase-3 protein expression in ACP PDOs treated with or without siIGF-1R#2, siIGF-1R#2+2μM Ceritinib. M) Detection of p-PI3K, PI3K, p-AKT, AKT protein expression in ACP PDOs treated with or without 100 ng/mL recombinant IGF-1 protein, 100 ng/mL IGF-1 protein+2μM Ceritinib. In all graphs, data are presented as mean ± SEM. Data among three or four groups are compared by an one-way ANOVA test followed by a Tukey post hoc test for (B) and (J). Data between two groups are compared by an independent-sample two-tailed Student’s *t*-test for (D)-(F). **p* < 0.05, ***p* < 0.01, ****p* < 0.001, ns: not significant.


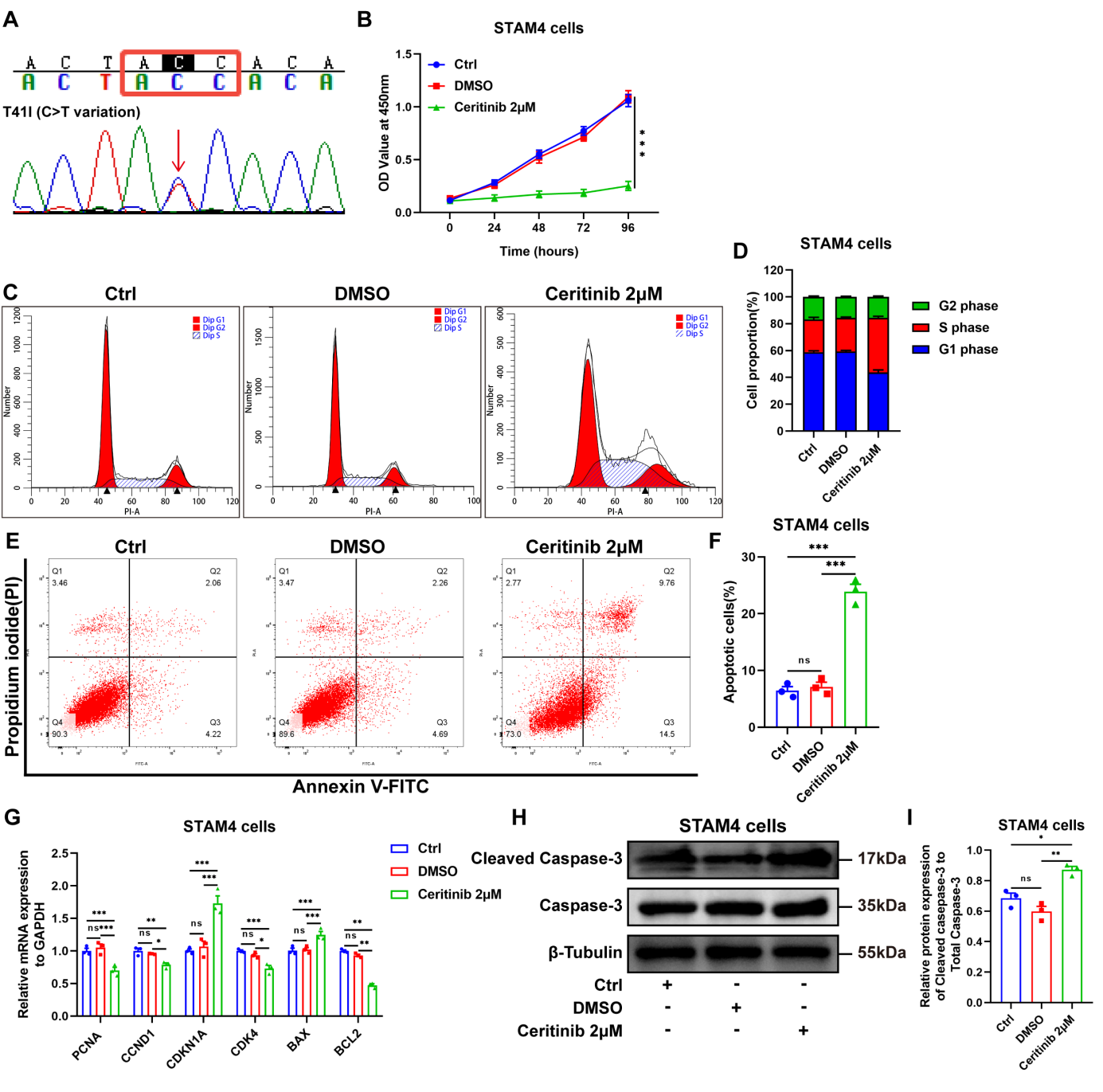


**Figure S9. Ceritinib promotes cell cycle arrest and apoptosis in STAM4 cells.** A) Sanger sequencing chromatogram showing a typical point mutation in the *CTNNB1* gene (Thr41Ile) in STAM4 cells. B) CCK-8 proliferation assay was used for detecting the proliferation ability of STAM4 cells treated with or without 2μM Ceritinib for 24hours, 48 hours, 72 hours, or 96 hours (n=3/group). C) The STAM4 cells were treated with or without 2μM Ceritinib for 48 hours, and the cell cycle distribution was assessed after propidium iodide (PI) staining by flow cytometry. D) Quantification of the cell cycle distribution of STAM4 cells treated with or without 2μM Ceritinib shown in (C) (n=3/group). E) The STAM4 cells were treated with or without 2μM Ceritinib for 48 hours, the cells were then harvested and stained with PI and annexin V-FITC for apoptotic analysis by flow cytometry. F) Quantification of the proportion of apoptotic cells of STAM4 cells treated with or without 2μM Ceritinib shown in (E) (n=3/group). G) Relative mRNA expression levels of *PCNA*, *CCND1*, *CDKN1A*, *CDK4*, *BAX*, and *BCL2* in STAM4 cells treated with or without 2μM Ceritinib (n=3/group). H, I) The protein expression of Cleaved Caspase-3 in STAM4 cells treated with or without 2μM Ceritinib were analysed by Western blot, and the results were represented as the expression of Cleaved Caspase-3 normalized to the expression of total Caspase-3 (n=3/group). In all graphs, data are presented as mean ± SEM. Data among three groups are compared by an one-way ANOVA test followed by a Tukey post hoc test for (F) and (I), and compared by a two-way ANOVA test followed by a Tukey post hoc test for (B) and (G). **p* < 0.05, ***p* < 0.01, ****p* < 0.001, ns: not significant. DMSO, dimethylsulfoxide; PI, propidium iodide; FITC, fluorescein isothiocyanate.


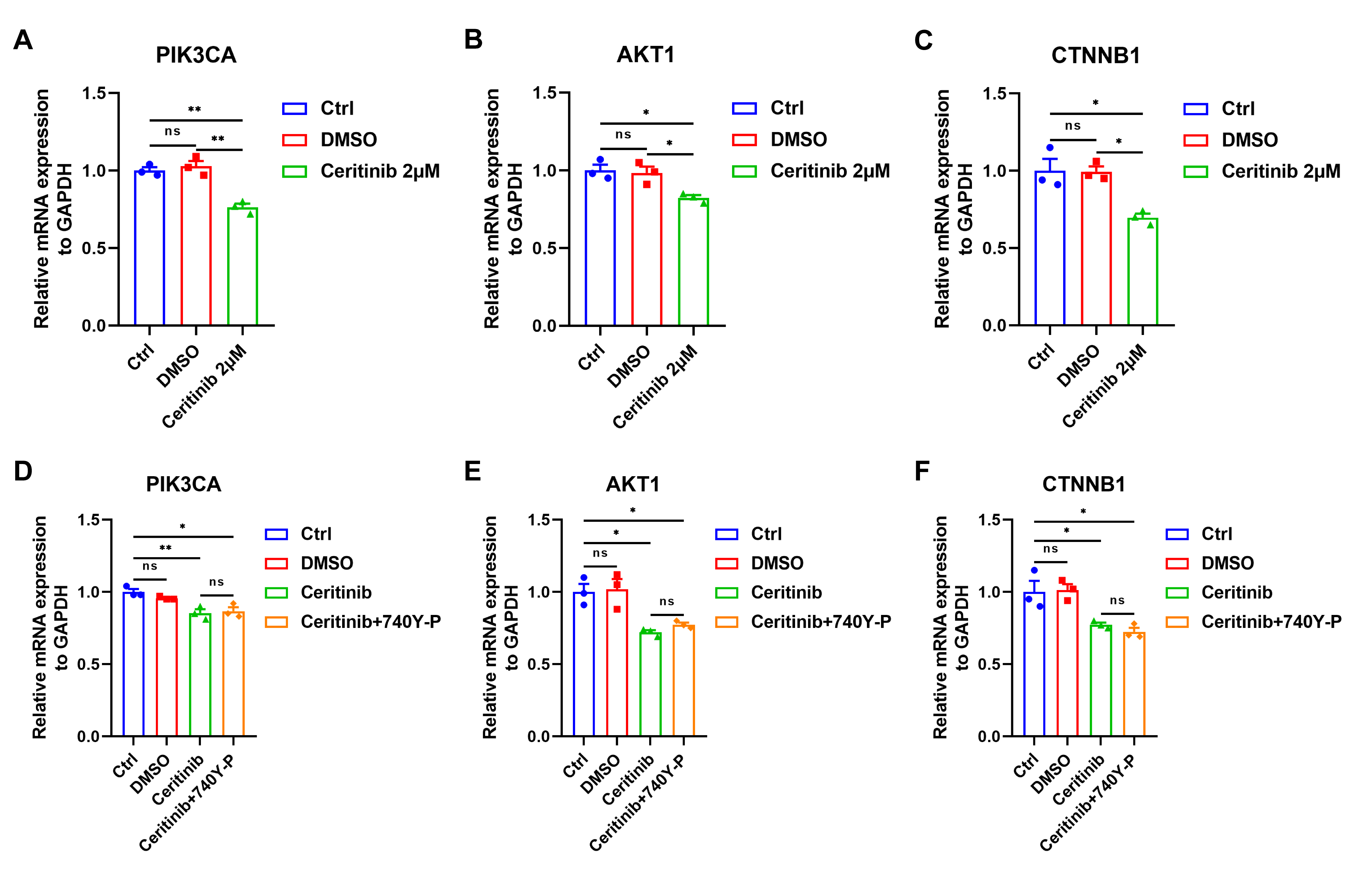


**Figure S10. The effect of Ceritinib on the mRNA expression levels of *PIK3CA*, *AKT1* and *CTNNB1*.** A-C) Relative mRNA expression levels of *PIK3CA* (A), *AKT1* (B), and *CTNNB1* (C) in ACP PDOs treated with or without 2μM Ceritinib (n=3/group). D-F) Relative mRNA expression levels of *PIK3CA* (D), *AKT1* (E), and *CTNNB1* (F) in ACP PDOs treated with or without 2μM Ceritinib, 2μM Ceritinib+30μg/ml 740Y-P (n=3/group). In all graphs, data are presented as mean ± SEM. Data among three or four groups are compared by an one-way ANOVA test followed by a Tukey post hoc test for (A-F). **p* < 0.05, ***p* < 0.01, ****p* < 0.001, ns: not significant.


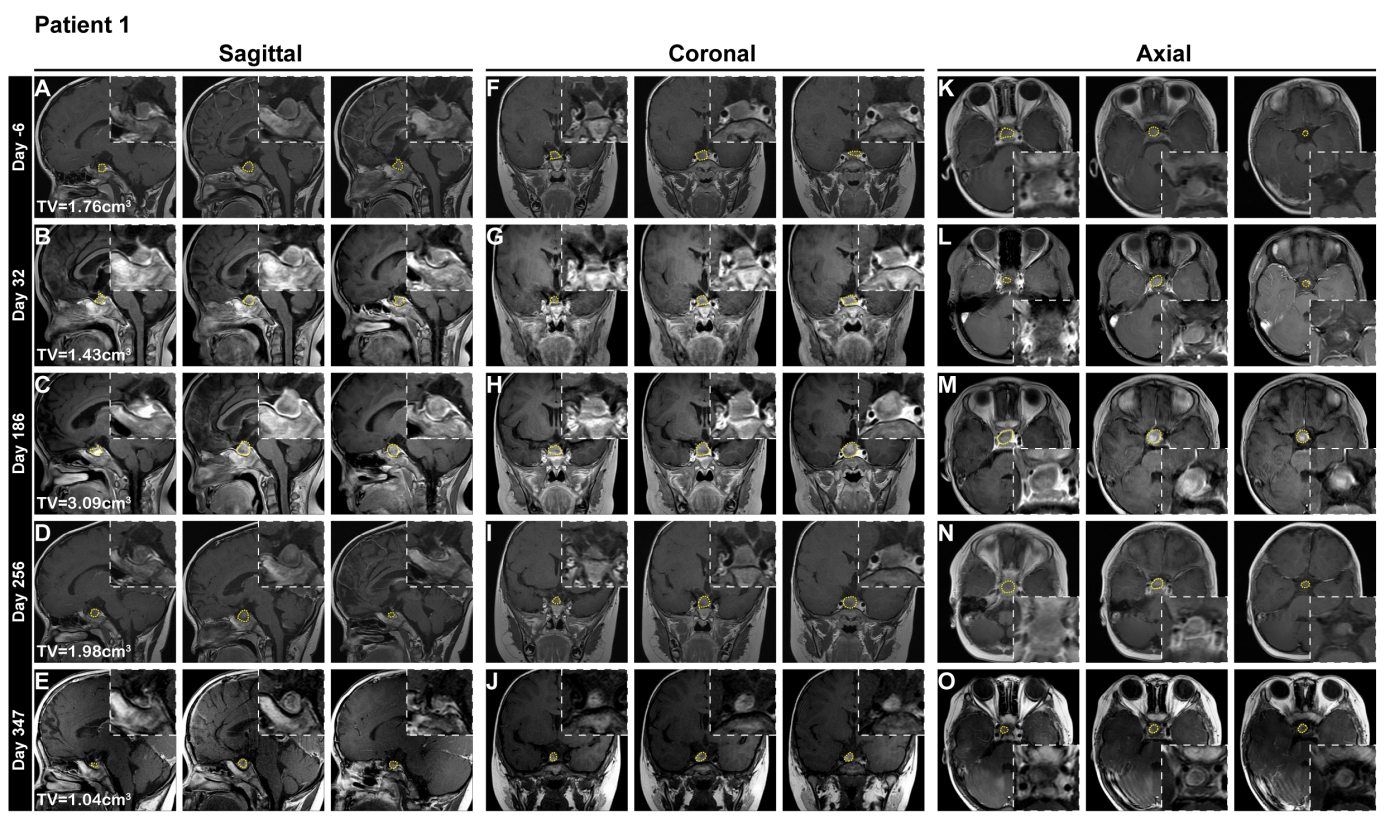


**Figure S11. Multi-planar (sagittal, coronal, and axial) and multi-slice contrast-enhanced T1-weighted magnetic resonance imaging (MRI) scans of Patient 1 over the follow-up period before second surgical resection.** A-E) The sequential sagittal contrast-enhanced T1-weighted MRI scans of ACP patient 1, spanning from initial diagnosis to the most recent follow-up before second surgical resection. The magnified image of the tumor is presented on the right. Medical therapy with Ceritinib was started on Day 0. F-J) The sequential coronal contrast-enhanced T1-weighted MRI scans of ACP patient 1, spanning from initial diagnosis to the most recent follow-up before second surgical resection. The magnified image of the tumor is presented on the right. Medical therapy with Ceritinib was started on Day 0. K-O) The sequential axial contrast-enhanced T1-weighted MRI scans of ACP patient 1, spanning from initial diagnosis to the most recent follow-up before second surgical resection. The magnified image of the tumor is presented on the right. Medical therapy with Ceritinib was started on Day 0. TV, Tumor volume.


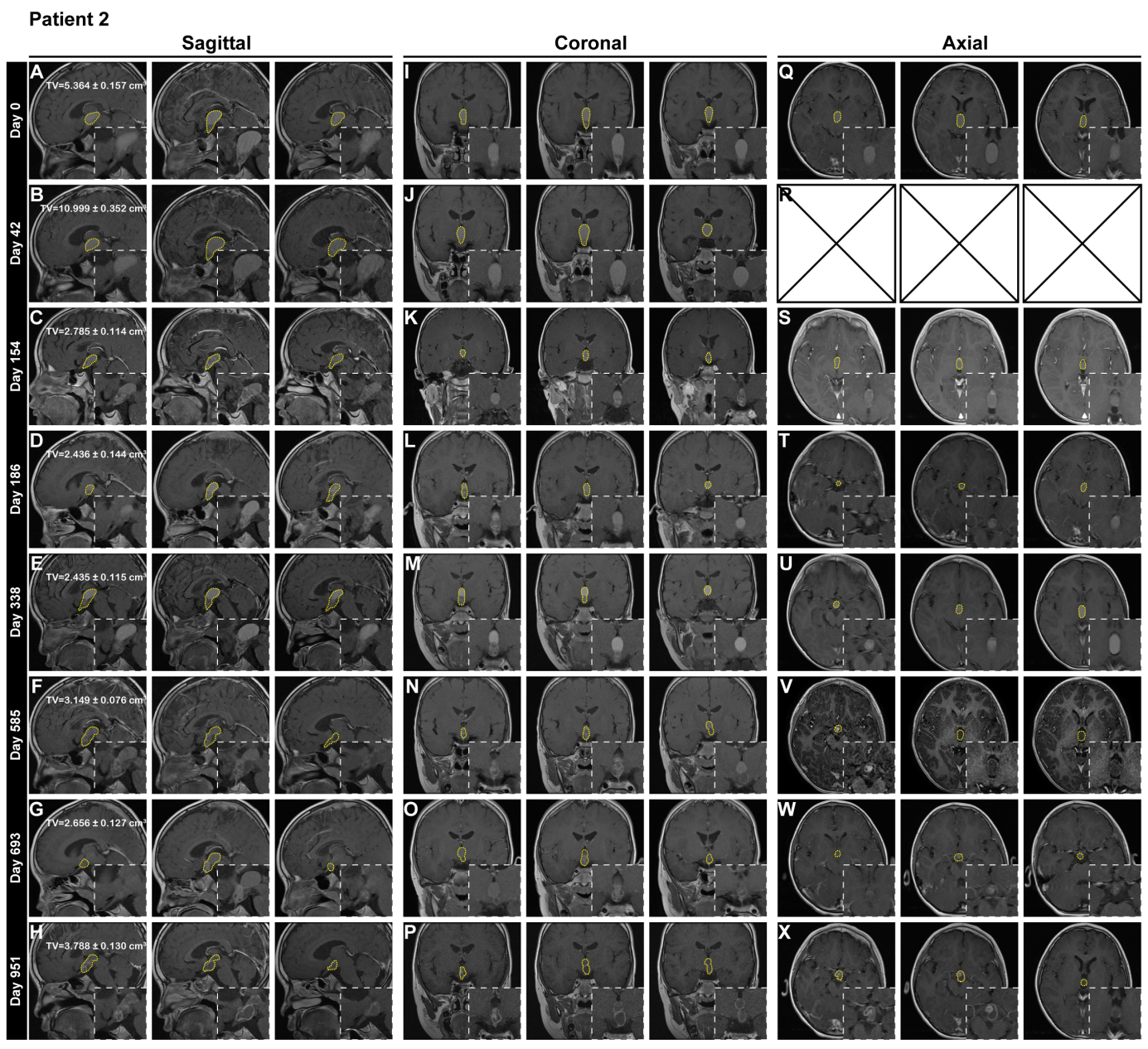


**Figure S12. Multi-planar (sagittal, coronal, and axial) and multi-slice contrast-enhanced T1-weighted MRI scans of Patient 2 over the follow-up period.** A-H) The sequential sagittal contrast-enhanced T1-weighted MRI scans of ACP patient 2, spanning from initial diagnosis to the most recent follow-up. The magnified image of the tumor is presented on the right. Medical therapy with Ceritinib was started on Day 0. I-P) The sequential coronal contrast-enhanced T1-weighted MRI scans of ACP patient 2, spanning from initial diagnosis to the most recent follow-up. The magnified image of the tumor is presented on the right. Medical therapy with Ceritinib was started on Day 0. Q-X) The sequential axial contrast-enhanced T1-weighted MRI scans of ACP patient 2, spanning from initial diagnosis to the most recent follow-up. The magnified image of the tumor is presented on the right. Medical therapy with Ceritinib was started on Day 0. In all graphs, data are presented as mean ± SEM. TV, Tumor volume.

**
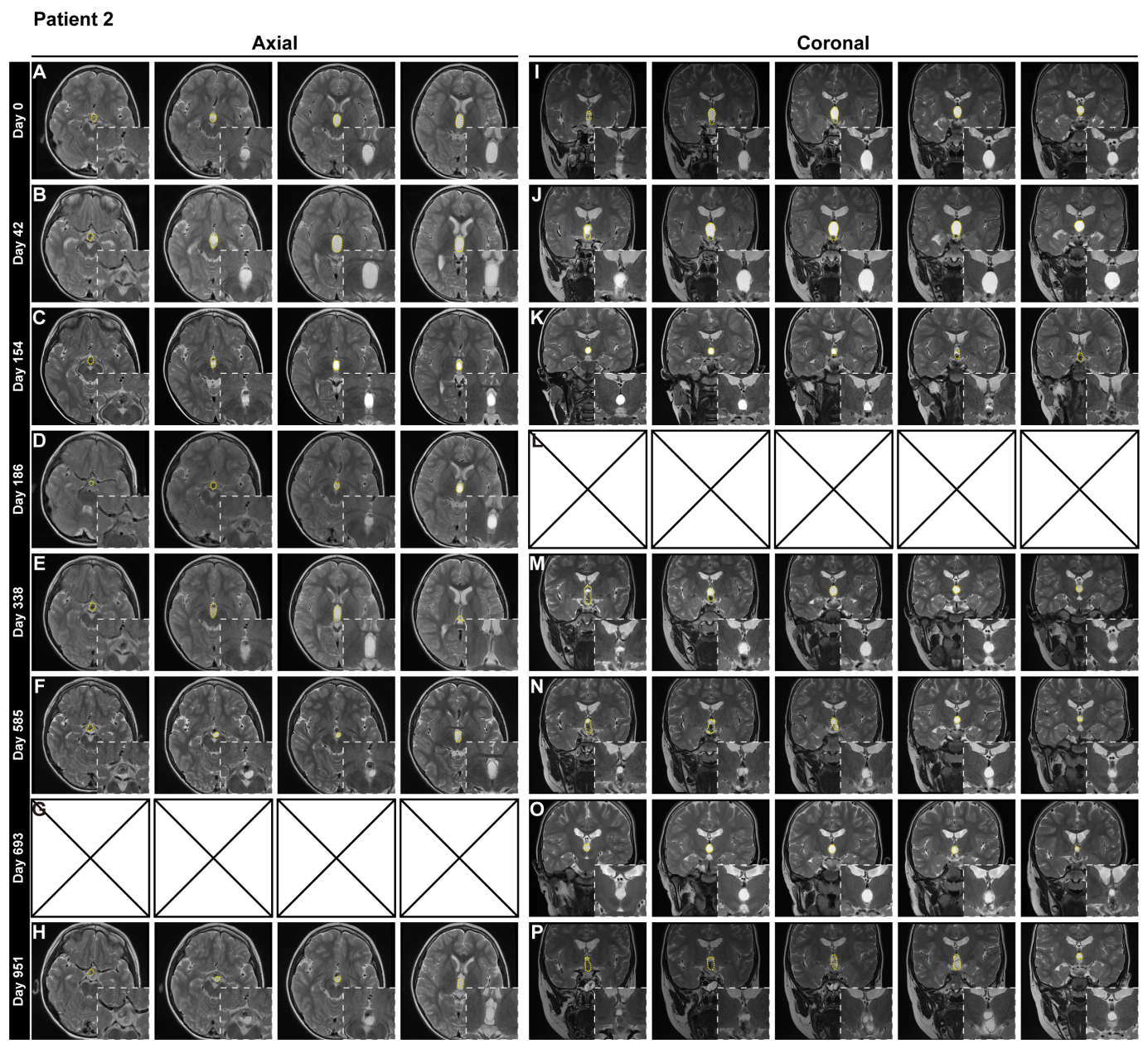
**

**Figure S13. Multi-planar (axial, and coronal) and multi-slice T2-weighted MRI scans of Patient 2 over the follow-up period.** A-H) The sequential axial T2-weighted MRI scans of ACP patient 2, spanning from initial diagnosis to the most recent follow-up. The magnified image of the tumor is presented on the right. Medical therapy with Ceritinib was started on Day 0. I-P) The sequential coronal T2-weighted MRI scans of ACP patient 2, spanning from initial diagnosis to the most recent follow-up. The magnified image of the tumor is presented on the right. Medical therapy with Ceritinib was started on Day 0.


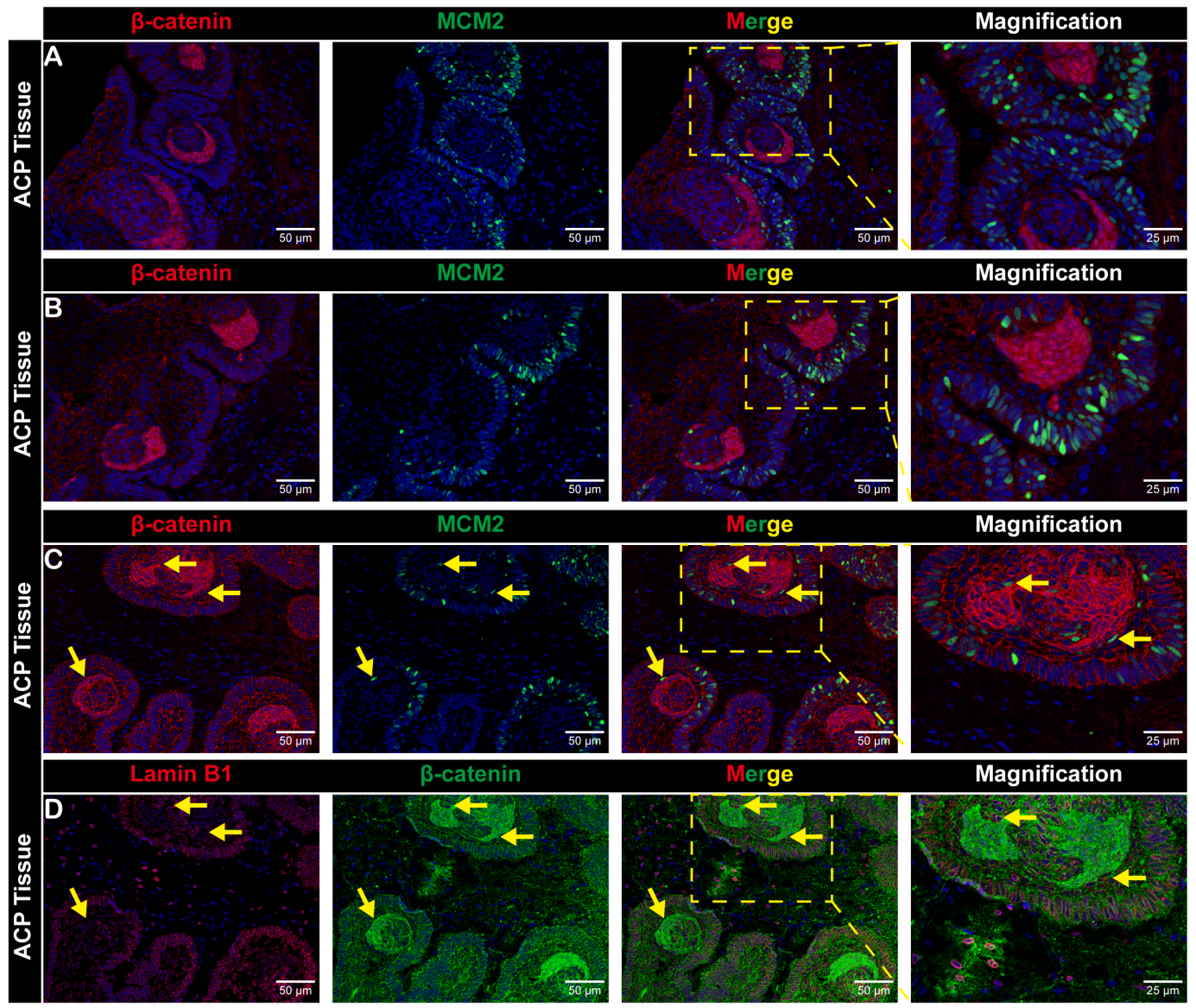


**Figure S14. Analysis of the characteristics of whorl-like epithelium.** A-C) Dual immunofluorescence staining of MCM2 and β-catenin of ACP tissues, representative images are shown. Boxed area is enlarged and presented on the right. D) Dual immunofluorescence staining of Lamin B1 and β-catenin of ACP tissues, representative images are shown. Boxed area is enlarged and presented on the right.

**Table S1** The siRNA sequences used in this study.

| siRNAs | Sequence |
| --- | --- |
| siIGF-1R#1 | 5′-AAGGATATTGGGCTTTACAACCTGATT-3′ |
| siIGF-1R#2 | 5'-AGGCGGGCTTCCGGGAGGTCTCCTT-3' |
| siIGF-1R#3 | 5'-GGACGAGATGGAGGCGGGCTTCCGG-3' |
| Scramble siRNA | 5'-GGAAGAGGTGGAGGCTCGCTCGCGG-3' |

**Table S2** List of the drugs and compounds used in this study.

| **Number** | **Drug Name** | **Target** | **Company** | **Catalog Number** |
| --- | --- | --- | --- | --- |
| 1 | Ceritinib (LDK378) | ALK, IGF-1R | MedChemExpress | HY-15656 |
| 2 | Dacomitinib (PF-00299804) | EGFR, HER2 | MedChemExpress | [HY-13272](https://www.medchemexpress.cn/Dacomitinib.html) |
| 3 | Dasatinib (BMS-354825) | Src, Bcr-Abl | MedChemExpress | HY-10181 |
| 4 | Napabucasin (BBI608) | STAT3 | MedChemExpress | HY-13919 |
| 5 | Neratinib (HKI-272) | EGFR, HER2 | MedChemExpress | HY-32721 |
| 6 | Lorlatinib (PF-06463922) | ALK | MedChemExpress | HY-12215 |
| 7 | Hydroxychloroquine sulfate (HCQ sulfate) | TLR7/9 | MedChemExpress | HY-B1370 |
| 8 | Dabrafenib (GSK2118436A) | Raf | MedChemExpress | HY-14660 |
| 9 | Trametinib (GSK1120212; JTP-74057) | MEK | MedChemExpress | HY-10999 |
| 10 | Tofacitinib (CP-690550) | JAK3/2/1 | MedChemExpress | HY-40354 |
| 11 | Vismodegib (GDC-0449) | SHH | MedChemExpress | HY-10440 |
| 12 | Recombinant insulin-like growth factor-1 (IGF-1) protein | IGF-1R | MedChemExpress | HY-P7018 |
| 13 | 740Y-P | PI3K agonist | Selleck | S7865 |

**Table S3** List of the antibodies used in this study.

| **Antibody** | **Company** | **Catalog Number** | **Dilution** | **Assay** |
| --- | --- | --- | --- | --- |
| Mouse monoclonal anti-pan Cytokeratin (pan-CK) | Abcam | Cat# ab7753; RRID: AB_306047 | 1:500 | Immunofluorescence |
| Mouse monoclonal anti-β-catenin | Abcam | Cat# ab22656; RRID: AB_447227 | 1:500, 1:1000 | Immunofluorescence, Western blotting |
| Mouse monoclonal anti-CD44 | Abcam | Cat# ab254530; RRID: AB_2885131 | 1:500 | Immunofluorescence |
| Rabbit polyclonal anti-Ki-67 | Abcam | Cat# ab15580; RRID: AB_443209 | 1:2000 | Immunofluorescence |
| Rabbit monoclonal anti-EGFR | Abcam | Cat# ab52894; RRID: AB_869579 | 1:250 | Immunofluorescence |
| Rabbit monoclonal anti-Phospho-p44/42 MAPK (Erk1/2) (Thr202/Tyr204) | Cell Signaling Technology | Cat# 4370; RRID: AB_2315112 | 1:200 | Immunofluorescence |
| Rabbit monoclonal anti-CD133 | Abcam | Cat# ab222782; RRID: AB_3065213 | 1:500 | Immunofluorescence |
| Rabbit monoclonal anti-KLF4 | Abcam | Cat# ab215036; RRID: AB_2933978 | 1:100 | Immunofluorescence |
| Rabbit monoclonal anti-Notch 1 | Abcam | Cat# ab52627; RRID: AB_881725 | 1:100 | Immunofluorescence |
| Rabbit monoclonal anti-Sonic Hedgehog (SHH) | Abcam | Cat# ab53281; RRID: AB_882648 | 1:250 | Immunofluorescence |
| Mouse monoclonal anti-p16 INK4a | Santa Cruz | Cat# sc-1661; RRID: AB_628067 | 1:50/1:200 | Immunofluorescence |
| Rabbit monoclonal anti-IGF-1 Receptor (IGF-1R) | Abcam | Cat# ab182408; RRID: AB_3106875 | 1:1000 | Immunofluorescence  Immunohistochemistry |
| Rabbit polyclonal anti-IGF1R | Affinity | Cat# AF6125; RRID: AB_2835009 | 1:1000 | Western blotting |
| Rabbit monoclonal anti-Anaplastic Lymphoma Kinase (ALK) | Abcam | Cat# ab16670; RRID: AB_443426 | 1:200 | Immunohistochemistry |
| Rabbit polyclonal anti-Phospho-PI3K p85 alpha (Tyr607) | Affinity | Cat# AF3241; RRID: AB_2834667 | 1:1000 | Western blotting |
| Rabbit polyclonal anti-PI3K p85 alpha | Affinity | Cat# AF6241; RRID: AB_2835340 | 1:1000 | Western blotting |
| Rabbit monoclonal anti-Phospho-Akt (Ser473) | Cell Signaling Technology | Cat# 4060; RRID: AB_2315049 | 1:2000 | Western blotting |
| Rabbit monoclonal anti-Akt (pan) (C67E7) | Cell Signaling Technology | Cat# 4691; RRID: AB_915783 | 1:1000 | Western blotting |
| Rabbit polyclonal anti-Phospho-GSK-3β (Ser9) | Cell Signaling Technology | Cat# 9336; RRID: AB_331405 | 1:1000 | Western blotting |
| Rabbit monoclonal anti-GSK-3β (27C10) | Cell Signaling Technology | Cat# 9315; RRID: AB_490890 | 1:1000 | Western blotting |
| Rabbit monoclonal anti-Cleaved Caspase-3 (Asp175) | Cell Signaling Technology | Cat# 9664; RRID: AB_2070042 | 1:1000 | Western blotting |
| Rabbit polyclonal anti-Caspase-3 | Cell Signaling Technology | Cat# 9662; RRID: AB_331439 | 1:1000 | Western blotting |
| Rabbit monoclonal anti-α-Tubulin | Cell Signaling Technology | Cat# 2125; RRID: AB_2619646 | 1:1000 | Western blotting |
| Rabbit monoclonal anti-Lamin B1 | Abcam | Cat# ab133741; RRID: AB_2616597 | 1:5000 | Western blotting |
| Goat anti-Mouse IgG (H+L) Cross-Adsorbed Secondary Antibody, Alexa Fluor 488 | Thermo Fischer Scientific | Cat# A-11001; RRID: AB_2534069 | 1:1000 | Immunofluorescence |
| Goat anti-Rabbit IgG (H+L) Cross-Adsorbed Secondary Antibody, Alexa Fluor 594 | Thermo Fischer Scientific | Cat# A-11012; RRID: AB_2534079 | 1:1000 | Immunofluorescence |
| Goat anti-Rat IgG (H+L) Cross-Adsorbed Secondary Antibody, Alexa Fluor 488 | Thermo Fischer Scientific | Cat# A-11006; RRID: AB_2534074 | 1:1000 | Immunofluorescence |
| Donkey anti-Goat IgG (H+L) Cross-Adsorbed Secondary Antibody, Alexa Fluor 488 | Thermo Fischer Scientific | Cat# A-11055; RRID: AB_2534102 | 1:1000 | Immunofluorescence |
| HRP-conjugated goat anti-mouse IgG antibody | Jackson ImmunoResearch | Cat # 115-035-003; RRID: AB_10015289 | 1:5000 | Western blotting |
| HRP-conjugated goat anti-rabbit IgG antibody | Jackson ImmunoResearch | Cat# 111-035-003; RRID: AB_2313567 | 1:5000 | Western blotting |

**Table S4** Primers for Quantitative real-time PCR used in this study.

| **Genes** | **Forward primer (5’ to 3’)** | **Reverse primer (5’ to 3’)** |
| --- | --- | --- |
| *Homo AKT1* | TCTTTGCCGGTATCGTGT | TGTCATCTTGGTCAGGTGGT |
| *Homo PIK3CA* | CTTTTGGAGTCCTATTGTCGTG | CCTAGTTGATGAGCAGGGTTT |
| *Homo CTNNB1* | AAAGCGGCTGTTAGTCACTGG | CGAGTCATTGCATACTGTCCAT |
| *Homo PCNA* | CCTGCTGGGATATTAGCTCCA | CAGCGGTAGGTGTCGAAGC |
| *Homo CCND1* | CAATGACCCCGCACGATTTC | CATGGAGGGCGGATTGGAA |
| *Homo CDKN1A* | TGTCCGTCAGAACCCATGC | AAAGTCGAAGTTCCATCGCTC |
| *Homo CDK4* | TCAGCACAGTTCGTGAGGTG | GTCCATCAGCCGGACAACAT |
| *Homo BAX* | CCCGAGAGGTCTTTTTCCGAG | CCAGCCCATGATGGTTCTGAT |
| *Homo BCL2* | GACTTCGCCGAGATGTCCA | CCCCACCGAACTCAAAGAA |
| *Homo IGF1R* | GTACAACTACCGCTGCTGGACC | TACACAGGCCGTGTCGTTGTC |
| *Homo ALK* | agatctctgttcgagtccct | tctgtaaaccaggagccgta |
| *Homo LEF1* | CTGCTAGAGACGCTGATCCA | TGGCTCTTGCAGTAGACGAA |
| *Homo TCF1* | AGATCCTGTTCCAGGCCTAT | GGATGCATTCCGCCCTATT |
| *Homo AXIN2* | TGGCTATCTCCCCACCTTGA | CAGTTTCCGTGGACCTCACA |
| *Homo SHH* | AGAGGAGGCACCCCAAAAAG | TACACCTCTGAGTCATCAGCCT |
| *Homo PTCH1* | CCCCTGTACGAAGTGGACACTC | AAGGAAGATCACCACTACCTTGG |
| *Homo TGFB1* | GGATACCAACTATTGCTTCAGCTCC | AGGCTCCAAATATAGGGGCAGGGTC |
| *Homo BMP2* | ACTCGAAATTCCCCGTGACC | CCACTTCCACCACGAATCCA |
| *Homo BMP4* | AAAGTCGCCGAGATTCAGGG | CAGAAGTGTCGCCTCGAAGT |
| *Homo FGF3* | GGCGTCTACGAGCACCTTG | CACCTCCACTGCCGTTATCTC |
| *Homo GAPDH* | GGAGCGAGATCCCTCCAAAAT | GGCTGTTGTCATACTTCTCATGG |
